# Supplementary material for: Are most published research findings false? Trends in statistical power, publication selection bias, and the false discovery rate in psychology (1975–2017)
Source: PLoS One. 2023 Oct 17;18(10):e0292717. doi: 10.1371/journal.pone.0292717 (PMC10581498; doi:10.1371/journal.pone.0292717)
Supplement: S1 Appendix — (PDF) [file pone.0292717.s001.pdf]

Supporting Information

Are most published research findings false? Trends in statistical power, publication selection bias, and the false discovery rate in psychology (1975–2017)

Author: *Andreas Schneck[1]**

[1]Ludwig-Maximilians-University Munich

**Corresponding author*

Email: andreas.schneck@lmu.de.

1. Operationalization

In the following, the three measures, publication selection bias, statistical power, and the false discovery rate (FDR), are explained in more detail. Furthermore, the underlying assumptions of the calculations are laid out.

1.1. Statistical Power

To calculate the statistical power, it was necessary to assume an underlying true effect. As laid out in the main article, this is done by assuming an *a priori* plausible effect size. Therefore, Assumption A is:

The underlying true effect size is constant across psychology (e.g. sub-fields, sample sizes, primary but also secondary analyses)

Assumption A

For the *a priori* statistical power, all test statistics had to be converted in a common effect size, in this case in Cohen's *d* metric, to calculate the standard error of the empirical effect. The following formulas for balanced tests were used for Cohen's *d* (cp. 1: 27-29, without correction term using the effect size itself)[1] and for the product-moment (Pearson) correlation coefficient as well as the equivalent point-biserial correlation coefficient (1: 42, 2). In the case of Cohen's *d* all t-tests were

[1] Borenstein suggests $se_d = \sqrt{\frac{n_1+n_2}{n_1 \times n_2} + \frac{d^2}{2\times(n_1+n_2)}}$, dropping the second summand and assuming equal group sizes yields: $2/\sqrt{N}$

assumed as paired tests with a between-pairs correlation of $r = 0.5$, which yields: $\sigma = 1/\sqrt{N}$. This approach produces rather small and thus optimistic standard error estimates. Because of the pruned correction term, the standard error estimates for Cohen's d were also evaluated in a simulation study that compared the true standard error to the estimated standard error using the formulas presented in Table A.[2] The used formula slightly underestimates the standard error on average below 2 percent, and what is even more important, in no case overestimates it. Overall, consistent and conservative standard errors for r, t as well as χ^2 and F with only one degree of freedom could be obtained. Calculating the standard error based on multi-group comparisons (χ^2 and F-tests with a degree of freedom > 1) and generalizing over η^2 (3: 282), no consistent estimates could be obtained. For both χ^2 and F-test multi-group comparisons, the standard error is slightly overestimated and varies immensely. Therefore, multi-group comparisons were dropped from the analysis.

**Test value**	$\boldsymbol{\sigma_d}$	$\boldsymbol{\sigma_r}$
$\chi^2\ if\ df = 1$	$2/\sqrt{N}$	$\sqrt{1/(N-3)}$
$F\ if\ df_1 = 1$	$2/\sqrt{N}$	$\sqrt{1/(N-3)}$
r	$2/\sqrt{N}$	$\sqrt{1/(N-3)}$
t	$\sqrt{\left(\frac{1}{N}\right) \times (2 \times (1-r))}$	$\sqrt{1/(N-3)}$

Table A Transformation formulas for test statistics in Cohen's *d*

$\boldsymbol{\sigma_d}$ is the standard error of Cohen's d, $\boldsymbol{\sigma_r}$ the standard error of Pearson's correlation coefficient. N represents the total sample size for all groups.

1.2. Publication selection bias

Besides the statistical power, publication selection bias in the form of publication bias or p-hacking had to be computed. In the literature, meta-analyses concerning only one underlying effect model publication selection bias by including the studies' precision as an additional covariate (e.g., 4 on the effect of minimum wage on employment). However, this is not efficiently applicable to research areas with no or little cumulative research. The caliper test (CT) developed by Gerber and Malhotra (5, 6) allows detecting publication selection bias, in the form of publication bias or p-hacking under

[2] Control group normally distributed size N around mean = 0 and standard deviation = 1 (ϕ(0,1), test group 1 (two-group comparisons, df=1): ϕ(uniform(0.001,0.8),1), test group 2 (additionally for multigroup comparisons, df=2 and independently of test group 1): ϕ(uniform(0.001,0.8),1). All groups are of equal size *N*: (uniform(30,400)) and unpaired. 1,000 replications of the simulation were run.

effect heterogeneity using only the z-values (z) in a small band (called caliper, c) around a prespecified significance threshold (th).

As the CT ignores z-values outside the narrow range (th), it is not affected by effect heterogeneity that occurs when examining the whole test value distribution (for simulations, see 7). Effect heterogeneity in this context means that the studies differ by random variation and substantial differences that may be explained by different research topics or research designs (8). In the intervals around th, the upper interval that contains results that are just statistically significant (over-caliper, *CR*, $x_z = 1$) should be as likely as results that slightly miss statistical significance (under-caliper, *1-CR*, $x_z = 0$). The CT therefore follows the logic of a regression discontinuity design (9), examining a treatment effect (in this case, publication) that is caused by an assignment variable (in this case, statistical significance). A limitation on the narrow range is necessary because the functional form of the entire distribution is unknown (see 10 for a critique on using distributional assumptions). The only known characteristic of the z-statistic is its continuity over its complete range, meaning there should not be any abrupt jumps in the distribution. The narrower the bandwidth of the examined interval c around th, the more likely the assumption of an equal distribution due to continuity is met (cp. Equation A).

$$x_z = \begin{cases} 0 \; if \; th - c * th < |z| \leq th \\ 1 \; if \; th < |z| < th + c * th \end{cases}$$

Equation A

Gerber and Malhotra (5, 6) used a 5%, 10%, 15%, and 20% bandwidth c. The widest bandwidths may be biased because the 20%-CT around the 5%- and 10%- significance threshold overlap. Publication selection bias around the 5%- and 10%-significance level may cancel each other out in the case of the 20%-CT. In a simulation study that compared alternative tests on publication selection bias, a bandwidth of 5% the size of th (0.098 in case of the 5% significance threshold th = 1.96) proved adequate to absorb fluctuations in the underlying effect distribution (7). Choosing this bandwidth also included as many test values as possible to ensure an adequate statistical power of the CT. Although the large number of z-values in the study at hand provides the CT with sufficient statistical power, another reason to choose wider intervals is the accuracy of the test values "(e.g., $F(1,49) = 2.34$) reported in the primary studies that are usually presented with two decimal places and add further noise to the distribution.

The CT presented so far only allows testing for publication selection bias. However, using the results of the CT allows to at least approximate the possible publication selection bias rates for

both types, publication bias, and p-hacking. The risk of both forms only comes into play if a researcher obtains a nonsignificant result in the first place (cp. Fig A). If significant results were obtained, publication selection bias practices would simply not be needed. In other words, the researcher is not at risk of committing publication selection bias. In contrast, if there is a nonsignificant result, a researcher faces three options: either try to publish the result irrespective of its outcome, omit publication in the case of publication bias, or start searching for significant specifications in the case of p-hacking.

In the case of publication bias, because nonsignificant results drop out of the analysis, the rate of pure publication bias, given that no p-hacking is present, can be calculated under Assumption B.

Publication bias drops nonsignificant results at an equal rate for any p-value in the interval *(α, 1] and is therefore independent of the p-value obtained in the first place and other factors (e.g., whether H0 or H1 is true, sample size, etc.).*

Assumption B

P-hacking (phr) is defined as making a nonsignificant result significant conditional to the fact that the first p-value analyzed is nonsignificant. This, however, is more complex to model, as a nonsignificant result is not merely dropped but replaced with an iteratively generated significant result given the data at hand. To identify the p-hacking rate, θ, the probability of an underlying true effect (true H1), the statistical $power$, and the significance threshold α have to be known.

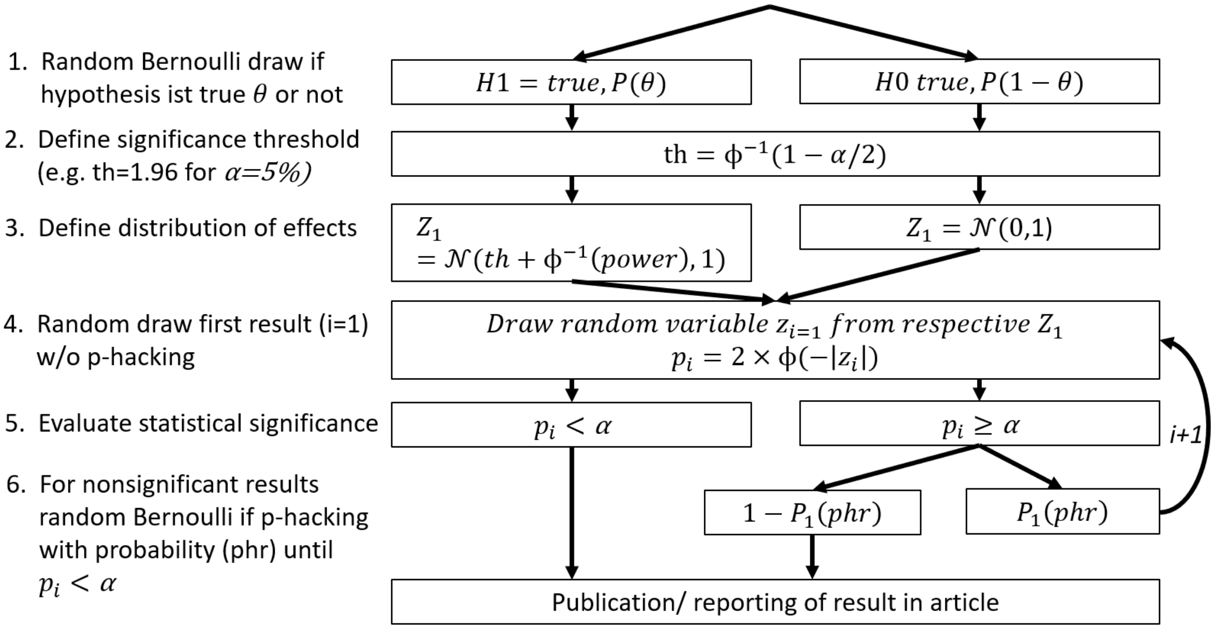

Fig A Process of p-hacking

The first step in the precondition that sets the stage for of p-hacking, as shown in Fig A θ defines if there is an underlying effect (H1 true) or no underlying effect (H0 true). At this step, which is out of the control of researchers, assumption C has to be made:

The probability that H1 is true is known (probability = θ) among all tests performed in the literature.

Assumption C

In the second step, the significance threshold is defined, in our case, the 5%-significance threshold th=1.96 is of most interest. Before drawing a *p*-value, the distribution of the test statistic has to be defined in step 3 by using the statistical power (in case of H1) and just a standard normal distribution around a null effect (in case of H0). Because in this setting, the average statistical power in psychology or a subdiscipline is used, the following Assumption D is made:

The actual statistical power (if p-hacking did not exist) is constant among all tests of the literature where H1 is true.

Assumption D

In step 4, the actual result z_i from the distribution Z_1 is drawn and converted into a *p*-value. The respective *p*-value is then in step 5 evaluated on its statistical significance ($p < \alpha$). In case of a significant effect, there is no need to invest in p-hacking and the *p*-value is reported or published as denoted in step 6. In the case of a nonsignificant *p*-value, p-hacking is implemented with a probability phr. Steps 4 to 5 are repeated until a significant result is obtained. This definition contains two additional Assumptions E and F.

The probability of p-hacking (phr) is independent of whether H0 or H1 is true.

Assumption E

If authors start p-hacking, they only stop if they obtain a significant p-value

Assumption F

To obtain the p-hacking (phr) estimate, the observed probability of just significant test values in the caliper, CR, is modeled and then solved for phr. For the estimation below, the definitions were laid out:

Definitions:

Conditional share of significant results:	$ar = \alpha$ OR $power$, depending if H0 or H1 is true
Significance threshold:	$th = \phi^{-1}(1 - \alpha/2)$
Critical value statistical power:	$z = \phi^{-1}(1 - power)$ OR $\phi^{-1}(1 - \alpha/2)$<br>, depending if H0 or H1 is true
Caliper band:	$c = th * caliper$

In the case of p-hacking, given the estimated CR, the statistical power and the assumed probability of a true hypothesis are known. Equation B shows the conditional probability of a just significant result ($p_{OC\_unbiased}$) on the z-statistic from the significant threshold to the upper bound of the over-caliper $(th, th + c]$ without any p-hacking. The same logic but in the different direction also applies to the just nonsignificant results (p_{UC}) in the under-caliper $(th - c, th]$ in Equation D, again without p-hacking. The probability of values in the over- and under-caliper accounts also for the probability of finding an existing underlying effect (z). The probability of finding a just significant effect is, in the case of p-hacking, inflated with a correction factor containing two parts, firstly a definition

of the population at risk, nonsignificant findings $(1 - ar)$, secondly, the ratio of p-hacking (phr) relative on the already significant values (ar). ar is thereby defined either as the statistical power in case of an underlying effect or as the set significance threshold α (cp. Equations C & E). The procedure for the estimation of just nonsignificant values was exactly the same, except that values were deleted rather than imputed (1 − phr). As both p_{OC_biased} and p_{UC_biased} are conditional on the presence or absence of a true effect, the known unconditional probabilities $P(\text{OC})$ and $P(\text{UC})$ can be recovered by weighting with θ, the assumed *a priori* probability of a hypothesis being true (cp. Equations F & G).

Conditional probabilities in over- and under-caliper both α, and $power$:

$$p_{OC\_unbiased} = \Pr(th < |Z| \leq th + c), \text{where } Z \sim \mathcal{N}(z, 1)$$
$$= \Phi(th + c - z) - \Phi(th - z) + \Phi(-th - z) - \Phi(-th - z - c)$$

Equation B

$$p_{OC\_biased} = p_{OC\_unbiased} \times \left(1 + \frac{(1 - ar) \times phr}{ar}\right)$$

Equation C

$$p_{UC\_unbiased} = \Pr(th - c < |Z| \leq th), \text{where } Z \sim \mathcal{N}(z, 1)$$
$$= \Phi(th - z) - \Phi(th - c - z) + \Phi(-th + c - z) - \Phi(-th - z)$$

Equation D

$$p_{uc} = p_{UC\_unbiased} \times (1 - phr)$$

Equation E

Unconditional probabilities over- and under-caliper for both scenarios, ar: α ($p_{OC|UC_\alpha}$) OR power ($p_{OC|UC_{pow}}$), depending if H0 or H1 is true.

$$P(OC) = (1 - \theta) \times p_{OC_\alpha} + \theta(p_{OC_{pow}})$$

Equation F

$$P(UC) = (1 - \theta) \times p_{UC_\alpha} + \theta(p_{UC_{pow}})$$

Equation G

Up to this step, $P(\text{OC})$ and $P(\text{UC})$ are the probabilities of a test value in the specific caliper, therefore in order to have the same measure obtained by the caliper test, the unconditional

probabilities (Caliper Ratio, CR) of finding a just significant or just nonsignificant effect are calculated relatively to overall probability in both calipers (cp. Equation H).

$$CR = P(OC)/(P(OC) + P(UC))$$

Equation H

In the last step, this equation system has to be solved for phr, the rate of p-hacking that transforms nonsignificant results into significant results. This was done using a unit-root solver.

2. Robustness Checks

2.1. Manual Data Extraction

To test the robustness of the automatically exported results, the author manually coded 20 randomly selected articles. The first step checked whether the export algorithm missed any APA style conform test values or detected them incorrectly. In total, 338 test values were identified in 17 articles, while three articles reported results that did not follow the APA style (bold box in Fig B). Although these results follow APA style, not all are reported correctly, leading to falsely detected test values (outside the dashed box in Fig B). Those can be split into two categories: erroneously reported results (for one test value a comma as a thousand-separator) and ineligible results. Results are ineligible if only the least significant of many results are reported (e.g. minimal $t(11)=2.73$). The reported minimal test values may introduce downward bias and should be excluded ($N=8$, in $K=2$). Nonetheless, the report of all estimates, including the unreported larger test values in the original articles, would be the best solution. Only nine estimates ($N=1$ false export, $N=8$ ineligible exports) or 2.66% of all detected estimates, were false-positive detections.

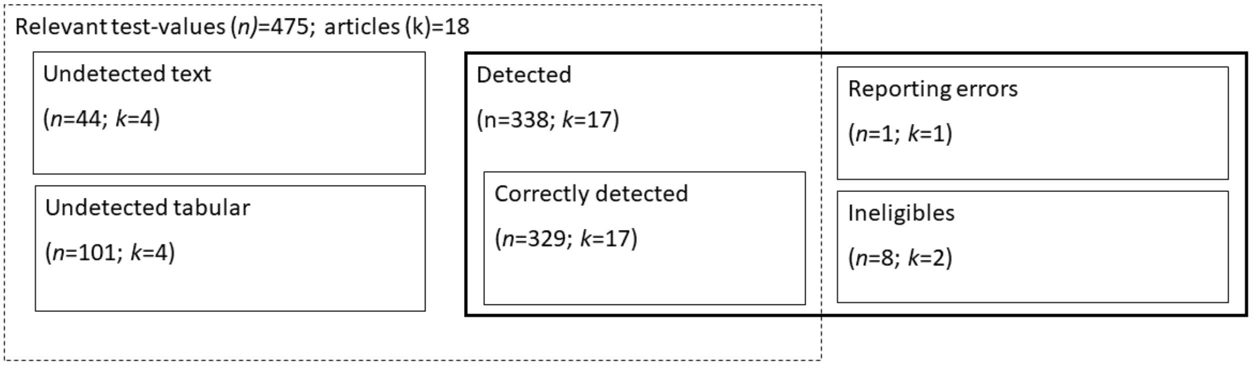

Fig B Truly and falsely detected test values in the automated data extraction process versus manual (human) data extraction

Despite the importance of reducing false-positive detections, detecting as many relevant test values as possible is essential. Only reported test values that follow APA style could be exported, although results reported in a non-APA style format were equally important for the analysis but hard to extract automatically. Those non-APA style compliant results were split up into two categories: results reported in the article's main text but in a non-APA style format and results presented in tables that follow no clear reporting style. It was impossible to detect 44 test values reported in the main text of four articles with the export algorithm (e.g., the report of multiple test values with joint degrees of freedom: *Fs*(1, 29)=4.01 and 4.03). The algorithm exported 88.2% of the in-text test values accurately.

However, the limitation on test values reported in the main text was only a practical solution to allow an automated export procedure, as tabular results follow no clear reporting guidelines. Nonetheless, tabular results are also important, as this article's conclusions should hold to all reported test values in the examined psychological journals. 101 test values from four articles were presented in tables and, therefore, not exportable by the algorithm. The distribution of tabular results was quite skewed, as one single article provided up to 83.25% (84 estimates) of all tabular results. The algorithm could extract 69.41% of all estimates, reported either in the text or in tables. Therefore, the export routine has two advantages: a low FPR of 2.66% and a high detection rate (69.41% for all test values or 88.20% for test values reported in-text only).

Although a high share of correctly detected estimates reduces the risk of bias introduced by a selective test value sample, the differences between the *p*-value distribution of the included and missing test values reported either in the text or tables are subject to a closer look. The *p*-value distribution was favored to the *z*-value distribution because of its bounded nature between 0 and 1, which is not prone to extreme values. However, a direct comparison of the *p*-value distribution of detected relevant values versus falsely non-detected values (tabular or in-text) was not possible because the articles differ in their different research questions that pose different *a priori* probabilities of the hypothesis being true. Therefore, the difference between correctly detected and falsely non-detected test values is only estimable within each original article. There should not be any difference between the detected and not detected test values left holding the study characteristics, such as the *a priori* probability of a hypothesis being true constant. To this end, a fixed-effects linear regression model was estimated that was built upon seven studies with variation respecting detected and not detected test values. There was no significant difference of the mean

p-value between the detected results and the falsely non-detected results reported in-text that were missed by the algorithm *t*(266)=-0.948; *p*=0.344.[3] A slight difference that was also statistically significant at the 10% level occurred in the *p*-value distributions of detected and not detected tabular results *t*(266)=-1.716; *p*=0.087. When looking additionally at the share of significant values, this is primarily of interest for the test on publication selection bias; no differences remained for the in-text *t*(266)=0.338; *p*=0.736 as well as tabular results *t*(266)=1.226; *p*=0.221.

Although the automatic export routine covered nearly all test values reported in the articles' text and a large share of test values, nothing can be said about the relevance of the test values for the primary research outcome. Therefore, as a second robustness check of the data extraction process, a substantial coding of 25 articles was also conducted in the population of articles that report up to five test values. This manual coding extracted only test values directly linked to the primary research outcomes. Robustness and manipulation checks were not extracted in the manual coding but in the automatic procedure. The limitation of the subset of articles with ≤ 5 tests was chosen because only this subset gave hope to be related to primary research outcomes only. In total, only 25% of all primary research outcomes could be extracted automatically.

Furthermore, of the exported values, 49% of all values are not primary research outcomes. Although these robustness analyses show that the automatic extraction procedure is not suited for topic-related meta-analyses, they may be sufficient to recover the standard error for *a priori* meta-analyses. For the analysis of publication selection bias, this poor coverage may not affect the estimator because secondary research outcomes can also be prone to publication selection bias.

2.2. Existing Export Algorithm

The study by Hartgerink, van Aert (12, for the data documentation, see Hartgerink 2016) used a similar approach to export test values from psychological journals following the APA style using the R package *statcheck* (13). Therefore, it is possible to compare the number of exported results of both algorithms to minimize the risk of missing relevant text values. In contrast to the algorithm in this study, *statcheck* also exports *p*-values, which authors should report according to APA style. The *statcheck* algorithm has the advantage that misreported *p*-values that could be identified by a mismatch between the reported *p*-value and test value can also be detected (14). A downside of

[3] Possible dependencies of p-values within an article were not considered because of the low number of included articles. The modelling strategy ignoring these possible dependencies might therefore be more liberal, detecting inexsisting differences.

this approach is, however, the increased error probability in the export, as another structured part has to be extracted in addition to the test value.

Only studies from 1985–2016 that are included in the data of Hartgerink (11) were compared to keep the following comparison as similar as possible. For the algorithm used in the study at hand, it was possible to export 693,713 test values and transform them into their according *p*-values.[4] Therefore, the algorithm used in the study at hand exceeded the export of Hartgerink (11) by around 33%. Besides exporting more test values from the articles, it was possible to include 8% more articles, leading to a more holistic picture. Although the mean *p*-value of the exported results was close (0.096 in the present data vs. 0.098 in the Hartgerink data), differences in the distribution between exported and missed test values may distort the results. Although *statcheck* is valuable for uncovering misreporting, it is too restrictive for the purpose at hand. However, the tool is of great use for the correct (re)calculation of the reported *p*-values.

2.3. Results

2.3.1. Measurement

In the following section, three different robustness checks will be reported: first, a description of the classified distribution of *p*-values (split by deciles of the sample size of the tests, cp. Table B). Second, different specifications of the measurements reported in the main article are shown in detail (cp. Table C).

As shown in the article, the results of the caliper test, as well as the observed *p*-value distribution, provide evidence for publication selection bias. This pattern is also visible when looking at the share of significant test values at the 10%, 5%, 1%, and 0.1% significance thresholds computed separately for each decile of the underlying number of observations (Table B). Over the deciles, the share of statistically significant test statistics drops with a decreasing significance threshold, as expected, whereas around 80% of the test statistics are significant at the 10% significance threshold. The difference between the deciles increased the smaller the significance threshold was set. Deciles with fewer observations show substantially lower shares of significant values than larger ones. In the most extreme case, the 0.1% significance threshold, in the smallest decile, 10.9%, and in the largest 35.8% of the test values were statistically significant. This result shows a larger share of just significant results in studies with a lower number of observations that are more prone to publication selection bias practices (for a similar logic, see p-curve & p-uniform

---

[4] To ensure comparability, reported *z*-values were also included in the comparison of *p*-values.

15). For the 5%- and 10% CR a increasing imbalance of just significant and nonsignificant results is observed that may, contrary to the expectations, point to the fact that larger studies are more affected by publication selection bias.

**Decile intervals**	**% results** *p* **< 0.1**	**% results** *p* **< 0.05**	**% results** *p* **< 0.01**	**% results** *p* **< 0.001**	**5%-CR**	**10%-CR**	**Number of observations**
[2, 14]	0.765	0.678	0.419	0.109	0.596	0.609	49,362
(14 21]	0.789	0.712	0.482	0.191	0.598	0.614	52,853
(21, 29]	0.790	0.714	0.480	0.210	0.601	0.625	48,346
(29, 38]	0.797	0.720	0.484	0.224	0.620	0.641	47,327
(38, 48]	0.803	0.729	0.495	0.239	0.626	0.639	48,846
(48, 63]	0.809	0.731	0.487	0.234	0.608	0.635	49,367
(63, 84]	0.814	0.737	0.497	0.246	0.612	0.636	46,379
(84, 119]	0.815	0.745	0.512	0.268	0.619	0.642	48,377
(119, 211]	0.824	0.757	0.533	0.286	0.625	0.649	48,460
(211, 271 million]	0.831	0.778	0.595	0.358	0.630	0.652	48,679

Table B Share of statistically significant results at different thresholds (10%, 5%, 1% & 0.1%) at deciles of the numbers of observations for each test statistics

	P(sig.) 1%-CR	P(sig.) 5%-CR	P(sig.) 10%-CR	Stat. power small es	Stat. power medium es	Stat. power large es	Nr. of studies, *K*	Nr. of test values, *N*
Main analysis (*d*)	0.565	0.613	0.633	0.233	0.593	0.804	35,515	487,996
Articles reporting only low nr. of test values								
$N \leq 5$	0.578	0.646	0.664	0.335	0.690	0.855	12,114	33,385
Effect size metrics								
r/ biserial	0.565	0.613	0.633	0.160	0.570	0.832	35,486	487,425
Article format								
PDF	0.554	0.618	0.634	0.237	0.600	0.809	1,735	19,550
HTML	0.566	0.613	0.633	0.233	0.592	0.804	33,780	468,446
Test statistic								
χ^2 if df = 1	0.615	0.638	0.653	0.305	0.697	0.884	5,417	19,388
F if df1 = 1	0.575	0.620	0.645	0.150	0.464	0.717	25,759	281,137
r	0.453	0.586	0.589	0.168	0.520	0.774	4,142	22,831
t	0.554	0.599	0.613	0.375	0.810	0.948	20,798	164,640

Note Thresholds for small, medium and large effects, d: 0.2, 0.5, 0.5, r: 0.1, 0.3., 0.5

Table C Publication selection bias and statistical power for different effect size measures and sub-samples

2.3.2. Parametric models of time trend

Besides the graphical models shown in the main article, both a categorical specification and a linear time trend were estimated to allow more flexible modeling of the time trend. The categorical specification divides the data into five categories, each containing ten years. The only exception is the reference category (2015–2017), which covers only the three most recent years. The study year was set to zero for 2017 to ease the interpretation of the regression intercept across the two specifications. Therefore, the intercept can be interpreted as the current state of the statistical power, publication selection bias, or FDR, respectively.

The results in Table D (categorical) and Table E (linear) for both the 5%- and 10%-CT and 1%-CT show that between 5 and 12% of the nonsignificant results were turned significant by p-hacking. In the alternative publication bias scenario, 25 to 39% were not published. All CTs produced similar results, while the larger 10%-CT showed slightly stronger evidence on publication bias and p-hacking, while the 1%-CT showed a weaker level of bias. In contrast to the results on the statistical power, the results on publication selection bias show no time trend on neither the categorical nor the linear specification. All in all, there is robust evidence for a mostly time-invariant substantial publication selection bias rate in the psychological literature without any signs of decline.

	1%-CR		5%-CR		10%-CR	
	p-hacking	publication bias	p-hacking	publication bias	p-hacking	publication bias
	(1)	(2)	(3)			
Constant	0.048***	0.299***	0.083***	0.357***	0.126***	0.398***
(ref. 2015–2017)	(0.010)	(0.068)	(0.006)	(0.025)	(0.007)	(0.020)
1975–1984	-0.027	-0.183	0.004	0.049	0.006	0.048
	(0.023)	(0.150)	(0.015)	(0.058)	(0.014)	(0.044)
1985–1994	-0.015	-0.086	-0.002	0.016	0.001	0.028
	(0.012)	(0.083)	(0.008)	(0.031)	(0.008)	(0.024)
1995–2004	-0.012	-0.054	-0.007	0.001	-0.004	0.012
	(0.012)	(0.077)	(0.007)	(0.029)	(0.007)	(0.023)
2005–2014	-0.019	-0.100	-0.0005	0.015	0.005	0.031
	(0.011)	(0.076)	(0.007)	(0.028)	(0.007)	(0.022)
N	35	35	36	36	36	36
R^2	0.103	0.086	0.065	0.045	0.098	0.096

Note: $^{*}p<0.05$ $^{**}p<0.01$ $^{***}p<0.001$; standard errors in parentheses, p-hacking under the assumption of θ = 0.5 & a priori statistical power for d=0.5. Differences in *N* by different CRs are caused by the exclusion of years with too many articles (<20) or test values (<20) in the respective CR.

Table D Publication selection bias on categorical study year

	1%-CR		5%-CR		10%-CR	
	p-hacking	publication bias	p-hacking	publication bias	p-hacking	publication bias
	(1)	(2)	(3)			
Constant	0.038***	0.257***	0.083***	0.367***	0.129***	0.418***
(ref. 2017)	(0.006)	(0.037)	(0.004)	(0.014)	(0.004)	(0.011)
+ 10 Years	0.038***	0.023	0.002	0.0001	0.002	-0.001
	(0.006)	(0.022)	(0.002)	(0.008)	(0.002)	(0.007)
N	35	35	36	36	36	36
R^2	0.028	0.033	0.026	0.00000	0.021	0.001

Note: $^{*}p<0.05$ $^{**}p<0.01$ $^{***}p<0.001$; standard errors in parentheses, p-hacking under the assumption of θ = 0.5 & a priori statistical power for d=0.5

Table E Publication selection bias on linear study year

Publication selection bias varied substantially across different psychological subfields (Fig C). The considerable variation till 1985 (with the exception of cognitive psychology) is mostly due to the small number of observations. From 1985 to 2010, the publication bias and p-hacking rate were

quite stable. One exception is the increasing p-hacking and publication bias rate in social psychology over time.

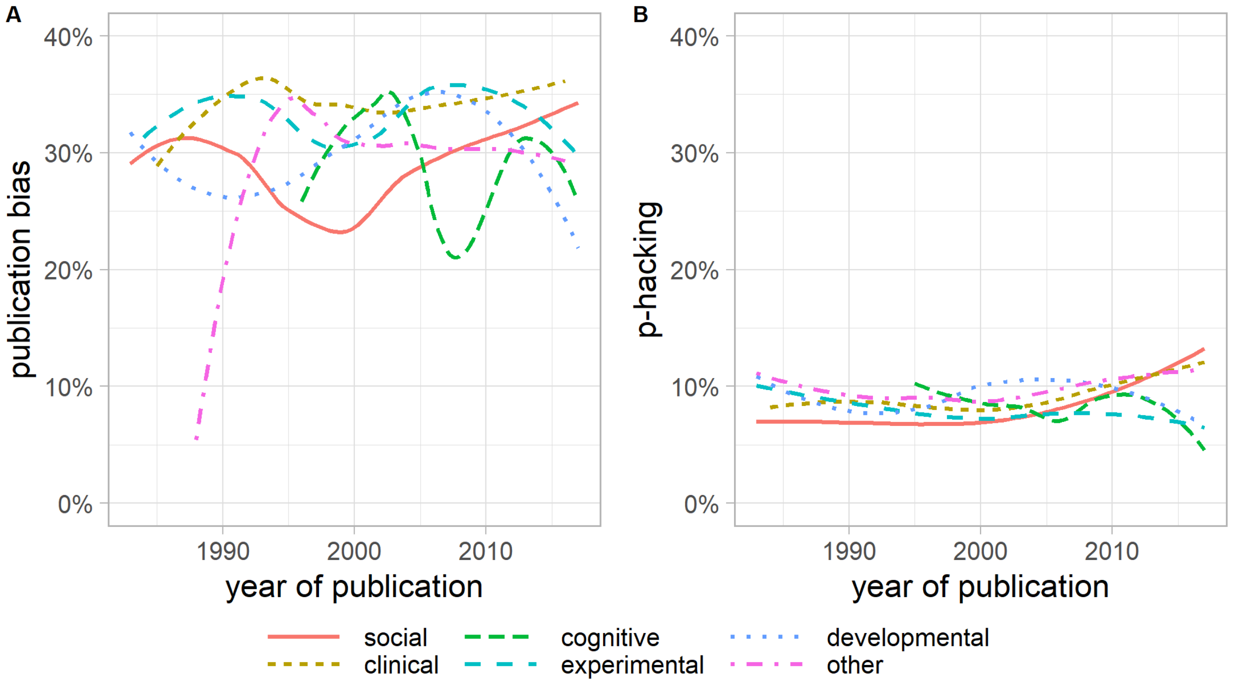

Fig C Publication selection bias by psychological subfield from nonparametric LOESS regressions

The parametric regression models in both the categorical (Table F) and the linear specification (Table G) confirmed the graphical findings from the main article that suggested an increased statistical power over time, especially in recent years. However, the overall level of the statistical power for an underlying medium true effect (d = 0.5) was at around 67% in recent years. All in all, the statistical power across all models was far too low compared to the benchmark of 80%, as proposed by Cohen (3: 56) except for large underlying effects.

	Statistical power assuming an *a priori* effect size		
	d=0.2	d=0.5	d=0.8
	(1)	(2)	(3)
Constant	0.294***	0.672***	0.855***
(ref. 2015–2017)	(0.007)	(0.011)	(0.009)
1975–1984	-0.113***	-0.143***	-0.088***
	(0.015)	(0.024)	(0.018)
1985–1994	-0.093***	-0.113***	-0.064***
	(0.009)	(0.014)	(0.011)
1995–2004	-0.082***	-0.113***	-0.077***
	(0.008)	(0.013)	(0.010)
2005–2014	-0.047***	-0.059***	-0.038***
	(0.008)	(0.013)	(0.010)
N	43	43	43
R^2	0.813	0.745	0.670

Note: $^{*}p$<0.05 $^{**}p$<0.01$^{***}p$<0.001; standard errors in parentheses

Table F Statistical power and significant effects on categorical study year

	Statistical power assuming an *a priori* effect size		
	d=0.2	d=0.5	d=0.8
	(1)	(2)	(3)
Constant	0.274***	0.645***	0.835***
(ref. 2017)	(0.004)	(0.007)	(0.006)
+ 10 Years	0.031***	0.039***	0.022***
	(0.002)	(0.004)	(0.003)
N	43	43	43
R^2	0.800	0.697	0.518

Note: $^{*}p$<0.05 $^{**}p$<0.01$^{***}p$<0.001; standard errors in parentheses

Table G Statistical power and significant effects on linear study year

Because the statistical power, unlike publication selection bias, can also be computed on the level of test values instead of aggregating on the yearly level. As shown in Tables H and I, this does not affect the results.

	Statistical power assuming an *a priori* effect size		
	d=0.2	*d*=0.5	*d*=0.8
	(1)	(2)	(3)
Constant	0.295***	0.672***	0.855***
(ref. 2015–2017)	(0.005)	(0.006)	(0.004)
1975–1984	-0.115***	-0.147***	-0.091***
	(0.008)	(0.012)	(0.011)
1985–1994	-0.093***	-0.113***	-0.065***
	(0.006)	(0.008)	(0.006)
1995–2004	-0.083***	-0.113***	-0.077***
	(0.006)	(0.007)	(0.005)
2005–2014	-0.047***	-0.060***	-0.038***
	(0.006)	(0.007)	(0.005)
N	487,996	487,996	487,996
R^2	0.017	0.016	0.011

Note: $^{*}p<0.05$ $^{**}p<0.01$ $^{***}p<0.001$; cluster robust standard errors on the article level in parentheses

Table H Statistical power on test value level on categorical study year

	Statistical power assuming an *a priori* effect size		
	d=0.2	*d*=0.5	*d*=0.8
	(1)	(2)	(3)
Constant	0.276***	0.648***	0.838***
(ref. 2017)	(0.003)	(0.004)	(0.003)
+ 10 Years	0.032***	0.042***	0.025***
	(0.001)	(0.002)	(0.002)
N	487,996	487,996	487,996
R^2	0.017	0.015	0.009

Note: $^{*}p<0.05$ $^{**}p<0.01$ $^{***}p<0.001$; cluster robust standard errors on the article level in parentheses

Table I Statistical power on test value level on categorical study year

The estimated statistical power shows slight differences between the psychological subdisciplines (Fig D). Note that the assumption of a medium underlying true effect may not hold over the

subfields, the time trend, as long as no change in the underlying true effect over time is comparable. All subfields show an increasing statistical power over the years. [5]

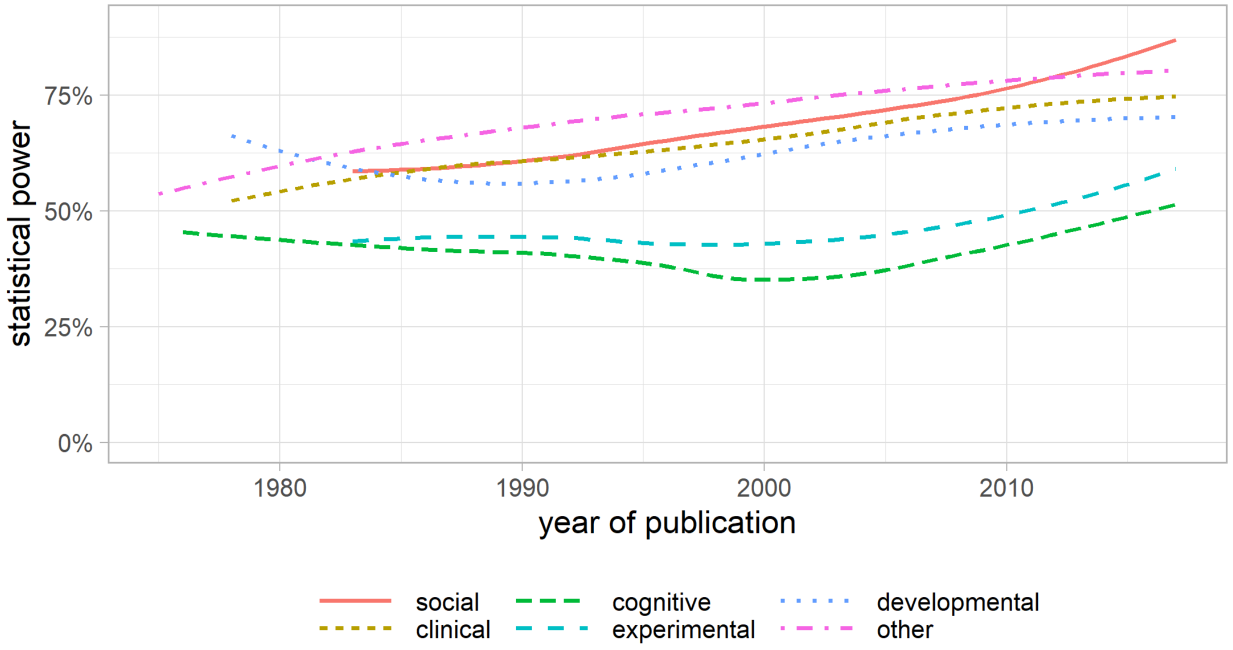

Fig D Statistical power by psychological subfield for medium true effect (*d* = 0.5) from nonparametric LOESS regressions

[5] These trends were also statistically significant in linear as well as categorical models.

As the FDR is only a composite estimate of both the statistical power and publication selection bias, no separate parametric models are shown. The subfield-specific FDR (Fig E) mirrors the trend of statistical power and the publication selection bias rate. The overall FDR seems strikingly constant in all subfields. However, cognitive psychology has, because of its very low statistical power, a particularly large FDR (uninflated or with publication bias).

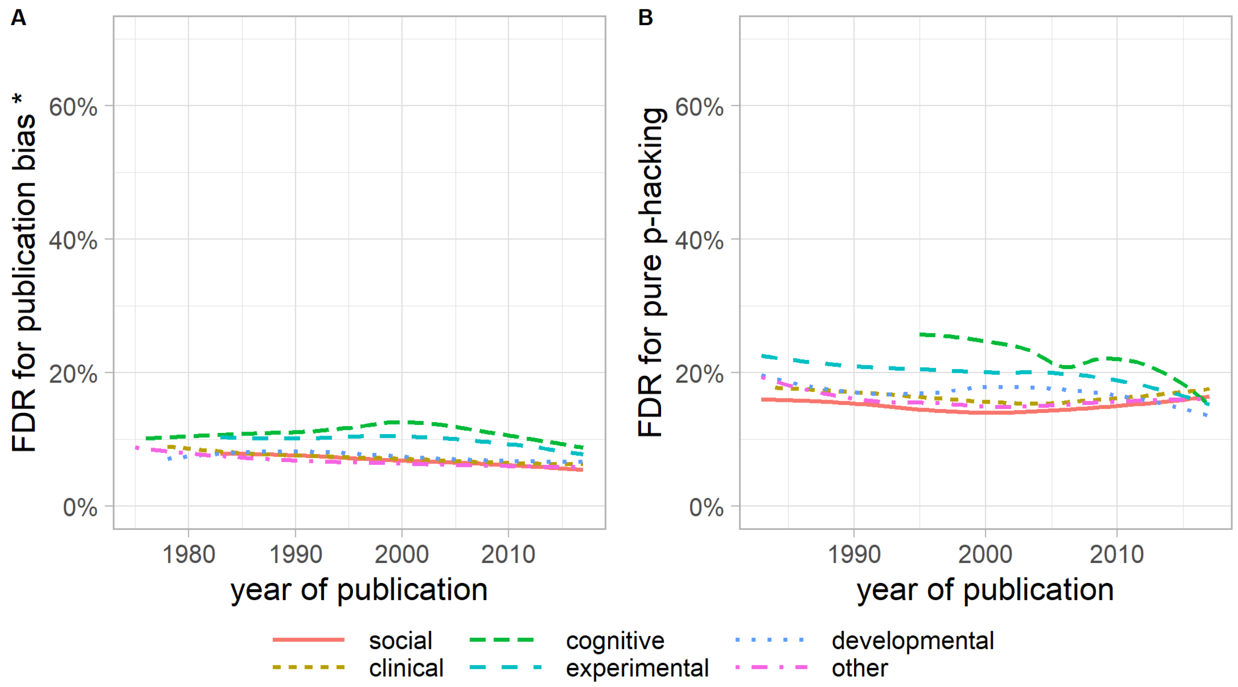

*Note: * The FDR for publication bias is equivalent to FDR assuming no publication selection bias.*

Fig E FDR by psychological subfield from nonparametric LOESS regressions

2.4. Central Limitation

The central limitation of the article is the missing fit between the observed and expected share of statistically significant results. In addition to the scenarios presented in Table 2 in the main text, the scenarios are also presented graphically in Fig F. The intercept of the horizontal line (at 73.0%) shows the situations where the possible scenarios match the observed share of significant results at the 5% significance threshold. The most speculative parameter, the share of true hypotheses, is plotted on the x-axis. Scenarios exist for a different statistical power as well as different p-hacking rates.

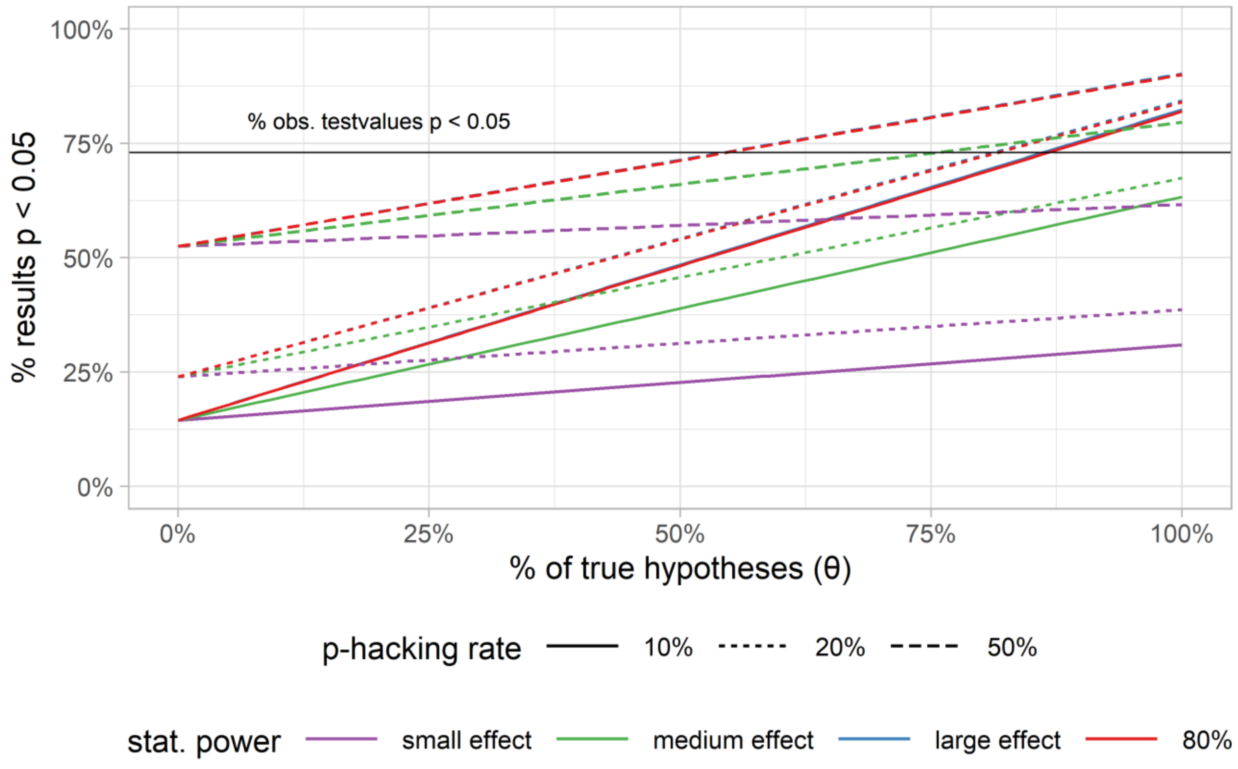

Fig F Scenarios to fit the empirically observed share of statistically significant results P(p < 0.05) ~ 73.0%

References

1. Borenstein M. Introduction to Meta-Analysis. Repr. ed. Chichester: Wiley; 2011. 421 p.
2. Harris DJ, Kolen MJ. Bootstrap and Traditional Standard Errors of the Point-Biserial. Educational and Psychological Measurement. 1988;48(1):43-51.
3. Cohen J. Statistical Power Analysis for the Behavioral Sciences. 2nd ed. Hillsdale, N.J.: L. Erlbaum Associates; 1988. 567 p.
4. Doucouliagos H, Stanley TD. Publication Selection Bias in Minimum-Wage Research? A Meta-Regression Analysis. British Journal of Industrial Relations. 2009;47(2):406-28.
5. Gerber AS, Malhotra N. Publication Bias in Empirical Sociological Research. Sociological Methods & Research. 2008;37(1):3-30.
6. Gerber AS, Malhotra N. Do Statistical Reporting Standards Affect What Is Published? Publication Bias in Two Leading Political Science Journals. Q J Polit Sci. 2008;3(3):313-26.
7. Schneck A. Examining Publication Bias – A Simulation-Based Evaluation of Statistical Tests on Publication Bias. PeerJ. 2017;5:e4115.
8. Higgins JPT, Thompson SG. Quantifying Heterogeneity in a Meta-Analysis. Statistics in medicine. 2002;21(11):1539-58.
9. Lee DS, Lemieux T. Regression Discontinuity Designs in Economics. J Econ Lit. 2010;48(2):281-355.
10. Lakens D. What p-hacking Really Looks Like: A Comment on Masicampo and LaLande (2012). The Quarterly Journal of Experimental Psychology. 2015;68(4):829-32.
11. Hartgerink HC. 688,112 Statistical Results: Content Mining Psychology Articles for Statistical Test Results. Data. 2016;1(3).
12. Hartgerink CHJ, van Aert RCM, Nuijten MB, Wicherts JM, van Assen MALM. Distributions of p-Values Smaller Than .05 in Psychology: What Is Going On? PeerJ. 2016;4:e1935.
13. Epskamp S, Nuijten MB. statcheck: Extract Statistics from Articles and Recompute p Values (Version 1.3.0). https://cran.r-project.org/web/packages/statcheck/index.html2018.
14. Nuijten MB, Hartgerink CHJ, van Assen MALM, Epskamp S, Wicherts JM. The Prevalence of Statistical Reporting Errors in Psychology (1985–2013). Behavior Research Methods. 2016;48(4):1205-26.
15. van Aert RCM, Wicherts JM, van Assen MALM. Conducting Meta-Analyses Based on p Values. Perspectives on Psychological Science. 2016;11(5):713-29.
