# Supplementary material for: Are most published research findings false? Trends in statistical power, publication selection bias, and the false discovery rate in psychology (1975–2017)
Source: PLoS One. 2023 Oct 17;18(10):e0292717. doi: 10.1371/journal.pone.0292717 (PMC10581498; doi:10.1371/journal.pone.0292717)
Supplement: Attachment — Submitted filename: review-are-most-published-research-findings-false_em.pdf [file pone.0292717.s003.pdf]

This manuscript analyzes 650,000 test statistics from 40,000 published psychology articles over almost 40 years to provide an estimation of statistical power, publication bias and p-hacking, and the false discovery rate across the field. To briefly summarize the results, the statistical power was low, but shows a (small) increase over time. There is an excess of statistically significant results, and the false discovery rate across the field is around 32%.

Overall, I think this article does a good job of conveying the issue of an excess of statistically significant results in the psychology field. It is particularly worrisome that so many reported results are statistically significant, and that the statistical power has increased so little over the past few years. The introduction of the article is accessible, gives a clear introduction to topics like publication bias, and provides a comprehensive overview of how these issues relate to problematic consequences within science. I checked out the supplemental materials associated with this manuscript. I did not try to reproduce all results, but it is relatively easy to load in data and to extract specific information from the script. I also appreciate the use of a README file to indicate which files direct me to which results.

This manuscript can benefit from more clarification on certain points to better convey its message. In short, I think the "p-hacking" element of the study deserves more nuance, some robustness checks and other methodological choices could be added, and I believe a redistribution of the information conveyed in the manuscript and supplemental material is warranted. Improving these points can help better facilitate the use of the considerable amount of results, and help readers better assess the methods and results in the study.

I have constructed the remainder of this review into four sections: p-hacking, other methodology, supplemental material, and minor remarks. The numbers at the beginning of each point refer to line numbers in the manuscript. Please feel free to contact me for clarifications.

Signed,

Esther Maassen

P-hacking

- I think it is important to distinguish more between publication bias and p-hacking in your study. If I understand correctly, publication bias and p-hacking are measured together as one, by performing the caliper test on two bins of p-values just around the significance level of .05. If you, based on the caliper test, find a bump just below $p = .05$, this could be an

indication of p-hacking. If you find that significant results are more likely to be found than non-significant ones, but there is no bump just below .05, this could be an indication of publication bias. Of course, if you find publication bias, there could ALSO be p-hacking going on, but I do not think this can be concluded from the data you have with the method you use. Your results (line 171) seem to indicate that there is not necessarily a bump below .05. As such, I think your data show evidence for publication bias, but not necessarily p-hacking, and this distinction (as well as that they are measured together as one entity) should be made clear in the rest of the manuscript. The way it is stated now, in both text as title, it seems there is both publication bias AND p-hacking present in the data, as if they were measured as separate entities.

- 39: "p-hacking as forms of scientific misconduct" indicates to me that you will attempt to detect forms of p-hacking in the data that can be classified as scientific misconduct. I think, however, that the method you use to detect p-hacking in your study is not able to detect scientific misconduct as it is generally understood (i.e., falsification or fabrication of data).

- 125: You state "therefore, a strong non-significant result is equally likely to be treated by publication bias and/or p-hacking to produce a statistically significant result than a nearly significant result". I think this might be a reasonable assumption for publication bias, but not for p-hacking, which could be elaborated on.

Other methodology

- 91: I am not sure it is ideal to transform all test values to Cohen's d (see this article: 10.1002/jrsm.1218). I think it is reasonable to assume, since you have so many results and cannot check manually, that the r test statistics in your sample are product-moment correlations, which are based on two underlying continuous variables that are assumed to follow a bivariate normal distribution. However, Cohen's d is computed based on one continuous variable and one dichotomized one (the grouping variable), meaning there is artificial dichotomization if you convert r to d. The linked article suggests transforming χ^2 values to tetrachoric correlations, F and t values to biserial correlations (NOT point-biserial correlations), and then including them with the product-moment correlations from the r values in meta-analysis (or transform them to Fisher's r-to-z if you want to solve the unbounded issue you reference in the supplemental material). This approach might be

more appropriate, even though "pooling such heterogeneous effects together in meta-analyses is only applicable if the underlying effect is of no substantial interest", as you state on line 93.

- If I understand correctly, publication bias and p-hacking are measured together at once, by performing the caliper test on two bins of p-values just around the significance level of .05. From the manuscript, it is not immediately clear to me what the bin width is that is used. You reference Head et al. (2015), who claim there is evidence for widespread p-hacking, but this article (10.7717/peerj.3068) finds that these results are not robust and are dependent on the bin width. Would it be possible to do a robustness check based on bin widths (value s?) in your study?

- As in the previous point, I would be interested in the results of using a smaller prior probability of hypotheses being true (< 10%).

Supplemental Material

The supplemental material contains quite some information that I think is essential to include in the manuscript, which is relatively sparse in the information it contains. I had to use the supplemental material early on when I was reading the manuscript. This is not necessarily a bad thing, and the information in the supplemental material is very clear (for example, the explanation on test value extraction from text). However, I found much important information in the supplemental material that is essential for understanding the process behind this body of research, and should be included in the manuscript (for example, operationalization of the simulation study). The following bullet points describe some information I found that I think would benefit from inclusion in the manuscript, but the list is not exhaustive.

- 81: the text states that the dataset contains 39,324 articles and 649,959 test values. When looking at dataset D1 (data.rda), I count 54,170 rows. When I remove all rows that do not contain any values in columns tv_0 to tv_209, I still have 42,313 entries left. Similarly: you report 649,959 test values, but I count 739,035 in the data. These estimates (54,170 and 739,035) are similar to the export estimates reported in S1 of the supporting material, but are not the same. Generally speaking, I think it would be helpful to elaborate on what kind of test values are included in the manuscript text, as well as a bit more information on the

dropout in the supporting materials (what happens to the 80k test values that cannot be computed from z-value to Cohen's d?). Personally, I would also be interested in some descriptives in the manuscript on which test statistics are reported most often, or the median number of test statistics per article etc.

- 106. The information in the supplemental material on page 14 states: "It is important to note that, with the estimation strategy, the statistical power calculations are based on an even larger estimate than present in reality." I think this point should be mentioned in the manuscript.

- Supplemental Material, page 4: you state a limitation regarding the test values: "However, it was not possible to distinguish if a test that allowed for two-sided testing was used only one-sided (t, r). In the final sample, this concerns 27,9% of all test values.". I would like to see this in a small section of the manuscript (maybe limitations?) instead of in the supporting materials.

- Supplemental Material, page 14: "Therefore, the estimated statistical power tends to be overestimated, which seems essential to avoid results that produce an overdramatization that is only caused by the used estimation strategy." Could you elaborate on this point?

- Supplemental Material, page 16/17: the first paragraph of the method section includes essential material that should be in the manuscript, especially the part of the reduction to 43 observations. Additionally, would it be possible to estimate the total distribution of p-values at once, without subsetting in subfields? Would you want to, is this interesting information?

- Supplemental Material, page 18: I think the argument that not all test statistics can be found automatically, and neither can tabular results, is worthwhile and could be included in the manuscript with some elaboration. Is this a limitation of the study?

- Supplemental Material page 21: the “Publication bias and p-hacking are larger” result could be included in the manuscript.

- Throughout the Supplemental Material, some limitations to the study are mentioned. I think a limitation section, however short, is essential to include in the conclusion and discussion section.

Minor remarks

- 52: You state "since the pioneering study by Cohen (12: 150f.) there has been no increase in statistical power." Is this across all psychology fields?

- 55: I would like to see an elaboration of the difference between the FPR (False Positives / (False Positives + True Negatives)) and FDR (False Positives / (False Positives + True Positives)) here. I think the "The FDR estimates this" text threw me off when I first read it. I think it would benefit readers to also display Table S5 (Truth Table) here.

- 59: from which field is the FDR of 14%? Does reference 16 (Ioannidis et al.) offer a better estimate of the FDR?

- 67: I think your explanation of publication bias is very clear. However, some elaboration on p-hacking here might be interesting, since there are many different forms of p-hacking. How do these forms relate to your research?

- 138: It is unclear to me what the sentence starting with "Furthermore" means.

- 153: Later on in the text (around line 220) you include a short discussion on the difference or similarities in results across subfields of psychology. I think this would be appropriate here as well, and the same goes for results around line 173. I also like Figure S7 in the Supplemental Material and would like to see it included in the manuscript.

- 159: What does it mean that the replication rate is nearly identical to the statistical power?

- 160: What does it mean that the time trend correlation is so large? Is this a problematic result?

- 172: I would like to see more explanation on how the 22.8% is calculated (which binwidth? does it matter?), and what it means. If I read correctly, it is the percentage of excessive significant results, assuming there is a uniform distribution?

- 173: I would not use the term "became significant". Also on line 192.

- Figure 1, panel b: I do not immediately understand what the y-axis stands for. Percentage of publication bias in a field? Maybe FDR?

- Table S7: it looks like column 4 to 7 display statistical power, because it is stated in the row above, but I think these estimates should be FDR?
