# Supplementary material for: Are most published research findings false? Trends in statistical power, publication selection bias, and the false discovery rate in psychology (1975–2017)
Source: PLoS One. 2023 Oct 17;18(10):e0292717. doi: 10.1371/journal.pone.0292717 (PMC10581498; doi:10.1371/journal.pone.0292717)
Supplement: Attachment — Submitted filename: response.docx [file pone.0292717.s005.docx]

Dear Esther Maassen, dear Dr. André Gillibert,

Many thanks for your constructive and very helpful comments that improved the paper substantially. Besides the changes reported below, the manuscript was, as suggested, entirely rewritten to assure better readability and at the same time convey more methodological details in the main text.

Below you can find my point by point replies to each of your remarks. The reply text is highlighted in red. Passages that were added in the manuscript and addressed the related issue were highlighted in green.

I hope that the manuscript is now suitable for publication in PLOS ONE.

Again many thanks for your remarks and best wishes

Andreas Schneck

**Content**

[Reviewer 1	1](#_Toc83593518)

[Reviewer 2	8](#_Toc83593519)

# Reviewer 1 – Esther Maassen

[shortened]

**P-hacking**

- I think it is important to distinguish more between publication bias and p-hacking in your study. If I understand correctly, publication bias and p-hacking are measured together as one, by performing the caliper test on two bins of p-values just around the significance level of .05. If you, based on the caliper test, find a bump just below p = .05, this could be an indication of p-hacking. If you find that significant results are more likely to be found than non-significant ones, but there is no bump just below .05, this could be an indication of publication bias. Of course, if you find publication bias, there could ALSO be p-hacking going on, but I do not think this can be concluded from the data you have with the method you use. Your results (line 171) seem to indicate that there is not necessarily a bump below .05. As such, I think your data show evidence for publication bias, but not necessarily p-hacking, and this distinction (as well as that they are measured together as one entity) should be made clear in the rest of the manuscript. The way it is stated now, in both text as title, it seems there is both publication bias AND p-hacking present in the data, as if they were measured as separate entities.

Thank you for your remark, I clarified on p-hacking and publication bias in the manuscript. Indeed it is not possible to separate both analytically, especially using the caliper test (a test originally designed to detect publication bias alone). In the results, the 22.8% publication bias results from a medium-sized bump with 61.4 just significant testvalues in the caliper than the expected 50%.

In order to avoid terminological inaccuracies, I changed to the term publication selection bias, that serves as an umbrella term for both possible reasons, publication bias and/or p-hacking.

I furthermore clarified in the manuscript:

“Publication selection bias is well-documented in various scientific disciplines (see, e.g., 9, 28) and may occur in two forms: publication bias and p-hacking. Under publication bias, research results are not published at all due to their research outcome (e.g., being non-significant). Publication bias, however, only includes searching for significant results in new datasets, while non-significant results remain in the file drawer (29). P-hacking, in contrast, searches for significant results within one dataset (22, 30). Practices of p-hacking include thereby excluding outliers, switching outcomes, measures, or covariates to obtain a significant result (22); p-hacking is therefore very similar to the concept of selective reporting bias. “

- 39: "p-hacking as forms of scientific misconduct" indicates to me that you will attempt to detect forms of p-hacking in the data that can be classified as scientific misconduct. I think, however, that the method you use to detect p-hacking in your study is not able to detect scientific misconduct as it is generally understood (i.e., falsification or fabrication of data).

I completely agree, this was not clear, the wording is now changed to:

“The article at hand shows the development of statistical power and publication selection bias in articles published in psychology between 1975 and 2017”

- 125: You state "therefore, a strong non-significant result is equally likely to be treated by publication bias and/or p-hacking to produce a statistically significant result than a nearly significant result". I think this might be a reasonable assumption for publication bias, but not for p-hacking, which could be elaborated on.

I completely agree in the case of publication bias. But also under p-hacking results can be changed switching control variables (e.g. controlling on so-called collider variables). In the conditions of the test oin publication selection bias are therefore clarified:

“For the prevalence estimate to be valid for all test values, two assumptions have to be made. First, publication selection bias is implemented irrespective of the p-value achieved at first hand, meaning that a strongly non-significant result is equally likely to be treated by publication selection bias to produce a significant result. This process is also applicable under p-hacking because results can be changed by switching control variables (e.g. controlling on so-called collider variables, 54). Second, the success of publication bias is not guaranteed and depends on the underlying data and the number of trials to achieve a significant result by the researcher. Overall, the CT was a conservative estimate of publication selection bias in a simulation study (see Section 1.1., Supporting Information)..”

**Other methodology**

- 91: I am not sure it is ideal to transform all test values to Cohen's d (see this article:

10.1002/jrsm.1218). I think it is reasonable to assume, since you have so many results and cannot check manually, that the r test statistics in your sample are product-moment correlations, which are based on two underlying continuous variables that are assumed to follow a bivariate normal distribution. However, Cohen's d is computed based on one continuous variable and one dichotomized one (the grouping variable), meaning there is artificial dichotomization if you convert r to d. The linked article suggests transforming χ² values to tetrachoric correlations, F and t values to biserial correlations (NOT point-biserial correlations), and then including them with the product-moment correlations from the r values in meta-analysis (or transform them to Fisher's r-to-z if you want to solve the unbounded issue you reference in the supplemental material). This approach might be more appropriate, even though "pooling such heterogeneous effects together in metaanalyses is only applicable if the underlying effect is of no substantial interest", as you state on line 93.

Many thanks for the remark, I completely agree that biserial-correlation would be favorable. In this specific case however it was only possible to calculate the biserial correllation for t, r, and F-tests with df1=1. Cohen’s d was the effect sizes that alloed to include as much test values as possible.

I included the results for point-biserial and biserial correlations in the robustness section.

- If I understand correctly, publication bias and p-hacking are measured together at once, by performing the caliper test on two bins of p-values just around the significance level of .05.

From the manuscript, it is not immediately clear to me what the bin width is that is used. You reference Head et al. (2015), who claim there is evidence for widespread p-hacking, but this article (10.7717/peerj.3068) finds that these results are not robust and are dependent on the bin width. Would it be possible to do a robustness check based on bin widths (value s?) in your study?

I completely agree, the results in the firs submitted manuscript were based on the 5%-Caliper. In order to assess the robustness of the results, I also calculated the 10% and the 1% caliper, the results are now included also graphically in the main text in order to give a transparent parameter choice. The pattern was however similar compared to the Head study resulting in a smaller bias estimate in narrower calipers.

- As in the previous point, I would be interested in the results of using a smaller prior probability of hypotheses being true (< 10%).

Now, I included also results for 1% true hypotheses.

**Supplemental Material**

The supplemental material contains quite some information that I think is essential to include in the manuscript, which is relatively sparse in the information it contains. I had to use the supplemental material early on when I was reading the manuscript. This is not necessarily a bad thing, and the information in the supplemental material is very clear (for example, the explanation on test value extraction from text). However, I found much important information in the supplemental material that is essential for understanding the process behind this body of research, and should be included in the manuscript (for example, operationalization of the simulation study). The following bullet points describe some information I found that I think would benefit from inclusion in the manuscript, but the list is not exhaustive.

Thank you for your suggestion of shifting more information from the supplemental material to the manuscript, I now included a more detailed description of the used method, the data, as well as robustness results in the manuscript.

- 81: the text states that the dataset contains 39,324 articles and 649,959 test values. When looking at dataset D1 (data.rda), I count 54,170 rows. When I remove all rows that do not contain any values in columns tv_0 to tv_209, I still have 42,313 entries left. Similarly: you report 649,959 test values, but I count 739,035 in the data. These estimates (54,170 and 739,035) are similar to the export estimates reported in S1 of the supporting material, but are not the same. Generally speaking, I think it would be helpful to elaborate on what kind of test values are included in the manuscript text, as well as a bit more information on the dropout in the supporting materials (what happens to the 80k test values that cannot be computed from z-value to Cohen's d?). Personally, I would also be interested in some descriptives in the manuscript on which test statistics are reported most often, or the median number of test statistics per article etc.

I completely agree, this was not entirely clear from the manuscript. The data cleaning procedure is now laid out more clearly in a consort-styled flowchart (Fig 2). Every dropout from the data is now noted there.

- 106. The information in the supplemental material on page 14 states: "It is important to note that, with the estimation strategy, the statistical power calculations are based on an even larger estimate than present in reality." I think this point should be mentioned in the manuscript.

Included in the manuscript:

On the level of meta-analyses, the average meta-analytic effect on each subdomain is about 0.499 (median: 0.479). However, the subfields show considerable variation in effects from very small (*d* = 0.002) to very large effect sizes (*d* = 1.206). According to the benchmark of Cohen (49) the average effect can therefore be classified as medium. Therefore, the obtained estimate is rather optimistic compared to literature that found rather small effect sizes to be common (60). This slightly larger effect in this study could result in an overly optimistic assessment of the statistical power.

- Supplemental Material, page 4: you state a limitation regarding the test values: "However, it was not possible to distinguish if a test that allowed for two-sided testing was used only one-sided (t, r). In the final sample, this concerns 27,9% of all test values.". I would like to see this in a small section of the manuscript (maybe limitations?) instead of in the supporting materials.

I completely agree, the limitations are now included, as Reviewer 2 suggested, in the discussion section.

- Supplemental Material, page 14: "Therefore, the estimated statistical power tends to be overestimated, which seems essential to avoid results that produce an overdramatization that is only caused by the used estimation strategy." Could you elaborate on this point?

I clarified in the manuscript:

However, this hybrid estimator still tends to overestimate the average effect, which results in too high or optimistic retrospective power estimates. For retrospective power analyses, this is deliberately done to avoid any overdramatization of a seemingly low statistical power (cp. 39)

- Supplemental Material, page 16/17: the first paragraph of the method section includes essential material that should be in the manuscript, especially the part of the reduction to 43 observations. Additionally, would it be possible to estimate the total distribution of pvalues at once, without subsetting in subfields? Would you want to, is this interesting information?

Thank you for your suggestion. Because the p-value distribution is not in the center of interest I rather did robustness analyses on the statistical power estimates on test value level. The information is now also included in the manuscript.

All three measures, statistical power, publication selection bias, and the false discovery rate, were aggregated yearly. As a result, the data contains only 43 observations because only the statistical power is available on the test value level (see Table F & G in the Supplemental Material for robustness analyses of the statistical power on the test value level). The results below were presented graphically via nonparametric LOESS regression (59)

- Supplemental Material, page 18: I think the argument that not all test statistics can be found automatically, and neither can tabular results, is worthwhile and could be included in the manuscript with some elaboration. Is this a limitation of the study?

Thank you very much, this is now included as a limitation in the discussion section.

- Supplemental Material page 21: the “Publication bias and p-hacking are larger” result could be included in the manuscript.

The results for articles with less than 5 test values is now included in the robustness section.

- Throughout the Supplemental Material, some limitations to the study are mentioned. I think a limitation section, however short, is essential to include in the conclusion and discussion section.

A paragraph on limitations is now included in the discussion section.

**Minor remarks**

- 52: You state "since the pioneering study by Cohen (12: 150f.) there has been no increase in statistical power." Is this across all psychology fields?

The study of Cohen does not separate its results by subdisciplines. I nonetheless added some literature on neighboring disciplines.

Since the pioneering study by Cohen (12: 150f.) that examined the state of statistical power in leading psychological journals, the statistical power hardly increased over time and is still inadequate in the social sciences (13, 14) but also other disciplines like medicine (15-17).

- 55: I would like to see an elaboration of the difference between the FPR (False Positives / (False Positives + True Negatives)) and FDR (False Positives / (False Positives + True Positives)) here. I think the "The FDR estimates this" text threw me off when I first read it. I think it would benefit readers to also display Table S5 (Truth Table) here.

Thank you very much, I included the truth table in the manuscript and introduced the FDR in more detail.

- 59: from which field is the FDR of 14%? Does reference 16 (Ioannidis et al.) offer a better estimate of the FDR?

In his comment Ioannidis only criticized the Jager & Leek study. Because of the data limitations no alternative estimater of the FDR is presented. The Jager & Leek study is about results reported in abstracts in medical journals, I added this information in the manuscript.

- 67: I think your explanation of publication bias is very clear. However, some elaboration on p-hacking here might be interesting, since there are many different forms of p-hacking. How do these forms relate to your research?

I included a sentence to explain the forms of p-hacking in more detail:

Practices of p-hacking include thereby excluding outliers, switching outcomes, measures, or covariates to obtain a significant result (22); p-hacking is therefore very similar to the concept of selective reporting bias.

- 138: It is unclear to me what the sentence starting with "Furthermore" means.

The senctence is now pruned.

- 153: Later on in the text (around line 220) you include a short discussion on the difference or similarities in results across subfields of psychology. I think this would be appropriate here as well, and the same goes for results around line 173. I also like Figure S7 in the Supplemental Material and would like to see it included in the manuscript.

Thank you for your suggestion. In order to keep the manuscript on one common analyticallevel (discipline wide time trends) I decided not to put the subfield results in the manuscript.

- 159: What does it mean that the replication rate is nearly identical to the statistical power? The replication rate is just a subset of the statistical power limiting on significant tests only. I clarified in the manuscript:

The replication rate can be approximated based on the statistical power estimate by limiting the average statistical power on significant results (p < 0.05). Therefore, the probability that a significant effect can be repeatedly found in a similar setting is estimated. The replication rate based on the retrospective statistical power estimate is only slightly larger (44.8%) than the average retrospective statistical power. Although the replication rate is a subset of the statistical power, such a large similarity was higher than expected.

- 160: What does it mean that the time trend correlation is so large? Is this a problematic result?

This is not a problematic result (because it is just a subset, see above). However, I pruned the sentence on time trends in order to focus more on the central research outcomes, publication selection bias, statistical power and the FDR.

- 172: I would like to see more explanation on how the 22.8% is calculated (which binwidth?

does it matter?), and what it means. If I read correctly, it is the percentage of excessive significant results, assuming there is a uniform distribution?

Thank you very much, this was very short in the manuscript. I now wrote a larger paragraph in the data & methods section. Furthermore the different publication selection bias estimators for the different bandwidths (calipers) are now presented in the results section.

- 173: I would not use the term "became significant". Also on line 192.

Thanks, now changed.

- Figure 1, panel b: I do not immediately understand what the y-axis stands for. Percentage of publication bias in a field? Maybe FDR?

Figure 1 is now split up in different figures, Fig 3 & Fig 4.

- Table S7: it looks like column 4 to 7 display statistical power, because it is stated in the row above, but I think these estimates should be FDR?

Thanks for the remark Table S7 was a little bis misleading these estimates were indeed FDRs, but given the estimates of statistical power. I pruned the table, but invested more in robustness analyses of the primary estimands, the statistical power and publication selection bias.

# Reviewer 2 - André Gillibert

[shortened]

In addition, there are many minor problems with the actual structure of the manuscript that can be fixed easily: better reporting of Methods, with less reliance to Supplementary Information, a structured Discussion, including a paragraph about strengths and limitations, a comparison to the literature and research perspectives. The editorial entitled "The case for structuring the discussion of scientific papers" (BMJ 1999;318:1224) may help the author.

Thank you very much for your remarks and especially the recommended paper by Docherty (1999). I restructured the entire manuscript, now less emphasis is put on the supplemental material and more details are reported directly in the paper. Furthermore, a section on robustness analyses is now presented after the results section.

I profited a lot of the recommendations mentioned in the Docherty (1999) paper, a standardized discussion section makes absolute sense. I rewrote the discussion section accordingly.

I will first mention major issues, then minor issues.

**Major issues**

1. Hypotheses underlying the estimation of the PIC-specific pooled effects

The statistical power can only be calculated if the effect size (Cohen's *d*) is known. This effect size was calculated for each large psychology/sociology domain, assuming that all effects were identical within a field based on categories defined by the PsycINFO Classification.

This classification provides broad categories. Ten categories make approximatively 50% (figure taken from the Supplementary Information data.Rdata) of all the analysed research. "Cognitive processes", "Learning & Memory", "Cognitive & Perceptual Development", etc.

First, these categories are far too broad to assume that all effects, in primary, secondary and *post hoc* analyses, are identical. Even within an article, effects of different magnitude are expected to be tested. However, the author pooled all these effects in a meta-analysis model and used the pooled effect as reference for power analyses.

I agree, however, the alternative often done in the literature is just to assume a plausible effect size. Now I put less emphasis on just one power estimator and report also the estimators on fixed thresholds along with the criticized retrospective power estimator.

The different forms of statistical power estimators were furthermore laid out in more detail:

The true effect is, however, by definition unknown. Still, it can be approximated by a set a priori plausible effect size of interest (14), retrospectively by a meta-analysis of relevant studies (17, 37, 38), or post hoc using a transformation of the reported p-value for power analyses (for the terminology cp. 39). While the first approach, a priori power analyses, may suffer from a hypothetical bias, retrospective power analyses root the estimate in the empirical literature if meta-analyses are applicable (40). Although post-hoc power is used in similar analyses (41), they tend to understate statistical power and add little information because they are directly calculated from p-values (39, 42).

However, the variance of effect sizes is important to take in account. For instance, assuming that the thousands tests were all adequately powered (80% every time) but that half of the effects tested were very small, associated to very large sample sizes (to get 80% power), and half of the effects were very large, associated to small sample sizes (with 80% power), then, the overall statistical power would be 80%, but the methodology of this manuscript would find that half of the studies are underpowered (power ~ 5 or 10%) and half are overpowered (power >99%) with an overall power at 40-50% because it would find an intermediate pooled effect size. Therefore, the pooled effect size is not enough: its variance must be assessed. That is possible thanks to the tau² statistic that meta-analyses model can compute. However, the covariance between the effect size and sample size must be computed to reliably assess the overall statistical power, which seems to be impossible.

I also agree with this suggestion and integrated a retrospective statistical power estimate that accounts for effect heterogeneity by using the suggested tau² from random-effects meta-analyses.

Another problem exists. Not all tested hypotheses are true. If half of the hypotheses are true, the pooled effect size would be diluted by a factor two, ie. grossly underestimated. Therefore, the pooled effect size must have a correction factor, based on the hypothetical rate of true hypotheses, which is unknown.

I somewhat struggled with this suggestion. Although the underlying effect is non-zero, a research hypothesis still can be wrong (e.g. in a different population, using slightly different methodology) – in short, all factors that contribute to the between-study heterogeneity. As standard errors should not differ between true and false hypotheses, the only reason for an underestimated power would be, as you correctly note, a underestimated true effect. Given the on average the estimated mean effects are around d~0.5, they are already seem quite large. Assuming just a priori a e.g. small effect, in my opinion, makes things worse because available (although diluted and noisy) information is discarded. The concept of retrospective power analyses is also used e.g. in economics (Ioannidis JPA, Stanley TD, Doucouliagos H. 2017).

The R code and table S4 show how effect sizes (Cohen's d) are calculated from P-values. These calculations are only based on the type of statistic (eg. t test) and degrees of freedom. This is at unreliable, if not wrong. Whether paired or two-samples t tests are used is unknown. The group balance of two-samples t tests is also unknown. The formula used by authors is correct only if the test is a two-sample t test and both groups are of equal size. If actual groups are unbalanced, the formula underestimates the effect size. If a one-sample t test is used, the underestimation is even worse, because there are twice as many measurements as degrees of freedom, and measurements are correlated, which is not taken in account by the formula. The same consideration applies to F tests: unbalance or pairing (typically in mixed effects models) greatly change the relationship between the F tests, degrees of freedom and effect sizes. Although slightly different, the problem of group balance and pairing (McNemar) also exists for chi-square tests.

Thank you very much, indeed the assumptions were underspecified in the manuscript. If t-values and degrees of freedom are available, in my understanding, one-sample and two-sample test lead to the same effect size, the one-sample test would, in this case show a doubled degree of freedom.

The problem with unequal group size could indeed not be resolved. Concerning the problem of paired data or unpaired data, the used formulas assume paired data. The risk of an underestimation of the effect size should therefore be as minimal as possible.

Calculations based on the Pearson correlation coefficient and a biserial correlation coefficient are reported in the robustness section to test the robustness in respect to the used effect size metrics. The results are, with the exception of r, quite similar.

Concerning the assumptions, it is now clarified:

For these subfield-specific meta-analyses, all test values were transformed in Cohen's *d* metric using the transformation formulas for paired tests (44: 524, 45: 35, for further details, see Section 2.2., Supporting Information) because this information could not be retrieved from the test values. However, this assumption is conservative as the effect size would be approximately cut half in case of unpaired effect sizes (40). For tests with a discrete (grouping) variable (*F*, *t*, *χ²*), equal group sizes have to be assumed because no group size information was available. This assumption is more problematic as potential differences in group sizes may underestimate the effect of the study. However, given the large share of experimental studies where randomization provides at least approximately equal group sizes should at least mitigate the problem (for robustness tests for different test statistics see the robustness section).

In the robustness section it is clarified:

Although the assumptions needed for the test value calculation and computation of the statistical power could not be directly tested, it is possible to look at the results for the different test statistics. As r, the Pearson correlation coefficient, does not suffer from restrictions (pairing), it is used as a benchmark (retrospective power ~55%). Overall the statistical power estimates were lower for χ²- and t-tests. In the case of t-tests, the lower statistical power (around -20 percentage points) can be caused by the assumption that always two-sided tests were used. For χ²-tests (especially with df>1, nearly -30 percentage points), manual coding shows a large share of diagnostic tests that may result in lower effect size estimates. For F-tests, the most used test statistic in the data, no large differences (<10 percentage points) were found in the case of df1=1 while tests with df1>1 even show a larger estimate of statistical power (around +12 percentage points). Therefore although a bias due to the assumptions of the effect size transformation cannot be ruled out, they should only lead to a minimal bias in the statistical power estimates.

From the study database (data.Rdata), approximatively 25% of all tests are F-tests comparing three groups or more. The concept of Cohen's d is questionable when there are more than two groups. Although the concept of statistical power remains, pooling three-groups effect sizes with two-groups effect sizes is questionable. Once again, balance between groups and absence of pairing is assumed by author's calculations.

I completely agree, see above for respective robustness analyses.

The manuscript mentions that "*The first indication of publication bias and/ or p-hacking was the large share of statistically significant findings (72.8% at the 5% level) in the examined 170 literature, especially in the context of the low statistical power*."

In the discipline of psychology this is often used as some kind of a "first evidence" on publication selection bias (Sterling 1959, Sterling et all 1995). However, I fully agree with your point and pruned the respective results from the manuscript.

In my opinion, that may be mainly explained by the wrong hypotheses underlying the assessment of the effect sizes and statistical power. I think that it is mainly explained by a dispersion of z-values much larger than modelled, because effect sizes are not constant within each research field. Indeed, even if publication bias and p-hacking were massive, it would artificially lead to overestimation of the true effect sizes by the meta-analysis algorithm and so, would not make that much a difference between the computed statistical power (44.6%) and proportion of statistically significant findings (72.8%).

Due to all these limitations, I recommend that the author of this manuscript do not put forward results relying on the pooled effect sizes and that they clearly announce the limitations of their approach.

I completely agree, now all 5 possible estimators: retrospective power, + heterogeneity, small a priori effect, medium a priori effect & large a priori effect are reported in the manuscript. As mentioned before, although the limitation of retrospective power-analyses that rely on the subfield meta-analyses, I think just relying on the a priori information would be a not justifiable loss of information.

2. Overinterpretation of publication bias

The publication bias was assessed by a reliable method, independent of the statistical power or distributional assumptions. The ρ statistic (ρ=s/0.5-1) is based on the ratio s between the number of P-values between 0.0396 and 0.05 and P-values between 0.0396 and 0.0626. This seems to have been interpreted as an approximation of the rate of p-hacking/publication bias that transform any non-significant (eg. p=0.30) result to a significant result (eg. p=0.045). I think that this may be a strong overestimation. Indeed, the peak of values between 0.0396 and 0.05 may include p-hacked P-values that were initially at 0.06, but it also includes p-hacked P-values that were between 0.06 and 1.00. Assuming that 10% of tests under null hypothesis with P-value between 0.05 and 1.00 are p-hacked to make them enter the [0.03-0.05] range according to a uniform distribution, there will be 2% of results "naturally" between 0.03 and 0.05, plus 10% artificial. Between 0.0396 and 0.05, there will be approximatively half of them ((10%+2%)=6%), compared to 0.9×1.26%=1.13% between 0.05 and 0.626. The ρ statistic will be 6/(6+1.13)/0.50-1=68.3%, much higher than the 10% of p-hacking of the hypothesis. Unfortunately, the real publication bias or p-Hacking cannot easily be inferred from the ρ statistic because it depends on several unknown parameters. First, the correlation between the P-value and p-hacking. A P-value at 0.07 may be p-hacked more frequently than a P-value at 0.50. Second, the distribution of P-values after p-hacking. What is the proportion between 0.04 and 0.05? Between 0.01 and 0.04? Less than 0.01? Third, the process of the publication or selective reporting bias. The publication bias may be higher for P-values far from 0.05 than P-values close to 0.05. At least, the publication bias follows a more predictable scheme, because it ultimately leads, under the null hypothesis, to a uniform distribution of P-values between 0 and 0.05.

I agree in principle with your points. The core problem, in my opinion, is that the black box of publication bias, so the process and effectivity of engaging in such practices is not estimable. In the simulation I conducted using largely the same setting as in one of my previous papers on the accuracy of publication bias tests (Schneck 2017), no bias of the estimator showed up for. In your example, the bias would also vanish if a true underlying effect (which should also be the case given the data) I present. In the limitation paragraph in the discussion section I therefore added:

Fourth, although the estimator of publication selection bias performed well in a simulation study, it may depend on the process (possibly nearly significant results are more at risk of publication selection bias) and effectivity (how easily a desired significant results can be obtained) of publication selection bias. Although the article gives a plausible range of statistical power and publication selection bias estimators, the exact results should be interpreted with caution.

Therefore, the ρ statistic, is a poor estimator of the proportion of null results that are artificially made positive by p-hacking or publication bias. It is used to estimate the FDR_inf_ in Fig 2. I propose to provide sensitivity analyses with different ρ values.

Thank you very much for this very helpfull suggestion, I included Fig 7 for robustness analyses varying publication selection bias from 0-100%.

3. The FDR depends on the proportion of true hypotheses

That is well described by the author of this manuscript and several scenarios are tested: 50%, 20% and 10% of true hypotheses. The distribution of P-values may help to assess the FDR. Indeed, if 5% of P-values were below 5%, one could easily tell that most discoveries are false. If 95% of P-values were below 0.0001, that would be indicative of true discoveries. Nevertheless, the difference between p-hacking, publication bias and true discoveries can hardly be made.

I fully agree. The p-values of the papers were due to this fact not interpreted as any indicative evidence.

4. The difference cannot be made between primary and secondary analyses

Sometimes, some secondary tests have very little power. Sometimes, the null hypothesis is even true, eg. when comparing characteristics of two randomized groups. Other times, secondary tests may be "knocking on an open door", with a very high power. The results of these tests are of minor interest. They also may violate the hypothesis of "constant effect size" that has been made.

The author approached the concept of "primary analysis" by performing a subgroup analysis of articles with five tests or fewer. I fear that such articles may contain more tests, but the algorithm could not track them because they were not reported with the APA style. May the author verify that fewer than five tests were performed on a subset of articles, by human reading of HTML or PDF files?

I do not think that there is a solution to that problem, but it should be mentioned in the discussion.

This problem indeed cannot be solved. I conducted the requested robustness check (section XY in the supplemental material). In short: the automatic extraction process of test values could not replace by hand coding of primary research outcomes in meta-analyses. In total 25% of all relevant in text or tabular primary outcomes were covered, while 50% of the covered values were actually no primary research outcome.

In the Supporting Information it is therefore clarified:

So far, it could be shown that the automatic export routine covered nearly all test values reported in the articles and overall a large share of test values. Nothing, however, can be said about the relevance of the test value for the primary research outcome. Therefore, as a second robustness check of the data extraction process, a substantial coding of 25 articles was also conducted in the population of articles that report up to five test values. This manual coding extracted only test values that were directly linked to the primary research outcomes. Robustness and manipulation checks were therefore not extracted in the manual coding but were extracted in the automatic procedure. The limitation of the subset of 5 articles was chosen because only this subset gave hope to be related to primary research outcomes only. In total, the automatic approach could not extract primary research outcomes needed for topic-interested meta-analyses automatically. In total, only 25% of all primary research outcomes could be extracted. Furthermore, of the exported values, 49% of all values are not primary research outcomes. Although these robustness analyses show that the automatic extraction procedure is not suited for topic-related meta-analyses, they may plausibilize an average effect needed to calculate the statistical power. For the analysis of publication selection bias, this poor coverage may not affect the estimator because secondary research outcomes can also be prone to publication selection bias.

**Minor issue: methods**

The R script supports some deviations to APA style but generates wrong results in some cases! Moreover, it may support more deviations.

Examples:

“Chsq(54,N=10,800)” should be interpreted as Chsq(54,N=10800) but has been incorrectly interpreted as Chsq(54,N=10).

"F(4,752,9,504)=13.06" should be considered as ill-formed but has been incorrectly interpreted as F(4,752)=13.06

"F(1,,70)=4.28" has been ignored but could be interpreted as "F(1,70)=4.28"

"Chsq(1,332,N=591)=1673" has been interpreted as "Chsq(1,N=332)=1673" but is ill-formed unless some reliable meaning can be found.

My personal suggestion is to identify well-known patterns with strict regular expressions, then, check what has not matched and find new regular expressions that exclude or correctly interpret the new patterns; then iterate until all tests have been classed in one of the two categories: "well-defined meaning" and "ill-formed". R warnings should not be ignored.

Many thanks, as suggested I now use explicit regular expression for the test value processing. The exported results were also double-checked to exclude falsely detected test values.

**Minor issues: reporting of methods**

Although many Supporting Information is provided, the exact references to articles seem to have be anonymized with identifiers such as "E1974_1984#1" containing a period and an index number. Why not providing the Digital Online Identifier (DOI), a Uniform Resource Locator (URL), or an identifier that makes it possible to retrieve the article?

My intention for omitting the DOIs in the dataset was to protect authors from single studies from being blamed for low power or "too many" studies in an over-caliper (see e.g. Francis 2012 for such analyses). Considering your comment, I included the values in the data to make the results as reproducible as possible.

Literature:

Francis, Gregory. 2012. "Too Good to Be True: Publication Bias in Two Prominent Studies from Experimental Psychology." Psychonomic Bulletin & Review 19(2):151-56. doi: 10.3758/s13423-012-0227-9.

Most of my understanding of the methodology of the article comes from the Supporting Information. The Methods or Result section should contain the following ideas that are required to understand the article:

1) Test statistics were extracted from the full-text of articles by regular expressions
2) Most full-text articles were in HTML format (~96%); but the oldest and newest could be in PDF format; OCR was used in PDF scans.
3) Test statistics in tables were not extracted because they generally do not follow the APA style
4) The APA style is typically test(degrees of freedom)=value where test can be "F", "Chisq", "t".
5) Sample sizes could not be calculated for t tests and F tests; therefore, it was assumed that they were about equal-sized independent groups.
6) Z-tests were ignored.

I clarified according to your suggestion in the data section.

The Caliper Test (CT) is well described but the conversion from z-values to P-values should be given to make things easier to understand for the reader.

Many thanks for the suggestion, the according p-values are now reported along the calipers.

The 5%-caliper offers a trade-off of both criteria as shown in simulations (53) and contains the just significant *p*-values in the range [0.039, 0.05) and the just non-significant *p*-values from [0.05, 0.0626). However also the 10%- ([0.0311, 0.05), [0.05, 0.078)) and 1%-caliper ([0.048, 0.05), [0.05, 0.052)) were estimated.

Since effects are actually not Cohen's d for 3-sample F tests and paired t tests, they should not be called like that. The term "effect size" may be preferred.

I avoided the term Cohen's d in the manuscript whenever possible to avoid false interpretations. When related to the effect size formulas, I referred to as "Cohen’s d metric”.

Please, clarify inclusion/exclusion criteria. The criteria “*articles that followed the strict reporting guidelines published by the APA in 1974.*” seems to be inconsistent with the Supplementary Information. Indeed, from the R script, it seems that some ill-formed test statistics are found in articles without the whole article being excluded: only the test seems to have been excluded. Moreover, some common deviations to the APA style were correctly detected and interpreted. This is well-specified in the Supplementary Information PDF “Small deviations from the publication manual were also covered.” but contradictory to the inclusion/exclusion criteria.

Additional exclusion criteria were identified in the R script, in a section called “Exclusion criteria”. These should be succinctly reported in the Methods section. Again, they seem to apply to tests and not articles.

I corrected in the article:

The used automatic extraction was able to cover all reported test values in the articles' text that followed the strict reporting guidelines published by the APA in 1974 (31) or later editions. This reporting guideline strongly recommends the in-text presentation of test values as a primary option if the test values are crucial to support the study's conclusions (32: 116f.). A systematic extraction via regular expressions was possible because the APA publication manual has a strict reporting scheme that includes the kind of the test statistic (F, χ², r, t, z), its respective degree(s) of freedom, and its test value (e.g. F(1,4) = 3.25). An exception to this reporting style is the mandatory sample size (N) after the degrees of freedom for the χ²-test until the 6th revision of APA-style (33-36). Also, some minor deviations in the articles were allowed (e.g., X² instead of χ²). Besides reporting test values directly in the articles' text, the publication manual also allows tables to present results from more sophisticated research designs (33: 39). However, in the 6th edition of APA-style, tables and figures are recommended for articles reporting more test values (>4 and >20 respectively 32: 116). In contrast to the in-text reporting, it was impossible to extract results reported in tables due to diverse reporting styles (e.g., standard errors or p-values below or beside the reported estimates). The in-text presentation of crucial results is, in contrast, consistent and varies only slightly over time (e.g. N reported along χ²).

The calculation of the False Discovery Rate (FDR) relies on the assumption that all tests are independent. Although this assumption is minor compared to others, it should be mentioned to be rigorous.

Added in the paper:

An additional assumption is the independence of tests, meaning that there are no (or not substantial) exact re-analyses included in the data (studies using the same data and the same or at least largely similar analysis procedure). Given that even replications are hard to publish in psychology (56), this should be of no or negligible concern.

A reference to the APA style guideline should be added at the first mention of this style.

Corrected in the manuscript.

**Minor issues: reporting of Results**

Please, add a flow chart to the manuscript, ideally as figure 1. I suggest that both the number of tests and articles be reported in each box of the flow chart. The flow chart should be cited at the first paragraph of the Results section.

Thank you very much for this suggestion, I added the respective (consort-style) flowchart at the beginning of the result section (Fig 2)

It is written that “*Overall, only 17.8% of the studies were adequately powered (>80%), although this share increased to 28.3% in 2017*”. Is 17.8% the proportion of adequately powered **tests** or **studies**? If this is about studies, how are all tests of a single study aggregated for the power calculation?

Thank you, this was misleading in the manuscript, of course, in this context, only tests can be adequately powered.

Overall, only 20% of the tests reached a retrospective statistical power larger than 80% and were therefore adequately powered.

It is mentioned that “*The replication rate is, however, with 45.6% nearly identical with the statistical power.*” but it is unclear how it has been calculated. Is it calculated for each test, as the probability that a replicated study with the same sample size leads to a significant result conditional to the fact that the first study had a positive result, whatever the null or alternative hypothesis is true? Does it take in account the p-hacking and publication bias in the original study? In the replication study? Does it take in account the Bayesian probability that the alternative hypothesis is true conditional to the success of the first analysis? This should be presented in Methods.

The replication measure does control for publication bias (same as the retrospective statistical power). I clarified in the manuscript:

The replication rate can be approximated based on the statistical power estimate by limiting the average statistical power on significant results (p < 0.05). Therefore, the probability that a significant effect can be repeatedly found in a similar setting is estimated. The replication rate based on the retrospective statistical power estimate is only slightly larger (44.8%) than the average retrospective statistical power. Although the replication rate is a subset of the statistical power, such a large similarity was higher than expected.

The sentence “*This also applies to the time trend (r = 0.99).*” is unclear. What are the two variables correlated?

The sentence referred to the two time trends of statistical power and the replication rate. Because one is a subset of another it does not contain much information. I pruned the sentence from the manuscript.

The sentence “*Low statistical power also has the deterrent side effect of fostering publication bias or p-hacking because it is harder to detect existing effects. This may encourage authors to engage in publication bias and/ or p-hacking to find an existing effect by repeated testing until they reach statistical significance.*” is inappropriate for a result section and should be moved to the introduction or completely removed, since it does not contain any result.

The sentence is now pruned.

**Minor issues: Supplementary Information (PDF)**

**Table S7:** the bottom legend of the table suggests that the table contains values of “FDR” but the columns titles, at the top of the table, suggests that the table contains values of “Statistical power”. The consistency should be fixed.

Thanks for the remark Table S7 was a little bit misleading, these estimates were indeed FDRs, but given the estimates of statistical power. I pruned the table but invested more in robustness analyses of the primary estimands, the statistical power and publication selection bias.

The two following sentences are hard to understand: “*Until the year 1986, all texts retrieved were only available in PDF format. In the following years, this share drastically drops to nearly zero. In the most recent years, 2016 and 2017, 42.2% and 10.6% of the texts were only accessible in PDF format.*”

The sentence is now pruned.

The second sentence seems contradictory to the first one, but a re-analysis from the study database makes everything clear: The PDF share dropped between 1986 and 2015 and strangely raised in 2016 and 2017. The reason of this raise should be explored: new journals included? Time to convert PDFs to HTMLs? The sentences should be rephrased to make things clearer, such as replacing “*In the following years*” by “*from 1986 to 2015*” and replacing “*In the most recent years, 2016 and 2017*“ by “*This share greatly increased in 2016 and 2017*”.

The sentence is now pruned, as the information is now located in the main text.

**Minor issues: introduction**

Please write complete sentences in plain English, avoiding ambiguous parentheses. How can I interpret the sentence: “*Severe misconduct, such as fraudulently making up whole datasets (2), and minor misconduct, such as (deliberately) erroneous rounding results*”. Either remove the word “deliberately”, or remove parentheses or explicitly rephrase “*minor misconduct, such as deliberate or inadvertent erroneous rounding of results*”.

Sentence changed to:

Although severe misconduct, such as fraudulently making up whole datasets, seem to be rare (5), questionable research practices, such as erroneous rounding results (6-8) and selective reporting of results that confirm the expectations (8, 9), may pose a larger problem on the validity of the findings.

The concept of “selective reporting bias” may be mentioned in addition to publication bias. Although both may be confounded, the publication bias is about not publishing the article at all while the selective reporting bias is about not reporting non-significant P-values in published articles.

Thank you very much for your suggestion. In the revised version I introduce the term publication selection bias for publication bias and p-hacking. Selective reporting bias is a concept very similar to p-hacking. I therefore clarified:

p-hacking is, therefore, very similar to the concept of selective reporting bias.

Some sentences are awkward, such as “*Because minor forms of scientific misconduct are much more common than fraud (7), they erode scientific integrity in society more severely*”. It basically tells that scientific misconduct (ie. lack of scientific integrity) erodes scientific integrity? That is a tautology.

Sentence changed, see above.

“*Statistical power [...] is a quality criterion of good research practice and should be as high as possible.*”. Please, either remove the term “*quality criterion*” or “*practice*”. Please, also remove the words “*and should be as high as possible*”, since it is contradictory to the next sentence that mentions that one should “*keep the included number of cases small (e.g. to minimize harm to participants or fit budget constraints)*”.

Sentence is now pruned.

Assertions without solid data to backup should not be written as assertions. For instance “*Finally, results driven by scientific misconduct shape the public discourse and ultimately drive (political or medical) interventions that might do more harm than good (8).*” is supported by a reference that shows an expert’s opinion without underlying data. The words “and *ultimately drive*” should be replaced by “**may** ultimately drive” or any other formulation that adds uncertainty to the assertion. The last sentence of the introduction “*Instead of rejecting theoretical predictions, so-called "undead theories" with no evidential value survive (24) and inhibit any scientific progress.*” suggests that there is no scientific progress at all due to false undead theories. The word “*any*” should be removed.

The respective sentences have been changed as suggested or pruned.

Some approximations are done: “*This article shows the development of statistical power, publication bias and p-hacking as forms of scientific misconduct*”. Statistical power is not a form of scientific misconduct. **Insufficient** statistical power may be caused by scientific misconduct, namely by a lack of scientific rigor and methodology in the assessment. However, insufficient statistical power, per se, is not a scientific misconduct.

Changed to:

The article at hand shows the development of statistical power and publication selection bias in articles published in psychology between 1975 and 2017.

Some sentences are redundant such as “*From a resource-focused perspective, low statistical power is inefficient because random variations in the data disguise the underlying effect patterns.*” and “*Therefore, the noise of the falsely detected estimates, as defined by the significance threshold (false-positive rate: FPR = e.g. set at 5%), dramatically impacts the overall significant results.*”. The former may be removed without loss of information. The latter could be more rigorous. Indeed, the FPR is not exactly equal to the significance threshold if tests are correlated it could be rephrased to “*(equal to the false-positive rate if tests are independent: FPR = e.g. set at 5%)”* or the FPR could be mentioned in a separate sentence.

The first sentence has been pruned, the second changed to:

In this situation, the noise of the falsely detected estimates (second column in Fig 1), defined by the significance threshold (equal to the false-positive rate - FPR: e.g., set at 5%), dramatically impacts the overall significant results.

A few words should be added to mention the aspects that make this work original compared to previous works.

At the end of the introduction, the core research aim has been added:

Therefore, the core research aim is to answer the question of John Ioannidis, “why most published research findings are false” (2), empirically.

Also in the discussion, a paragraph on the strength has been added :

Because the article draws from a full sample of empirical articles in journals published by the APA and the therein reported statistical test values, one conceptual and two data-related strengths of the analysis can be pointed out. The conceptual strength is to measure statistical power and publication selection bias independently in one dataset to shed light on at least two variables needed to calculate the FDR. This approach has the advantage that only the by definition unknown share of true hypotheses remains unknown. The two data-related strengths are the high statistical precision due to many included studies and test values and a good external validity, which allows for a generalization over psychology.

**Minor issues: discussion**

The discussion is not enough related to the article content. The first paragraph, summarizing the main results is appropriate but may be improved. For instance, the first sentence “*The article has presented evidence on the development of three scientific integrity measures: statistical power, the prevalence of publication bias and p-hacking, and the FDR.*” may be rephrased to suggest that statistical power is a quality criterion of scientific research but not necessarily a measure of scientific integrity. The word “*are*” should be replaced by “could be” in “*The resulting FDRinf illustrates the consequences, as roughly one-third of all significant results are just statistical artifacts*”. There are too many limitations and hypotheses to provide a clear-cut conclusion.

The rest of the discussion makes recommendation based on the author opinion. I suggest to rewrite it entirely, based on BMJ’s guidelines (BMJ 1999;318:1224).

Many thanks for this very helpful suggestion; the discussion has been rewritten according to the abovementioned suggestions.

**Suggestions: additional robustness and subgroup analyses**

I am curious to know whether results depend on the type of test (chi-square, t, F, etc.)?

This information is now included in the robustness section in the manuscript and Table C in the Supporting Information (Appendix).

A robustness analysis could be performed to compare the quality of PDF and HTML files. That is especially useful to interpret the latest results (2016 and 2017). Are there the same number of tests detected by article in PDF and HTML files? Are other metrics (power, publication bias, FDR) similar?

Also added in the robustness section and Table C in the Supporting Information (Appendix).
