# Supplementary material for: Are most published research findings false? Trends in statistical power, publication selection bias, and the false discovery rate in psychology (1975–2017)
Source: PLoS One. 2023 Oct 17;18(10):e0292717. doi: 10.1371/journal.pone.0292717 (PMC10581498; doi:10.1371/journal.pone.0292717)
Supplement: Attachment — Submitted filename: Reviewing.docx [file pone.0292717.s006.docx]

I thank authors of the major improvements they made in the manuscript, trying to answer every concern reviewers had. There were massive changes in the manuscript, showing improvements in all areas that reviewers have commented. I have only one major concern remaining, and several minor concerns.

# Major concern

The major concern is about the interpretation and use of the rho statistic (publication selection bias). The issue is much more complex than I initially thought, so I will be verbose. I am sorry, but I hope that you will appreciate that level of detail. I found that, p-hacking and publication bias leads to very different estimates of the FDR, and the theoretical model of “publication selection bias” is crucial but totally unknown.

The rho statistic is interpreted as the proportion of non-significant tests that become significant due to p-hacking or publication bias. However, I think that this is not a correct interpretation. This can be easily be understood under the scheme of “pure” publication bias, without any p-hacking. We will assume that the chance of having a significant result at each test is 50% (the average statistical power you found). If 37.0% of non-significant tests are dropped (publication bias), without any p-hacking or replication of the study, the total number of published P-values (denominator) will be reduced by 37.0% * 50% = 18.5% while the number of published P-values in the range [0.05 – 1.00[ will be reduced by 37.0% without any change of the number of published P-values in the range [0.00 – 0.05[. Consequently, the distribution of P-values will be changed. The density probability of P-values in the range [0.00 – 0.05 [ will be homogeneously multiplied by 1/(1-0.1850) = 1.227 (i.e. +22.7%) because of the reduction of the total number of published P-values (denominator) while the density probability of P-values in the range [0.05 – 1.00[ will be homogeneously multiplied by (1-0.370)*1.227 = 0.773. These two changes in density probability will create a discontinuity at the 0.05 threshold. You defined the rho statistic as P(x_z_=1)/0.5-1. In that example, P(x_z_=1) = 1.227/(1.227+0.773) = 0.6135 so that rho=0.227. Therefore, the rho statistic you found is equivalent to a dropping of 37.0% of non-significant results. This does not make them significant but make them disappear in the void. What is the impact on the FDR? The total number of true positives (TP) would be unchanged (because significant results are not dropped by the publication bias) and the total number of false positives (FP) would also be unchanged (because significant results are not dropped by the publication bias) while the number of false negatives (FN) and true negative (TN) would be both decreased. The FPR (FP/(FP+TN)) would be inflated but the FDR (FP/(TP+FP)) would be unchanged! That may seem incredible at the first glance, but look at Fig. 1 of your article and do the calculation! This can be explained because the false discovery rate is only computed on the subset of significant results. This subset is not changed by the publication bias. Only non-significant results are dropped. Even if the drop rate differs between true and wrong hypotheses with non-significant results, the FDR is still unchanged by the publication bias.

Actual publication biases are much more complex than the simplified “pure” model I described above. Indeed, significant results that are found in an unexpected direction, may also be dropped. For instance, results showing that a drug is significantly worse than the placebo. Dropping these significant results will increase the number of false positive (FP) and so, increase the FDR.

Moreover, any form of p-hacking can greatly increase the FDR. Indeed, p-hacking changes non-significant results (such as true negatives) into significant results (such as false positives). I will show that, two different models of p-hacking leads to very different calculations of the FDR.

The first model of p-hacking is that a proportion P of non-significant results is changed to become significant by p-hacking. The proportion of such p-hacking is assumed to be independent of the initial P-value, as long as it is in the range ]0.05 – 1.00]. Assuming that the p-hacking is equivalent to the process of drawing P-values from a uniform distribution on [0.00 – 1.00] an unlimited number of times, until the P-value is in the range [0.00 – 0.05], the p-hacked P-values would follow a uniform distribution on the range [0.00 – 0.05]. Now, assume that half of the hypotheses are true (theta=0.50), the statistical power is 50% for true hypotheses (pow), and that 9.5% (p-hacking rate or PHR) of non-significant results are p-hacked, independently of the fact that hypotheses are true or wrong. The very simple calculator-like R code below shows how to calculate the FDR:

PHR=0.095

power=0.50

theta=0.50

base_FP = (1-theta)*0.05

extra_FP = (1-theta)*(1-0.05)*PHR # extra number of FP due to p-hacking

total_FP = base_FP+extra_FP

base_TP = theta*power

extra_TP = theta*(1-power)*PHR

total_TP = base_TP+extra_TP

FDR = total_FP/(total_TP+total_FP)

With a p-hacking rate at 9.5%, the FDR is 20.4% while the FDR would be only 9.1% with no p-hacking at all. I tried to compute the rho statistic from the p-hacking rate, but found that it was impossible without extra hypotheses. Indeed, the functional relationship between the PHR and the rho statistic depends on the variance of the power. Assuming the power is homogeneous among all tests, the rho statistic can be calculated for a specified PHR. The following R source code shows a computation of rho by simulations, that should be sufficiently easy to understood with comments:

```
simu=function(theta, power, PHR) {
	# simulate a test with p-hacking
	# on input:
	# theta = prior probability that each hypothesis is true
	# power = statistical power of true hypotheses

	H=rbinom(1,1,theta) # set to 1 if the hypothesis is true
	if (H!=0) {
		# if the hypothesis is true, draw a first non-p-hacked p-value
		pvalue = pnorm(rnorm(1,mean=qnorm(0.975)+qnorm(power)))
		# transform one-sided p-value to two-sided p-value
		pvalue = 2*pmin(pvalue, 1-pvalue)

		# if the p-value is non-significant but a p-hacking is done
		if (pvalue >= 0.05 && rbinom(1,1,PHR)!=0) {
			# we draw p-values repetitively until one is significant
			while (pvalue >= 0.05) {
				pvalue = pnorm(rnorm(1,mean=qnorm(0.975)+qnorm(power)))
				pvalue = 2*pmin(pvalue, 1-pvalue)
			}
		}
	} else {
		# draw a p-value under the null hypothesis
		pvalue = runif(1)
		# if the p-value is non-significant but a p-hacking is done
		if (pvalue >= 0.05 && rbinom(1,1,PHR)!=0) {
			# draw a p-hacked p-value under the null hypothesis
			pvalue=runif(1,max=0.05)
		}
	}
	# return whether the hypothesis is true or wrong and the p-value
	return(c(H,pvalue))
}

compute_rho=function(theta, power, PHR, caliper=0.05, nsimu=1000000) {
	# define the threshold of P-values for the specified caliper
	pvalue_middle = 0.05
	pvalue_low = 2*(1-pnorm(qnorm(0.975)+caliper))
	pvalue_high = 2*(1-pnorm(qnorm(0.975)-caliper))

	# do many simulations of partially p-hacked p-values
	v=replicate(nsimu,simu(theta,power,PHR))
	# compute the number of p-values slightly below or above
	# the 0.05 threshold
	pbelow=sum(v[2,]>pvalue_low & v[2,]<pvalue_middle)
	pabove=sum(v[2,]>pvalue_middle & v[2,]<pvalue_high)
	# compute the rho statistic
	rho = (pbelow/(pbelow+pabove))/0.5-1

	return(rho)
}

set.seed(2021)
compute_rho(0.50, 0.50, 0.095)
```

This shows that rho=22.5% (value is not exact due to a finite number of simulations) for a p-hacking at 9.5%, power at 50% and theta at 50%. The same calculation can be obtained without simulation (if you find that clearer):

```
rho=function(theta, power, PHR, caliper, alpha=0.05) {
	pvalue_middle = alpha
	# caliper around the 0.05, expressed on p-value scale
	pvalue_low = 2*(1-pnorm(qnorm(1-alpha/2)+caliper))
	pvalue_high = 2*(1-pnorm(qnorm(1-alpha/2)-caliper))

	# probability of H0 being true and a just-significant result, without p-hacking
	pbelow_H0_unhacked = (1-theta)*(pvalue_middle - pvalue_low)

	# increase in that probability due to p-hacking
	pbelow_H0_hacked   = (1-theta)*(pvalue_middle - pvalue_low)*(1-alpha)*PHR/alpha
	pbelow_H0 = pbelow_H0_unhacked + pbelow_H0_hacked

	# probability of H0 being true and a just-non-significant result
	pabove_H0 = (1-theta)*(pvalue_high - pvalue_middle)*(1-PHR)

	# expectancy of Wald's z statistic under H1
	z = qnorm(0.975)+qnorm(power)

	# probability of just-significant result under H1, without p-hacking
	pinterval_below = (pnorm(z+caliper - qnorm(0.975)) - pnorm(z - qnorm(0.975)))

	# probability of just-non-significant result under H1, without p-hacking
	pinterval_above = (pnorm(z - qnorm(0.975)) - pnorm(z-caliper - qnorm(0.975)))

	# probability of H1 being true and a just-significant result, without p-hacking
	pbelow_H1_unhacked = theta*pinterval_below
	# increase in that probability due to p-hacking
	pbelow_H1_hacked = theta*(1-power)*PHR*pinterval_below/power
	pbelow_H1 = pbelow_H1_unhacked + pbelow_H1_hacked
	# probability of H1 being true and a just-non-significant result
	pabove_H1 = theta*pinterval_above*(1-PHR)

	# unconditional probability of a just-significant P-value
	pbelow = pbelow_H0+pbelow_H1
	# unconditional probability of a just-non-significant P-value
	pabove = pabove_H0+pabove_H1
	# rho statistic
	rho = (pbelow/(pbelow+pabove))/0.5-1
	return(rho)
}
rho(0.50, 0.50, 0.095, 0.05)
```

It finds that rho=22.7% for a p-hacking at 9.5%, power at 50% and theta at 50%.

Another method of p-hacking is possible : making almost-significant results (0.05 < p < 0.08) become significant (0.04 < p < 0.05), but not modifying any p-value greater than 0.08. That method of p-hacking is illustrated by the following simulation code:

```
simu_phacking2=function(theta, power, PHR, min_phack=0.05, max_phack=0.08, target_phack_min=0.04) {
	H=rbinom(1,1,theta) # set to 1 if the hypothesis is true
	if (H!=0) {
		pvalue = pnorm(rnorm(1,mean=qnorm(0.975)+qnorm(power)))
		pvalue = 2*pmin(pvalue, 1-pvalue)

		if (pvalue >= min_phack && pvalue < max_phack && rbinom(1,1,PHR)!=0) {
			pvalue = runif(1, min=target_phack_min, max=0.05)
		}
	} else {
		pvalue = runif(1)
		if (pvalue >= min_phack && pvalue < max_phack && rbinom(1,1,PHR)!=0) {
			pvalue = runif(1, min=target_phack_min, max=0.05)
		}
	}
	return(c(H,pvalue))
}

FDR_of_simus=function(v) {
	H0=!v[1,]
	significant = v[2,] < 0.05
	mean(H0[significant])
}
rho_of_simus=function(v, caliper=0.05) {
	pvalue_middle = 0.05
	pvalue_low = 2*(1-pnorm(qnorm(0.975)+caliper))
	pvalue_high = 2*(1-pnorm(qnorm(0.975)-caliper))

	pbelow=sum(v[2,]>pvalue_low & v[2,]<pvalue_middle)
	pupper=sum(v[2,]>pvalue_middle & v[2,]<pvalue_high)
	(pbelow/(pbelow+pupper))/0.5-1
}

set.seed(2021)
simus = replicate(10000000,simu_phacking2(0.50,0.50,0.154))
FDR_of_simus(simus)
rho_of_simus(simus)
```

This shows that, with a theta=50%, power=50%, and 15.4% of P-values between 0.05 and 0.08 p-hacked to become P-values between 0.04 and 0.05, the rho statistic is equal to 22.7% and the FDR is 9.6%, slightly higher than the FDR of 9.1% without p-hacking.

Therefore, it seems that the relationship between rho and the FDR is depending on the model of publication bias or p-hacking. With a pure publication bias dropping 37% of non-significant results (model 1), rho is equal to 22.7% and the FDR is equal to 9.1%. With a p-hacking changing 9.5% of all non-significant tests to significant (model 2), rho is also equal to 22.7%, but the FDR is increased to 20.4%. Eventually, with a p-hacking changing 15.4% of P-values between 0.05 and 0.08 to P-values between 0.04 and 0.05 (model 3), the rho statistic is also equal to 22.7%, but the FDR is only 9.6%. The calculation shown on Figure 5 is incorrect according to all models I have described. The real publication selection bias is probably a mix of all these techniques. Unfortunately, the true FDR cannot be calculated without knowledge of the frequency and techniques of p-hacking and publication bias ; we do not have this knowledge.

I have two proposals to authors to deal with that problem: the first option is to completely remove results related to the FDR. The second option is to provide the two most extreme models and show both results, discussing that the truth is probably between the two extremes. The minimal FDR is found in the model of pure publication bias (9.1%), since it is not increased at all compared to a world where there would be no publication bias at all. The second model I propose is based on p-hacking of some non-significant results to make them become significant. This second model can be parametrized by two distributions: the distribution of probabilities of p-hacking and the distribution of p-hacked values. The distribution of probabilities of p-hacking may have a “peak” for p-values close to 0.05. This peak would greatly increase the rho statistic but would not greatly increase the FDR. Therefore, the pessimistic option, leading to a high estimate of the FDR, is the uniform distribution of probabilities of p-hacking on the range ]0.05 – 1.00]. The distribution of p-hacked values could have a higher density probability in the range [0.04 – 0.05] than in the range [0.00 – 0.04[; that would increase much the rho statistic without increasing that much the FDR. Therefore, the hypothesis of uniform distribution of p-hacked values is rather pessimistic, leading to a high estimate of the FDR. This pessimistic case is the scenario is the “model 2” I described above. This is not exactly the “worst” case, but it is quite pessimistic. The “worst” case would be a massive p-hacking of large p-values (e.g. p> 0.10) to very small p-hacked p-values (p<0.03). This “worst” case would not increase the rho statistic but would greatly increase the FDR. However, this worst case is probably not realistic. I propose that you show “model 2” as pessimistic case and discuss the fact that the “true model” is unknown.

# Minor concerns and suggestions

## Minor concern 1: clarity of homogeneity assumption

I suggest that authors stress more on the assumption of homogeneous effect size within each broad category in the abstract and manuscript.

For instance, the sentence on line 15-16, "*In sum, the statistical power was very low and showed only a slightly increasing trend over time*" could be changed to "*Assuming the effect size was homogeneous among all tests of each domains of psychology, the statistical power was very low...*"

On line 290-291, after the words "When turning to the primary research outcomes, the retrospective statistical power over the examined years between 1975 and 2017 was low (43.5%, Fig 3)", you could add, ", assuming all effects tested in each subfield were homogeneous".

The limitation is already well acknowledged in the Discussion section.

Of course, you are free to reword that, but I think that this assumption should be very clear at every stage.

## Minor concern 2: model with heterogeneity

Authors answered to the concern about homogeneity assumption:

*« I also agree with this suggestion and integrated a retrospective statistical power estimate that accounts for effect heterogeneity by using the suggested tau² from random-effects meta-analyses. »*

They describe this random-effect meta-analysis in Section 1.2 of Supplementary Information. However, it seems that the effect heterogeneity has been taken in account to get a better estimate of the average effect, which is nice but does not answer to the main concern of a possible covariance between effects and sample sizes.

I do not think that there is a solution to that problem. Acknowledging this limitation in the abstract, methods, results and discussion is all you can do. You can do that by following advice I gave in “minor concern 1”. Moreover, I suggest that you add the words “*in the average effect estimate*” after “*for a retrospective statistical power accounting for effect heterogeneity*” on line 170-171 to clarify the heterogeneity problem it solves.

## Minor concern 3 : answer to authors comment (no manuscript modification required)

I wrote:

« Another problem exists. Not all tested hypotheses are true. If half of the hypotheses are true, the

pooled effect size would be diluted by a factor two, ie. grossly underestimated. Therefore, the

pooled effect size must have a correction factor, based on the hypothetical rate of true hypotheses,

which is unknown. »

Authors responded:

*« I somewhat struggled with this suggestion. Although the underlying effect is non-zero, a research*

*hypothesis still can be wrong (e.g. in a different population, using slightly different methodology) – in*

*short, all factors that contribute to the between-study heterogeneity. As standard errors should not*

*differ between true and false hypotheses, the only reason for an underestimated power would be, as*

*you correctly note, a underestimated true effect. Given the on average the estimated mean effects*

*are around d~0.5, they are already seem quite large. Assuming just a priori a e.g. small effect, in my*

*opinion, makes things worse because available (although diluted and noisy) information is discarded.*

*The concept of retrospective power analyses is also used e.g. in economics (Ioannidis JPA, Stanley TD,*

*Doucouliagos H. 2017). »*

To clarify my position. The problem is about the estimator of the average effect size.

You estimated that the mean effect size is 0.499. If half of the hypotheses tested are true, that value 0.499 could actually be the average between a Cohen's d equal to 0.998 (H1) for half of the hypotheses tested and 0.000 (H0) for the other half. Indeed 0.499 = 0.998*0.50+0.000*0.50

The "statistical power" would not be poor for the tests with a Cohen's d equal to 0.998 and would not be applicable to tests with an effect equal to 0.000 (only the type I error rate can be defined for zero effects).

From another point of view, the average effect size should be computed from the set subset of tests that have been performed on true hypotheses (H1) rather than wrong hypotheses (H0).

Unfortunately, I see no way of knowning which hypotheses are true or wrong. The average effect could be multiplied by the inverse of the proportion of true hypotheses (e.g. 0.50) to correct this bias, but this proportion is not really known.

Your approach of defining a priori effects (d=0.2, d=0.5, etc.) in a secondary analysis, workarounds that problem. So I think you did the best you can. I do not call for a modification in the manuscript for that.

## Minor concern 4: writing style

On line 60 (legend of figure 1), I suggest changing *“allows quantifying the FP share on all diagnosed effects (TP + FP)"* to *" allows quantifying the FP share on all positive statistical tests (TP + FP)*" to avoid any suggestion of a medical diagnosis.

On line 131, I suggest changing “*because this information could not be retrieved from the test values*” to “*because it was not possible to automatically detect whether tests were paired or not*" to clarify the type of information.

On line 133, I suggest changing “*have to be assumed*” to “*had to be assumed*”.

On line 141, I suggest changing “*because for z, no information*” to “*but not for z, because no information*”

On line 165, phi should be described as being the cumulative distribution function of the standard normal distribution.

On line 179, change “*calipüers*” to “*calipers*”.

On line 268, change “*96 Mio*” to “*96 millions*”.

On line 274, close the parenthesis opened on line 273.

On line 287, I suggest changing “*The number of observations is with*” to “*The sample size is*”.

On line 288, change “*270 Mio*” to “*270 millions*”.

## Minor concern 5

On lines 312-314, the sentence "*The replication rate based on the retrospective statistical power estimate is only slightly larger (44.8%) than the average retrospective statistical power*" looks wrong to me.

Indeed, the replication rate (44.8%) is slightly **smaller** than the average retrospective statistical power (49.9%).

## Minor concern 6: extrema

On line 246, the extrema of effect sizes are shown (d=0.002 and d=1.206). Similarly, many extrema are shown on table 1. Extrema are not much informative on PIC areas, because there are many PIC areas with few articles, with very poor estimates of the average effect. Extrema about articles and test values on table 1 are clearly outliers. I suggest that you add the 1^st^ and 3^rd^ quartiles. If you are interested in less typical values, you may add the 1^st^ and 9^th^ deciles. Extrema should only be kept if you have enough space in the table, but are not of major interest.

I guess that authors checked that extrema are not due to automatic extraction errors. For instance, the largest sample size, 96 millions, has probably been manually checked. If that is true, authors are invited to specify that they checked these outliers manually and they were correct. Otherwise, a reader may suspect that these are errors.

## Minor concern 7: clarity of table 1

Although table 1 contains much useful information I struggled reading this table. First, commas should be used as thousands separator and dots as digits separator. For instance, the number “5073.153” can be written “5,073.153” but should not be written “5.073.153”.

The column header “Observations” should be renamed to “Number of observations”. The first column is currently unnamed. It could be named “Type of observation”.

Row names should be more explicit. In the “Meta-analyses of PIC areas” section, “Articles” could be renamed to “No articles per PIC” and “Test values” could be renamed to “No test values per PIC”. In the “Articles” section, “Mean nr. of observations” could be renamed to “sample size for the tests”. In the “Test values” section, “N” could be renamed to “Sample size for the test”.

## Minor concern 8: clarity of figure 2

The flow chart contains both the number of tests and number of articles. This is very nice but the abbreviations (k = article, n = tests) are not very well introduced. I propose the following modifications: in the second box, replace “Excluded (k = 11,690)” by “Excluded (k = 11,690 articles)”. In the third box, replace “k = 42,170; testvalues (n): 734,748” by “k = 42,170 articles; n = 734,748 test values”.

This is only a suggestion. If the editor has a better idea, follow his advice.
