# Supplementary material for: Are most published research findings false? Trends in statistical power, publication selection bias, and the false discovery rate in psychology (1975–2017)
Source: PLoS One. 2023 Oct 17;18(10):e0292717. doi: 10.1371/journal.pone.0292717 (PMC10581498; doi:10.1371/journal.pone.0292717)
Supplement: Attachment — Submitted filename: response_rev.docx [file pone.0292717.s007.docx]

Dear Dr. André Gillibert,

Thanks again for your constructive and helpful comments that substantially improved the paper. Below you find my point-by-point response to your remarks; the newly introduced text is highlighted in blue, and my reply in green.

I hope that the manuscript is now suitable for publication in PLOS ONE.

Again many thanks for your remarks and best wishes

Andreas Schneck Dear André Gillibert,

Thanks again for your constructive and helpful comments that substantially improved the paper. Below you find my point-by-point response to your remarks; the newly introduced text is highlighted in blue, and my reply in green.

I hope that the manuscript is now suitable for publication in PLOS ONE.

Again many thanks for your remarks and best wishes

Andreas Schneck

I completely agree with your major point that lumping together publication bias and p-hacking is not applicable regarding the consequences of both on the FDR. I changed the calculations accordingly to your code example and included a new section in the main text (below in blue). Furthermore, I added a more technical description of the p-hacking estimator in the supplemental material (p. 7-10).

In contrast to previous studies, the CT in this article estimates the prevalence of publication selection bias rather than serving as a diagnostic test. Although both forms of publication selection bias: publication bias, and p-hacking, can be tested with the CT, they have different properties that must be considered when estimating their prevalence. Publication bias omits nonsignificant results while leaving the number of false-positives unchanged. P-hacking, in contrast, converts nonsignificant results into significant results, increasing the number of false-positives (cp. Fig 1) directly.

The prevalence of publication bias, $pbr$, is directly estimable from the expected significant results and the observed probability of just significant values in the CT. The expected probability of finding a significant result, $E\left( sig \right),$ is a joint measure of the statistical power ($pow$) and the chosen critical *p*-value in this case 0.05 for the 5%-threshold weighted by the assumed prevalence of true effects, $\theta$ denominating how many tested hypotheses are true (1: 2). Because in the examined psychological literature, a strong theoretical tradition exists that increases the *a priori* probability of a true underlying effect (2, 3), θ = 50% was chosen for the main analyses (for a similar 'best case scenario' see 4). To cover also less optimistic scenarios also θ = 1%, 10% & 20% were used (5).

$E\left( sig \right)=\theta*pow+\left( 1-\theta\right)*\alpha$.

The estimated prevalence rate building on the probability of the just significant caliper (over caliper, $OC$) is then:

$pbr=(1-0.5/OC)/(1-E\left( sig \right)$)

In contrast, estimating the prevalence of p-hacking rests on more assumptions as nonsignificant studies are actively made significant by the researcher. Therefore, the probability distribution of the respective hypotheses has to be recovered to estimate the needed p-hacking prevalence to fit the observed probability distribution of the just significant and nonsignificant values in the CT given the estimated statistical power and the critical *p*-value. As this procedure involves multiple equations, a unit root solver was implemented to recover the p-hacking prevalence ($phr$, for further details on the estimation, see section 1.2. in S1 Appendix).

Some assumptions have to be made for both estimators of publication bias and p-hacking. First, publication selection bias is implemented irrespective of the nonsignificant *p*-value achieved at first hand, meaning that a strongly nonsignificant result is equally likely to be treated by publication selection bias. This means that nonsignificant *p*-values are dropped with an equal probability in the case of publication bias (e.g. borderline significant results, 0.051 or far off p = 0.9). This process is also applicable under p-hacking because results can be changed by switching control variables (e.g., controlling on so-called collider variables, 6). Second, p-hacking is always successful in finding a significant specification, as this might be the case of researchers that employ several rounds of p-hacking to obtain a significant result. Third, although the CT does not need distributional assumptions, the prevalence estimates of publication bias and p-hacking are dependent on the assumed prevalence of true effects ($\theta$) and the estimated statistical power.

Both practices of publication selection bias increase the FPR beyond the set false-positive rate (e.g., 5%). For publication bias, because nonsignificant results drop out, the denominator is reduced by the reduced share of nonsignificant true-negative results (cp. Fig 1).

$${FPR}_{inf\_pb}=FPR/(FPR+\left( 1-pbr \right)*(1-FPR))$$

For p-hacking, the inflated false-positive rate can be computed by the sum of the expected false-positive rate and the false-positive rate obtained by p-hacking in the population at risk (nonsignificant results).

$${FPR}_{inf\_phr}= FPR+\left( 1-FPR \right)*phr$$

More informative than the isolated description of the statistical power and the prevalence of publication bias or p-hacking is the joint measure, the FDR. The FDR denominates the share of published significant false results (FPR) on all published significant findings (FPR and statistical power, pow). However, calculating the FDR also requires, as the prevalence of publication bias and p-hacking, an assumption on the prevalence of true effects θ. This assumption is necessary because only the FPR and the statistical power (in their inflated or uninflated form) are estimable rather than the number of false-positives and false-negatives (cp. Fig. 1).

$$FDR=\frac{\left( 1-\theta\right)FPR}{\theta pow+\left( 1-\theta\right)FPR}$$

As p-hacking converts nonsignificant results into significant ones, p-hacking increases the FDR. However, for publication bias, this is not the case, as the false-positives remain unchanged (cp. Fig 1). This can also be shown in the equation above as the dropped nonsignificant results also change $\theta_{pb\_inf}=\theta/(\theta*\left( 2-phr) \right)$. Therefore the FDR with publication bias is equivalent to the unbiased FDR. As it is impossible to specify the relative incidence of p-hacking and publication bias from the data, both estimates form a plausible range of an underlying FDR. Given the fact that p-hacking is a more general framework that reduces authors' costs of producing significant results, p-hacking might be more prevalent.

An additional assumption is the independence of tests, meaning that there are no (or not substantial) exact re-analyses included in the data (studies using the same data and the same or at least largely similar analysis procedure). As even replications are hard to publish in psychology (55), so this should be of no or negligible concern.

Thank you also for your minor concerns, I included the suggested changes in the manuscript, for details see below:

# Minor concerns and suggestions

## Minor concern 1: clarity of homogeneity assumption

I suggest that authors stress more on the assumption of homogeneous effect size within each broad category in the abstract and manuscript.

For instance, the sentence on line 15-16, "*In sum, the statistical power was very low and showed only a slightly increasing trend over time*" could be changed to "*Assuming the effect size was homogeneous among all tests of each domains of psychology, the statistical power was very low...*"

Assuming homogeneous effect sizes within different psychological domains, the statistical power was very low and showed only a slightly increasing trend over time.

On line 290-291, after the words "When turning to the primary research outcomes, the retrospective statistical power over the examined years between 1975 and 2017 was low (43.5%, Fig 3)", you could add, ", assuming all effects tested in each subfield were homogeneous".

When turning to the primary research outcomes, the retrospective statistical power over the examined years between 1975 and 2017 was, under the assumption of effect heterogeneity within the psychological subfields, low (43.5%, Fig 3)

The limitation is already well acknowledged in the Discussion section.

Of course, you are free to reword that, but I think that this assumption should be very clear at every stage.

## Minor concern 2: model with heterogeneity

Authors answered to the concern about homogeneity assumption:

*« I also agree with this suggestion and integrated a retrospective statistical power estimate that accounts for effect heterogeneity by using the suggested tau² from random-effects meta-analyses. »*

They describe this random-effect meta-analysis in Section 1.2 of Supplementary Information. However, it seems that the effect heterogeneity has been taken in account to get a better estimate of the average effect, which is nice but does not answer to the main concern of a possible covariance between effects and sample sizes.

I do not think that there is a solution to that problem. Acknowledging this limitation in the abstract, methods, results and discussion is all you can do. You can do that by following advice I gave in "minor concern 1". Moreover, I suggest that you add the words "*in the average effect estimate*" after "*for a retrospective statistical power accounting for effect heterogeneity*" on line 170-171 to clarify the heterogeneity problem it solves.

Besides the retrospective power, also the results for a retrospective statistical power accounting for effect heterogeneity in the average effect estimate (7, for further details, see section 1.1. in S1 Appendix) and *a priori* specified levels for small (*d* = 0.2), medium (*d* = 0.5) and large effects (*d* = 0.8) were calculated (cp. 8).

## Minor concern 3 : answer to authors comment (no manuscript modification required)

I wrote:

« Another problem exists. Not all tested hypotheses are true. If half of the hypotheses are true, the

pooled effect size would be diluted by a factor two, ie. grossly underestimated. Therefore, the

pooled effect size must have a correction factor, based on the hypothetical rate of true hypotheses,

which is unknown. »

Authors responded:

*« I somewhat struggled with this suggestion. Although the underlying effect is non-zero, a research*

*hypothesis still can be wrong (e.g. in a different population, using slightly different methodology) – in*

*short, all factors that contribute to the between-study heterogeneity. As standard errors should not*

*differ between true and false hypotheses, the only reason for an underestimated power would be, as*

*you correctly note, a underestimated true effect. Given the on average the estimated mean effects*

*are around d~0.5, they are already seem quite large. Assuming just a priori a e.g. small effect, in my*

*opinion, makes things worse because available (although diluted and noisy) information is discarded.*

*The concept of retrospective power analyses is also used e.g. in economics (Ioannidis JPA, Stanley TD,*

*Doucouliagos H. 2017). »*

To clarify my position. The problem is about the estimator of the average effect size.

You estimated that the mean effect size is 0.499. If half of the hypotheses tested are true, that value 0.499 could actually be the average between a Cohen's d equal to 0.998 (H1) for half of the hypotheses tested and 0.000 (H0) for the other half. Indeed 0.499 = 0.998*0.50+0.000*0.50

The "statistical power" would not be poor for the tests with a Cohen's d equal to 0.998 and would not be applicable to tests with an effect equal to 0.000 (only the type I error rate can be defined for zero effects).

From another point of view, the average effect size should be computed from the set subset of tests that have been performed on true hypotheses (H1) rather than wrong hypotheses (H0).

Unfortunately, I see no way of knowning which hypotheses are true or wrong. The average effect could be multiplied by the inverse of the proportion of true hypotheses (e.g. 0.50) to correct this bias, but this proportion is not really known.

Your approach of defining a priori effects (d=0.2, d=0.5, etc.) in a secondary analysis, workarounds that problem. So I think you did the best you can. I do not call for a modification in the manuscript for that.

Thank you for clarifying!

## Minor concern 4: writing style

On line 60 (legend of figure 1), I suggest changing *"allows quantifying the FP share on all diagnosed effects (TP + FP)"* to *" allows quantifying the FP share on all positive statistical tests (TP + FP)*" to avoid any suggestion of a medical diagnosis.

Changed accordingly

On line 131, I suggest changing "*because this information could not be retrieved from the test values*" to "*because it was not possible to automatically detect whether tests were paired or not*" to clarify the type of information.

Changed accordingly

On line 133, I suggest changing "*have to be assumed*" to "*had to be assumed*".

Changed accordingly

On line 141, I suggest changing "*because for z, no information*" to "*but not for z, because no information*"

Changed accordingly

On line 165, phi should be described as being the cumulative distribution function of the standard normal distribution.

Changed accordingly

On line 179, change "*calipüers*" to "*calipers*".

Changed accordingly

On line 268, change "*96 Mio*" to "*96 millions*".

Sentence pruned

On line 274, close the parenthesis opened on line 273.

Changed accordingly

On line 287, I suggest changing "*The number of observations is with*" to "*The sample size is*".

Changed accordingly

On line 288, change "*270 Mio*" to "*270 millions*".

Changed accordingly

## Minor concern 5

On lines 312-314, the sentence "*The replication rate based on the retrospective statistical power estimate is only slightly larger (44.8%) than the average retrospective statistical power*" looks wrong to me.

Indeed, the replication rate (44.8%) is slightly **smaller** than the average retrospective statistical power (49.9%).

I clarified:

The replication rate based on the retrospective statistical power estimate is only slightly larger (44.8%) than the average retrospective statistical power (43.5%)

## Minor concern 6: extrema

On line 246, the extrema of effect sizes are shown (d=0.002 and d=1.206). Similarly, many extrema are shown on table 1. Extrema are not much informative on PIC areas, because there are many PIC areas with few articles, with very poor estimates of the average effect. Extrema about articles and test values on table 1 are clearly outliers. I suggest that you add the 1^st^ and 3^rd^ quartiles. If you are interested in less typical values, you may add the 1^st^ and 9^th^ deciles. Extrema should only be kept if you have enough space in the table, but are not of major interest.

Many thanks, I pruned the extrema from the tables and replaced them with the 1^st^ and 3^rd^ quartile.

I guess that authors checked that extrema are not due to automatic extraction errors. For instance, the largest sample size, 96 millions, has probably been manually checked. If that is true, authors are invited to specify that they checked these outliers manually and they were correct. Otherwise, a reader may suspect that these are errors.

I added the respective information:

(in this case, a manual check revealed: distinct human names from process-produced data)

## Minor concern 7: clarity of table 1

Although table 1 contains much useful information I struggled reading this table. First, commas should be used as thousands separator and dots as digits separator. For instance, the number "5073.153" can be written "5,073.153" but should not be written "5.073.153".

Changed accordingly

The column header "Observations" should be renamed to "Number of observations". The first column is currently unnamed. It could be named "Type of observation".

Changed accordingly

Row names should be more explicit. In the "Meta-analyses of PIC areas" section, "Articles" could be renamed to "No articles per PIC" and "Test values" could be renamed to "No test values per PIC". In the "Articles" section, "Mean nr. of observations" could be renamed to "sample size for the tests". In the "Test values" section, "N" could be renamed to "Sample size for the test".

Changed accordingly

## Minor concern 8: clarity of figure 2

The flow chart contains both the number of tests and number of articles. This is very nice but the abbreviations (k = article, n = tests) are not very well introduced. I propose the following modifications: in the second box, replace “Excluded (k = 11,690)” by “Excluded (k = 11,690 articles)”. In the third box, replace “k = 42,170; testvalues (n): 734,748” by “k = 42,170 articles; n = 734,748 test values”.

This is only a suggestion. If the editor has a better idea, follow his advice.

Changed accordingly
