# Supplementary material for: Are most published research findings false? Trends in statistical power, publication selection bias, and the false discovery rate in psychology (1975–2017)
Source: PLoS One. 2023 Oct 17;18(10):e0292717. doi: 10.1371/journal.pone.0292717 (PMC10581498; doi:10.1371/journal.pone.0292717)
Supplement: Attachment — Submitted filename: response_rev_rev_rev.docx [file pone.0292717.s009.docx]

Dear Dr. André Gillibert,

Thank you again for your constructive and helpful comments that substantially improved the paper. Below you find my point-by-point response to your remarks; the newly introduced text is highlighted in blue, and my reply in green.

I hope that the manuscript is now suitable for publication in PLOS ONE.

Again many thanks for your remarks and best wishes

Andreas Schneck

# General remarks

First, I thank the author to have taken in account all my comments thoroughly and to have deeply reworked the manuscript. The research subject is of great interest and the author did his best to improve the manuscript. Most assumptions underlying the statistical models are acknowledged, so that the reader can understand the limitations. In accordance with my suggestion in the previous reviewing, the author provide robustness analyses under different assumptions to the publication selection mechanisms: either pure p-hacking or pure publication bias. The real mechanism is probably a mix of both. This helps to understand the actual limitations of the study. The manuscript wording can be improved to insist on the fact that both mechanisms (p-hacking and publication bias) were assessed separately, with the assumption that the other mechanism does not exist. One of my concerns is about the necessary statistical complexity of this manuscript. I see no way to simplify that, but I fear that many readers will not grasp all the limitations. Overall, I think that limitations of the study are major and that they should be well acknowledged and accurately reflected in the abstract and conclusion.

I completely agree, I stressed the limitations more thoroughly in the manuscript, especially in the new section central limitation (the missing fit between expected and empirical share of statistically significant findings. Furthermore I added in the abstract:

As the analyses at hand rely on multiple assumptions that cannot be tested on the rather heterogenous set of data also alternative scenarios were laid out, resulting again in a rather optimistic result that although research results suffer from low statistical power and publication selection bias most of the results reported statistically significant may be substantial results rather than statistical artifacts.

My opinion is that it is basically impossible to assess the false discovery rate (FDR) from these data with an acceptable level of reliability, but that it had to be tried; failing to accurately assess the FDR is an interesting result, even if it is “negative”.

I spotted several errors in equations. All of them can be fixed. I have many minor comments and suggestions. I hope that this manuscript will be greatly improved once again if the editor accepts that the author revises it.

I would like to thank the author for the “S2 Replication Package”. This helped me much in the reviewing process. This is a good example of how “open science” can improve the quality of research work. If the manuscript is accepted, I hope that the editor will publish all the reviewing history.

I completely agree and would love to see the reviewing history published as well!

There is much work to do (again)! Good luck!

# Supplementary Information: description of the p-hacking mechanism

Assumptions about the mechanism of p-hacking are described in page 7 and 8 of the Supporting Information: “constant likelihood to engage in publication bias or p-hacking”, “constant statistical power”, and the fact that the probability of a true underlying effect θ has to be known to calculate the p-hacking rate.

When reading equations C and D on page 9, I found another assumption, that was not clearly described: when p-hacking, researchers are assumed to draw a new **independent** P-value, based on the **exact same effect size** as if they repeated the whole experiment with new subjects. If the null hypothesis is true, this effect size is zero; if the alternative hypothesis is true, the standard effect size leading to the general common power, is used. Authors are assumed to repeat this p-hacking process until they get a significant P-value; then, they stop and publish.

I fear that the p-hacking mechanism is often a “fine tuning” of P-values with slight modifications in inclusion/exclusion criteria, adjustment variables, transforming a P-value equal to 0.08 in a P-value equal to 0.04, with a high probability of the final value being in the caliper.

I guess that the step by step P-value generation process should be described in detail, such as below (reformulate if you wish to clarify some things).

The P-value generation process is based on four parameters:

1) θ, the probability that the alternative hypothesis (H1) is true
2) power, the probability of a significant result without any p-hacking or publication biais, if H1 is true
3) α, the significance threshold (5%)
4) phr, the probability of p-hacking (making a non-significant result significant) conditional to the fact that the first P-value analyzed is non-significant

The P-value generation process is described below:

1) Draw a random variable with Bernoulli distribution to define whether H0 or H1 is true, with probability θ of making H1 true
2) If H1 is true, define ar=power, otherwise ar=α
3) Define $th=\Phi^{-1}\left( 1-\frac{\alpha}{2} \right)$ the significance threshold of a z-value in a normal-based two-sided statistical test ($th=1.96$ for $\alpha=5\%$)
4) Define $Z_{1}\mathcal{\sim N}\left( \mathrm{th}+\Phi^{-1}\left( power \right),1 \right)$ if H1 is true and $Z_{1}\mathcal{\sim N}\left( 0,1 \right)$ if H0 is true, the random variable that represents a z-value of a normal-based statistical test.
5) Draw a random value $z_{1}$ of the variable $Z_{1}$, representing the first test statistic
6) Compute $p_{1}=2\times\Phi\left( -\left| z_{1} \right| \right)$, the first P-value, obtained without any p-hacking
7) Define $i=1$ the number of statistical tests actually performed at this stage.
8) If $p_{1}\leq\alpha$, the algorithm stops and the final P-value generated is $p_{1}$
9) If $p_{1}>\alpha$, draw a random variable with Bernoulli distribution to define whether authors will p-hack, with probability $phr$ of p-hacking
10) If authors do not p-hack (according to step 8), the algorithm stops and the final P-value generated is $p_{1}$
11) If authors p-hack (according to step 8), they will repeatedly execute steps 12 to 15 until a significant P-value is generated.
12) Increment $i$ by one (if $i=1$, it becomes 2, if $i=2$, it becomes 3, etc.)
13) Define $Z_{i}\mathcal{\sim N}\left( \mathrm{th}+\Phi^{-1}\left( power \right),1 \right)$ if H1 is true and $Z_{i}\mathcal{\sim N}\left( 0,1 \right)$ if H0 is true, the random variable that represents a z-value of a normal-based statistical test. $Z_{i}$ is independent of all previous $Z_{j}$ variables where $j\in\left\{ 1,\ldots,i-1 \right\}$. The truth of H1 or H0 is unchanged from step 4, assuming that authors draw a new independent P-value from the same experimental context.
14) Compute $p_{i}=2\times\Phi\left( -\left| z_{i} \right| \right)$, the $i^{th}$ P-value generated
15) If $p_{i}>\alpha$ go to step 12, to repeat the process until a significant P-value is generated
16) If $p_{i}\leq\alpha$, the algorithm stops and the final P-value is $p_{i}$

The following assumptions are performed, in the process of p-hacking described above:

1) The probability that H1 is true is known (probability = θ) among all tests performed in the literature
2) The actual statistical power (if p-hacking did not exist) is constant among all tests of the literature where H1 is true
3) The probability of p-hacking ($phr$) is independent of whether H0 or H1 is true. This makes sense, since nobody knows whether H0 or H1 is true; only the P-value is observed.
4) The probability of p-hacking depends on whether the first P-value is significant or not, but is otherwise independent of whether the P-value is close to be significant or far from being significant.
5) Once authors start to p-hack, they do not stop until they get a significant P-value
6) The p-hacking process is performed by drawing **independent** P-values again and again, until one gets significant. This differs from a process of “tuning” existing P-values to make them smaller, by slightly modifying inclusion/exclusion criteria, adjustment variables or statistical models…
7) There is no publication bias at all, only pure p-hacking

Many thanks for your suggestion, I added a respective graph (Fig A) and described it accordingly (too long paragraph to enter here). In order to make the assumptions as clear as possible I stated them in italics and named them as Assumption A, B, etc…

# Supplementary Information: equations

The appendix shows the equations underlying the p-hacking rate (phr) calculation. On page 8 of Supporting Information, in the “Definitions:” paragraph.

1) “ar = α |power” should be replaced by “ar = α or power, depending on whether the null hypothesis (H0) or alternative hypothesis (H1) is analyzed” or any similar wording making it clear that it is equal to alpha or the power, depending on the context.
2) “z = Φ^-1^(1-power)” should be replaced by “z = Φ^-1^(1-power) or Φ^-1^(1-α/2) depending on whether H0 or H1 is analyzed” or any similar wording.

Changed to:

Conditional share of significant results:	$ar=\alpha\mathrm{OR}power, depending if H0 or H1 is true$

Critical value statistical power:	$z=\phi^{-1}\left( 1-power \right)$ OR $\phi^{-1}(1-\alpha/2)$	$, depending if H0 or H1 is true$

In the next paragraph, “[(th+c), th)” should be replaced by “[th, th+c[“ and “[th, (th-c))” should be replaced by “[th-c,th[“ in order to make the lower boundary of intervals smaller than the upper boundary.

Indeed the intervals were misleading because the underlying statistic is unclear, either z or p, I therefore clarified:

“[(th+c), th)” is replaced by $(th, th+c]$, meaning that $th<z\leq th+c$

“[th, (th-c))” is replaced by $(th-c, th]$meaning that $th-c<z\leq th$

I furthermore clarified :

Equation C shows the conditional probability of a just significant result ($p_{OC}$) on the z-statistic from the significant threshold to the upper bound of the over-caliper $[th, th+c)$. The same logic but in the different direction also applies to the just non-significant results ($p_{UC}$) in the under-caliper $(th-c,th]$ in Equation D.

I reproduce below Equation C (page 9 of Supporting Information):

$$p_{oc}=(\left( 1-\Phi\left( z \right) \right)-\left( 1-\Phi\left( c+z \right) \right)+\left( \Phi\left( z \right)-\Phi\left( 2*-th-c+z \right) \right)*\left( 1+\left( 1-ar \right)*phr/ar \right)$$

I think there are errors in this equation. Parentheses are unbalanced and the term

$\Phi\left( z \right)-\Phi\left( 2*-th-c+z \right)$ should probably be replaced by

$\Phi\left( 2*-th+z \right)-\Phi\left( 2*-th-c+z \right)$.

I propose to rewrite this equation C as:

$$p_{oc}=\left( \left( 1-\Phi\left( z \right) \right)-\left( 1-\Phi\left( c+z \right) \right)+\left( \Phi\left( -2 th+z \right)-\Phi\left( -2 th-c+z \right) \right) \right)\times\left( 1+\frac{\left( 1-ar \right)\times phr}{ar} \right)$$

You are completely right, I changed the equation accordingly and checked the R script where it was correctly specified.

Moreover, another, intermediate equation could make it easier to understand equation C.

$p_{oc}=\Pr\left( th<\left| Z \right|<th+c \right)\times\left( 1+\frac{\left( 1-ar \right)\times phr}{ar} \right)$ where $Z\sim\mathcal{N}\left( z,1 \right)$

Clarified see above.

The term $\Pr\left( th<\left| Z \right|<th+c \right)$ represents the probability of a just-significant result if there is no p-hacking at all, conditional to H0.

There are also parentheses errors in equation D (page 9 of Supporting Information):

It should be written as:

$$p_{uc}=\left( \left( 1-\Phi\left( -c+z \right) \right)-\left( 1-\Phi\left( z \right) \right)+\Phi\left( -2th+c+z \right)-\Phi\left( -2th+z \right) \right)\times\left( 1-phr \right)$$

Thanks for your remark, I changed the equation accordingly.

Equation G is very strange, because OC has two different meanings. It looks like a computer assignation operator rather than a mathematical equality. I propose to change the left-hand side of this equation to CR (caliper ratio). Moreover, for consistency with Equation E and Equation F, OC and UC should be replaced by $P\left( OC \right)$ and $P\left( UC \right)$ on the right-hand side. This new Equation G becomes:

$$CR=\frac{P\left( OC \right)}{P\left( OC \right)+P\left( UC \right)}$$

Thanks for your remark, I changed the text accordingly and now introduce the caliper ratio CR in both, the supplement and the main text.

# Methods & Results

Manuscript, line 189, "and is therefore unsed for the main analyses" should be changed to "and is therefore used for the main analyses".

Manuscript, line 197. Just after “*P-hacking, in contrast, converts nonsignificant results into significant results, increasing the number of false positives (cp. Fig 1) directly.*”, please mention the fact that the P-hacking rate and publication bias rate cannot be estimated simultaneously from the caliper test, because there is only one estimated statistic (the CR) but two unknown parameters (phr and pbr). Please, also mention that, consequently, you calculated each one assuming that the other is zero.

I clarified:

Although the CT can be used to estimate the rate of pure publication bias or p-hacking, it is not possible to identify if publication bias or p-hacking or even a combination of both strategies lead to an overrepresentation of just significant results.

Manuscript, line 198. The sentence starts with *“The prevalence of publication bias"*. I propose to change it to "Assuming that the prevalence of P-hacking is zero, the prevalence of publication bias…”.

Changed to:

Assuming that the prevalence of p-hacking is zero, the prevalence of publication bias…

And :

In contrast, estimating the p-hacking rate given publication bias is zero, rests on more assumptions as nonsignificant studies are actively made significant by researchers (for a detailed description of the process of p-hacking, see Fig A in S1 Appendix).

Manuscript, line 207: please, mention that this $E\left( sig \right)$ expectancy is an expectancy *before* publication bias. Due to publication bias, the actual proportion of significant results will be greater.

This part on E(sig) is pruned as it is no longer neccessary to estimate the publication bias rate.

Manuscript, line 209. The sentence “*The estimated prevalence rate building on the probability of the just significant caliper (over caliper, ....) is then:*” is misleading. The equation on line 210 shows that there pbr is zero if OC=0.5. So, I understood that the value OC is the observed probability of just significant caliper **conditional to the fact that the P-value is within calipers**. Maybe you could introduce the statistics: P(OC) the probability of just-significant result, P(UC), the probability of just-non-significant result and CR=P(OC)/(P(OC)+P(UC)) the probability of just-significant result conditional to the fact that the P-value is within the caliper. You may also mention that, without any publication bias, CR should be equal to 0.50. The use of the name CR rather than OC will reduce the confusion.

That you for your suggestion I changes OC to CR and clarified:

In the case of no publication bias or p-hacking CR is expected to be 0.5 (even just significant and nonsignificant effects) because there should be no discontinuity in the distribution of *p*-values around the rather arbitrary significance threshold.

I do not understand the equation on line 210 of the manuscript. Assuming the publication bias is pure, I would expect the equation to be:

$$pbr=2-\frac{1}{CR}$$

Indeed, assuming the caliper is small enough, before any publication bias, $P\left( OC \right)=P\left( UC \right)=c$, whatever the actual power and $\theta$ are. Assuming that, before any publication, $N$ tests are performed, all the $N\times P\left( OC \right)$ just-significant P-values will be published while only a fraction $\left( 1-pbr \right)$ of the $N\times P\left( UC \right)$ just-non-significant results will be published, leading to a $CR=\frac{Nc}{Nc+Nc\left( 1-pbr \right)}=\frac{1}{1+1\left( 1-pbr \right)}=\frac{1}{2-pbr}$. And so, one can easily deduce that $pbr=2-\frac{1}{CR}$.

Maybe some part of the problem is due to the fact that you did not define clearly what *pbr* is. Is it the proportion of **all** tests that are unpublished, or is only about **non-significant** tests? In the formula above, I used the second definition: proportion among non-significant tests. That would be consistent with the *phr* that you defined in Appendix.

Anyway, even if pbr is defined as the proportion of unpublished tests among all tests that have been performed, the formula would be:

$$pbr=\left( 2-\frac{1}{CR} \right)\times\left( 1-E\left( sig \right) \right)$$

The R script seems to be consistent with the formula given on line 210, which seems to be wrong to me.

I changed the formula in the R script to the formula below. Furthermore I clarified in the manuscript:

$$pbr=\frac{CR-(1-CR)}{CR}=2-\frac{1}{CR}$$

Unless I miss something (which I may, of course), I propose to define the pbr as the proportion of non-significant tests that get unpublished, and use the formula:

$$pbr=2-\frac{1}{CR}$$

This removes any dependency on the statistical power (pow) and proportion of non-zero effects (theta). I did perform very simple simulations ; they seem to confirm that my calculation works.

```
theta=0.5 # proportion of hypotheses that are true
power=0.4 # power for true hypotheses (poor power in this example)
caliper=0.05
alpha=0.05
c=caliper*qnorm(1-alpha/2)
pbr=0.50 # proportion of tests dropped among non-significant tests

th=qnorm(1-alpha/2) # significance threshold
pval0=alpha
pvalu=2*pnorm(th-c,lower.tail=FALSE) # undercaliper P-value threshold
pvalo=2*pnorm(th+c,lower.tail=FALSE) # overcaliper P-value threshold

N=1e7 # number of tests for simulations
true=rbinom(N,1,theta) # which hypotheses are true
z=rnorm(N)+ifelse(true, qnorm(power)+qnorm(1-alpha/2), 0) # z-values of tests
pvalues=2*pnorm(-abs(z)) # pvalues before p-hacking

# pvalues of tests after publication bias
dropped = (pvalues > pval0 & rbinom(N,1,pbr)==1)
ppvalues=pvalues[!dropped]
uc=mean(ppvalues>pval0 & ppvalues<pvalu)
oc=mean(ppvalues>pvalo & ppvalues<pval0)
cr = oc/(uc+oc) # proportion of just-significant p-values among p-values within the caliper

good_pbr_estimator = 2-1/cr
Esig = theta*power+(1-theta)*alpha
bad_pbr_estimator = (1-0.5/cr)/(1-Esig)

# good_pbr_estimator is close to pbr but bad_pbr_estimator is not
c(good_pbr_estimator, bad_pbr_estimator, pbr)
```

I clarified:

Assuming that the p-hacking rate is zero, the publication bias rate, $pbr$, is directly estimable from results of the CT as the ratio of the difference of the probability of just significant results, $CR$, and just nonsignificant, $1-CR$, and $CR$, denoting the rate of dropped nonsignificant results. Simplifying the equation then yields:

$$pbr=\frac{CR-(1-CR)}{CR}=2-\frac{1}{CR}$$

On line 226 after the sentence “[…] several rounds of p-hacking to obtain a significant result.” I suggest that you specify that you also assumed that the mechanism of p-hacking was equivalent to indefinitely draw new independent samples of the study, with the same sample size and effect size (non-zero if H1 is true and zero if H0 is true), until a significant P-value is obtained. If the rate of true hypotheses (theta) is high and the statistical power is good, this p-hacking mechanism will tend to generate many P-values that are close to zero, and fewer just-significant P-values. This can be very different from a mechanism of “fine tuning” of p-values with inclusion/exclusion criteria definitions of outcomes and covariate adjustments, that would lead to many just-significant P-values.

I clarified:

Second, p-hacking is always successful in finding a significant specification, as this might be the case for researchers that employ several rounds of drawing a new independent sample of the same underlying effect to obtain a statistically significant result. This second assumption on the actual form of p-hacking is not exhaustive because fine-tuning in the same sample would be possible, e.g., by excluding outliers, changing measures, or control variables.

The next sentence, about the dependence on theta and power may only apply to the p-hacking rate, assuming that my calculations are correct. This should be updated accordingly, unless I missed something.

I clarified:

Third, although the CT does not need distributional assumptions when calculating the publication bias rate, the p-hacking rate depends on the assumed prevalence of true effects ($\theta$) and the estimated statistical power.

Please add a few words to clarify the fact that the equation shown on line 233 of the manuscript is based on pure publication bias, without any p-hacking. For instance, the previous sentence could be worded:

“For pure publication bias, assuming that there is no p-hacking, because nonsignificant results drop out, the denominator is reduced by the reduced share of nonsignificant true-negative results (cp. Fig 1)."

This is somewhat redundant with the hypotheses that are previously described in the manuscript, but I think that this fact should be regularly recalled to make sure that a reader who would superficially read the manuscript understands this assumption.

Similarly, on line 234, the sentence can be updated:

“For pure p-hacking, assuming that there is no publication bias, […]”

I clarified the point on pure publication bias and p-hacking in multiple occasions in the manuscript.

I checked equations on lines 233, 237; they look correct, but their presentation can be slightly improved. I propose to change them to:

$$FPR_{\inf\_pbr} =\alpha/(\alpha+ \left( 1 - pbr \right)\times(1 - \alpha))$$

$$FPR_{inf\_phr}=\alpha+\left( 1-\alpha\right)\times phr$$

Indeed, the “FPR” that was shown on the right-hand side of these equations was implicitly the FPR before any p-hacking or publication bias was applied, while the “FPR” on the left-hand side was the FPR after p-hacking or publication bias. The use of α makes this fact easier to understand in my opinion. It may be worth mentioning, somewhere in the manuscript, which variables are defined before p-hacking and publication bias occur (α, pow, θ, etc.) and which variables are defined after the p-hacking and publication bias occurs (FDR, FPR, etc.).

I changed the entire notation to the calculation of TP, FP, FN and TN, the respective formula is therefore pruned. Also the multiplication sign is now changed in the entire manuscript.

The end of the sentence on line 240 is awkward, if not misleading:

“[…] all published significant findings (FPR and statistical power, pow).”

I propose to reformulate it:

“[…] all published significant findings (true positives + false positives).”

The sentence is now pruned, before the FDR is defined as:

The FDR estimates the share of false-positive results on all significant results detected by the estimator (first row percentage in Fig 1).

Indeed, as the next sentence shows, the number of published significant findings cannot be calculated from FPR and pow alone; theta is required.

I also propose to reformulate the two next sentences with a wording such as:

“The FDR depends on the FPR, power and prevalence of publication bias or p-hacking, but also depends on the prevalence of true effects (θ). An assumption on the value of θ is necessary […]”

or any equivalent formulation.

Thank you for your remark, as you suggested I changed the notation to define TP, FP, FN and TN, therefore the respective sentences were prunded.

Equation on line 245 would be correct only if the θ is interpreted as the proportion of published results about non-zero effects (TP+FN) among all **published** results (TP+FP+FN+TN) and pow is interpreted as the proportion of published significant tests about non-zero effects (TP) among **published** results about non-zero effects (TP+FN). This definition of θ is suggested on line 249 in the expression $\theta_{pb\_inf}=\theta/(\theta*\left( 2-phr \right))$. Unfortunately, the modification of the pow is not mentioned and the expression of $\theta_{pb\_inf}$ seems to be incorrect. First, it refers to *phr* rather than *pbr*. Second, my calculations show quite a different equations and are consistent with basic simulations contrary to $\theta_{pb\_inf}$.

The following simulations and mathematical equations computed in R show that $\theta_{pb\_inf}$ is incorrect:

```
# simulations and mathematical calculations for
# pure publication bias

theta=0.6 # proportion of hypotheses that are true
power=0.7 # power for true hypotheses (good power in this example)
alpha=0.05
pbr=0.30 # proportion of tests dropped among non-significant tests

N=1e7 # number of tests for simulations
true=as.logical(rbinom(N,1,theta)) # which hypotheses are true
z=rnorm(N)+ifelse(true, qnorm(power)+qnorm(1-alpha/2), 0) # z-values of tests
pvalues=2*pnorm(-abs(z)) # pvalues before p-hacking

# which P-values are dropped by pure publication bias?
dropped = (pvalues > alpha & rbinom(N,1,pbr)==1)

# after publication biais, we get two vectors
# ppvalues = published p-values
# ptrue = published "true" effects (i.e. non-zero effects)
ppvalues=pvalues[!dropped]
ptrue = true[!dropped]

# 2x2 table before applying the publication bias
TP=theta*power		# true positives before publication biais
FP=(1-theta)*alpha	# false positives before publication biais
FN=theta*(1-power)	# false negatives before publication biais
TN=(1-theta)*(1-alpha)	# true negatives before publication biais

# this is consistent with our simulations (ratios are very close to 1)
mean(true & pvalues < alpha) / TP
mean(!true & pvalues < alpha) / FP
mean(true & pvalues > alpha) / FN
mean(!true & pvalues > alpha) / TN

# 2x2 table after publication bias (with a 'b' suffix)
TPb=TP		# true positives after publication bias
FPb=FP		# false positives after publication bias
FNb=FN*(1-pbr)
TNb=TN*(1-pbr)

# Note that, now TPb+FPb+FNb+TNb is not equal to 100%
# because the denominator of these rates is the number of tests performed
# BEFORE publication

N2=TPb+FPb+FNb+TNb # total number of published results

TPc=TPb/N2	# true positive rate among published results
FPc=FPb/N2	# false positive  among published results
FNc=FNb/N2
TNc=TNb/N2

# This is consistent with the simulations
# since the ratios between the observed and theoretical values
# is very close to 1
mean(ptrue & ppvalues < alpha) / TPc
mean(!ptrue & ppvalues < alpha) / FPc
mean(ptrue & ppvalues > alpha) / FNc
mean(!ptrue & ppvalues > alpha) / TNc

# thetab = proportion of true effects among published results
# This is equal to TPc+FNc but can also be expressed with TPb, FNb, etc.
thetab=(TPb+FNb)/(TPb+FPb+FNb+TNb)

mean(ptrue) / thetab # ratio close to 1 (consistent with simulations)

# powb = proportion of significant results published among
# true effects that are published
powb =TPb/(TPb+FNb)

mean((ppvalues<alpha)[ptrue]) / powb # ratio close to 1 (consistent with simulations)

# we expand the formula of thetab2 from
# the expression of TPb, FNb, etc.
thetab2 = (theta*power+theta*(1-power)*(1-pbr))/
	(theta*power + (1-theta)*alpha +
	 theta*(1-power)*(1-pbr) + (1-theta)*(1-alpha)*(1-pbr))

abs(thetab2 - thetab) < 1e-15 # thetab2 is equal to thetab (neglecting rounding errors)

# this can be simplified (Xcas helped to simplify the expression of thetab2...)
thetab3 = ((1-power)*pbr-1)*theta/(((alpha-power)*theta-alpha+1)*pbr-1)
abs(thetab3 - thetab) < 1e-15 # thetab3 is equal to thetab

theta_pb_inf = theta/(theta*(2-pbr)) # from line 249 of the manuscript
theta_pb_inf / thetab # quite different from thetab (ratio is far from 1)
theta_pb_inf / mean(ptrue) # clearly inconsistent with simulations

# We apply the equation of line 245 of the manuscript
# with thetab and powb rather than theta and pow

# we apply equation of line 233 of the manuscript
# to get the false positive rate
FPRb=alpha/(alpha+(1-pbr)*(1-alpha))

mean((ppvalues < alpha)[!ptrue]) / FPRb # consistent with simulations (ratio close to 1)

# we now apply equation of line 245
# with the post-publication theta, power and FPR
FDRb=(1-thetab)*FPRb/(thetab*powb+(1-thetab)*FPRb)

mean((!ptrue)[ppvalues < alpha]) / FDRb # consistent with simulations (ratio close to 1)
```

All those complex conditional and unconditional probabilities are not easy to write in the manuscript, and I propose to simplify it by replacing equation of line 245 by equations related to the four values TP, FP, TN, FN. With pure publication bias and no p-hacking, the four equations are:

$$TP=\theta\times pow$$

$$FP=\left( 1-\theta\right)\alpha$$

$$FN=\theta\left( 1-pow \right)\left( 1-pbr \right)$$

$$TN=\left( 1-\theta\right)\left( 1-\alpha\right)\left( 1-pbr \right)$$

Where these four statistics are expressed as proportions of the hypotheses tested, rather than proportion of published results. These equations could even be introduced before equation of line 233 related to the $FPR$.

Once these four statistics are shown one can easily see that $FDR=\frac{FP}{FP+TP}$ is independent of $pbr$, because $FP$ and $TP$ are independent of $pbr$.

With pure p-hacking, the four equations are:

$$TP=\theta\times\left( pow+\left( 1-pow \right)\times phr \right)$$

$$FP=\left( 1-\theta\right)\times\left( \alpha+\left( 1-\alpha\right)\times phr \right)$$

$$FN=\theta\times\left( 1-pow-\left( 1-pow \right)\times phr \right)$$

$$TN=\left( 1-\theta\right)\times\left( 1-\alpha-\left( 1-\alpha\right)\times phr \right)$$

The denominator of these rates is still the initial proportion of hypotheses tested before any p-hacking occurs; it is also equal to the number of published results, because there is no publication bias; only pure p-hacking. The $FDR$ can be calculated as $FDR=\frac{FP}{FP+TP}$.

I checked empirically that these equations were correct (please find, the R code below):

```
# simulations and mathematical calculations for
# pure p-hacking

theta=0.6 # proportion of hypotheses that are true
power=0.7 # power for true hypotheses (good power in this example)
caliper=0.05
alpha=0.05
phr=0.30 # proportion of tests p-hacked among non-significant tests

N=1e7 # number of tests for simulations
true=as.logical(rbinom(N,1,theta)) # which hypotheses are true
z=rnorm(N)+ifelse(true, qnorm(power)+qnorm(1-alpha/2), 0) # z-values of tests
pvalues=2*pnorm(-abs(z)) # pvalues before p-hacking

# which P-values are p-hacked by pure p-hacking?
phacked = (pvalues > alpha & rbinom(N,1,phr)==1)

# after p-hacking, we get two vectors
# ppvalues = published p-values
# ptrue = published "true" effects (i.e. non-zero effects)
ppvalues=ifelse(phacked, 0, pvalues) # for this simulation we do not care
# on the precise distribution of p-hacked values; WARNING, this is not
# correct if you analyze the caliper test.
ptrue = true # we get the same number of published results

# 2x2 table before applying the p-hacking
TP=theta*power		# true positives before p-hacking
FP=(1-theta)*alpha	# false positives before p-hacking
FN=theta*(1-power)	# false negatives before p-hacking
TN=(1-theta)*(1-alpha)	# true negatives before p-hacking

# this is consistent with our simulations (ratios are very close to 1)
mean(true & pvalues < alpha) / TP
mean(!true & pvalues < alpha) / FP
mean(true & pvalues > alpha) / FN
mean(!true & pvalues > alpha) / TN

# 2x2 table after p-hacking (with a 'b' suffix)
TPb=theta*(power+(1-power)*phr)		# true positives after p-hacking
FPb=(1-theta)*(alpha+(1-alpha)*phr)		# false positives after p-hacking
FNb=theta*(1-power-(1-power)*phr)
TNb=(1-theta)*(1-alpha-(1-alpha)*phr)

# Note that TPb+FPb+FNb+TNb is equal to 100%
# because the number of published results is the number
# of initial tests
TPb+FPb+FNb+TNb - 1 # equal to zero

# This is consistent with the simulations
mean(ptrue & ppvalues < alpha) / TPb
mean(!ptrue & ppvalues < alpha) / FPb
mean(ptrue & ppvalues > alpha) / FNb
mean(!ptrue & ppvalues > alpha) / TNb

FDRb = FPb/(FPb+TPb)

mean((!ptrue)[ppvalues < alpha])/ FDRb # consistent with simulations
```

Again many thanks for your remarks and the R code. I changed the notation as suggested.

On lines 250 and 251, the sentence “As it is impossible to specify the relative incidence of p-hacking and publication bias from the data, both estimates form a plausible range of an underlying FDR.” Could be rephrased to make it clearer. I propose “As it is impossible to assess the relative prevalence of p-hacking and publication bias from the published data, the two extreme scenario of pure p-hacking without publication bias and pure publication bias without p-hacking were analyzed separately; the actual FDR may lie between these two extremes, assuming the simple mechanism of p-hacking and publication bias modeled is not too far from reality”

I changed the sentence to:

As it is impossible to assess the relative occurrence of p-hacking and publication bias from the published data, the two extreme scenarios of pure p-hacking without publication bias and pure publication bias without p-hacking were analyzed separately. The actual FDR may lie between these two extremes, assuming the simple mechanism of p-hacking and publication bias modeled is not too far from reality.

On lines 256-257 the sentence “As even replications are hard to publish in psychology (55), so this should be of no or negligible concern.” is not completely accurate in my opinion. I agree that replications studies are very rare, but replications studies use independent samples, and so provide independent P-values; unless you are referring to correlated hypotheses.

The sentence is now pruned.

This may be a problem if one wants to count only once the “discovery” of the original study and the replication study. Anyway, a “stronger” problem is the fact that there are, on average, 17 P-values per article. Two tests performed in the same article are always correlated; most of the time, the correlation is very small and can be neglected. Sometimes, it is stronger, when two redundant outcomes are compared between the same groups, such as two slightly different psychological scales that basically measure the same psychological parameter. This is probably a minor issue, because it does not necessarily change the FDR. It might interact with p-hacking and publication bias. For instance, authors may publish an article whenever they find at least one “cool” significant result. This mechanism is quite different from the publication bias that you modeled. Actually, the mechanism that you modeled is based on independent “publication” of each P-value, and is a mechanism closer to a *selective reporting bias* rather than a *publication bias*. True *publication bias*, depend on all results of the article and affect the whole article at once. The actual publication bias mechanism may even affect studies with one or more positive findings in the opposite direction that the authors want to find.

I agree that this could be misleading, I nonetheless chose to keep the term publication bias due to its wider distribution in psychology. In the manuscript I clarified:

A bias closely related to publication bias is selective reporting bias, where significant results within one dataset are reported, whereas nonsignificant results remain unpublished. Because publication bias and selective reporting bias cannot be distinguished in this study, the more commonly used term publication bias is used.

Overall, I suggest that you briefly mention that the mechanisms of p-hacking and publication bias you defined, rely on the assumption that each test of an article is affected by p-hacking or publication bias completely independently of other tests of the same article. You may also change the term “publication bias” by “selective reporting bias” everywhere in your article; that would probably be more consistent with your methodology.

Many thanks for your remark, I added a sentence on the assumption of independence Not change the term PB, but stress statistical independence:

Another assumption is the independence of tests, meaning that no (or not substantial) exact re-analyses are included in the data (studies using the same data and the same or at least largely similar analysis procedure) and there are no dependencies in the test statistics in one article (e.g. when reporting robustness checks).

On lines 273-274, the author specify that “*p-values of either exact 0 or 1*” are excluded. The flow chart shows 2780 exclusions for that exclusion criteria. This is inappropriate in my opinion. P-values have been recomputed from various statistics (F, Chi-square, etc.). Due to the finite numerical precision of floating point values, R will round to zero any P-value < 5×10^-324; this is called “floating point underflow”. This rounding may be problematic for some of your calculations, but that is a pure technical obstacle and not a relevant exclusion criteria since excluding extremely-significant results would underestimate the statistical power. I propose to compute log(P-value) rather than P-value. Base R functions, such as pnorm, pf, pchisq and pt have an optional parameter log.p=TRUE to return the logarithm of the probability to avoid floating point underflow. The lower.tail=FALSE parameter may also be useful to avoid the need of computing 1-p which would create an underflow at 2.2×10^-16. Another, simple but less accurate solution would be to replace P-values that have been underflowed to zero, by a very small P-value (e.g. 1e-322). Extremely small P-values might have a too big impact on the meta-analysis. If that is the case, some adequate handling may be performed, such as winsorizing; the method and rationale should be described. The truncation of P-values < 5×10^-324 seems to be accidental rather than a choice of truncation of extreme values.

Excluding P-values exactly equal to 1 would overestimate the statistical power. These P-values are possible for two main reasons:

1) Perfect balance between groups, which is not uncommon on binary variables with chi-square tests, such as 5/10 vs 20/40.
2) Rounding a test statistic close to zero (e.g. t=0.004) to exactly zero (t=0.00)

The P-value is correct or almost correct in both cases and I see no obvious reason to exclude them. Maybe there is a problem with the folded-normal distribution estimator when a z-value is exactly equal to zero? If there is a problem, is it possible to winsorize the P-value, i.e. replace P-value that are equal to 1 or too close to 1, by P-values that are slightly below 1 (e.g. 0.9999).

Thanks for the remark, I imputed the smallest and largest possible p-values to avoid any missing data.

In table 1, the “Sample size for the tests” in the “Articles” should be rewritten “Mean sample size for the tests”. This can be understood on line 310… Otherwise, table 1 has been very much improved since last version.

Changed accordingly.

On lines 337-339, I understand that the sentence “*This result also remains similar when computing the retrospective statistical power correcting for the large effect heterogeneity and using an a priori effect size of a medium effect (d = 0.5).*” refers to a model assuming that there are some large effect heterogeneity but the effect size is independent of the sample size. This should be well specified, with words such as “*assuming that the effect size is heterogeneous but independent of the sample size…*”. This hypothesis may be wrong. Indeed, the proportion of significant results among all published tests is not much dependent on the sample size. From your dataset I found, 65% for tiny samples (n< 10), 71% for samples with 10 to 20 observations, 73% for 20 to 50, 74% for 50 to 100, 77% for 100 to 500, 81% for 500 to 5000 and 81% for sample sizes greater than 5000. This may be indicative of two mechanisms:

1) A p-hacking rate or publication bias much higher for small sample sizes than for greater sample sizes
2) Effect sizes much greater for small sample sizes than for greater sample sizes

In the new version of the article I rely on the statistical power based a priori defined effect sizes, they are by definition assume effect homogeneity over time and subfield and of cource no dependency with sample size.

*I added in the main text:*

Third, statistical power estimate assumes a constant underlying effect size across psychology with no dependency of sample size and underlying true effect size

*I added in the supplemental materials:*

*The underlying true effect size is constant across psychology (e.g. sub-fields and also sample sizes)*

Assumption A

As you suggested I also analysed several significance thresholds (10,5,1,0.1%) for deciles of the sample size, here I would argue that 1) may be the main problem.

Subgroup analyses by sample size may be interesting.

On lines 357-358, after “can be repeatedly found in a similar setting is estimated” I propose that you add the words “assuming that neither the original, nor the replication study uses p-hacking or publication bias” unless, of course, you took in account p-hacking and publication bias in the original study or in the replication study. I guess that the “replication rate” is calculated for all significant P-values, including false positives; therefore, I guess that it depends on θ. This should be specified in the sentence describing this result.

I pruned the so-called replication rate as it is not in the center of the results. However, you are completely right the replication rate in the study was defined as the statistical power for statistically significant studies (p < 0.05).

On line 366, please reformulate “The overall prevalence of publication bias”, as “Assuming that there would be no p-hacking at all, the prevalence of pure publication bias would be…” or any equivalent wording that make this assumption clear enough.

I added the respective part ”assuming no publication bias | p-hacking” in any occasion.

On line 369, you may reformulate “In the alternative situation of p-hacking, 10.1% of the results have to be turned significant[…]” as “In the alternative situation of pure p-hacking without any publication bias, 10.1% of the results would been turned significant[…]” or any equivalent wording that makes this assumption clear.

Similarly, you may add reminders in the next sentence:

“The estimated publication selection bias was slightly larger in the estimate based on the 10%-caliper (27.9% for pure publication bias without p-hacking, 13.9% for pure p-hacking without publication bias) and considerably smaller in the 1%-caliper (15% for pure publication bias, 4.7% for pure p-hacking)."

I clarified:

The estimated publication selection bias was slightly larger based on the 10%-caliper (42.5% for pure publication bias without p-hacking, 13.3% for pure p-hacking without publication bias) and considerably smaller in the 1%-caliper (22.8% for publication bias, 4.9% for p-hacking, again in pure strategies). The narrowest 1%-caliper, however, was very volatile over the examined years, which may be a result of the smaller sample sizes in this narrowest caliper.

On line 375, “As a result of p-hacking, instead of the *a priori* set FPR, 5% […]" may be reformulated as “Assuming pure p-hacking without publication bias, instead of the *a priori* set FPR, α = 5%, the FPR would nearly triple to 14.6%”

Changed to:

Under pure p-hacking, without publication bias, the FPR would more than triple to 16% instead of the a priori set FPR, α = 5%. The FPR in the case of publication bias without p-hacking was 7.7%.

On lines 391-392: “In total, in the case of p-hacking” may be changed to “Assuming pure p-hacking without publication bias”

Changed to:

Assuming pure p-hacking without publication bias, up to one-fifth (17.8%) of the significant effects would be false and wrongly detected, meaning those findings would be just statistical artifacts.

On line 398-399: “*[…]the FDR is not fixed to 5% but heavily depends on the statistical power, which is, in our case, low.*"; please mention that it also heavily depends on θ, the proportion of true results.

The sentence is now pruned for better readability.

Did you make sur that θ is taken in account in the calculation of the average effect size and statistical power? Indeed, the statistical power is the probability of getting a significant result **if the effect is a true non-zero effect**. If θ = 50% of tested hypotheses are wrong (actual effect is zero) and (1-θ) hypotheses are true with actual effect size *d*, then, without p-hacking and publication bias the observed average effect size will be θ×*d*. The statistical power should be calculated for the effect size *d*. The FDR will depend on θ, statistical power and phr.

Thank you for your remark, in order to reduce the complexity of the results, I decided to prune the so-called retrospective power estimates that build on the results of sub-field specific meta-analyses. I now believe that the added value of rooting the power estimates empirically in the literature is outweighed by the problems of estimating meta-analyses in folded distributions and, what you mentioned unknown thetas. I now rely entirely on apriori power analyses.

Lines 447-448: the sentence “*In the case of t-tests, the lower statistical power (around -10 percentage points) can be caused by the assumption that always two-sided tests were used*” is correct but misses a part of the problem: hypotheses tested by r tests are quite different from hypotheses tested by t tests. For example, r tests may be used to assess the correlation between psychometric scales, expected to be moderately or highly correlated, while t tests are used to compare groups having had different interventions, with small or very small effect sizes. Moreover, the proportion θ of true hypotheses may be higher for r tests than for t tests. Sample sizes may also differ between studies performing r tests and studies performing other tests. There are many possible explanations to the difference of power between t-tests and r-tests; I do not know which ones are right or wrong.

You may either remove the sentence (which looks like a discussion rather than results) or change it to be more open to alternative explanations, such as: “*In the case of t-tests, the lower statistical power (around -10 percentage points) might be caused by the assumption that always two-sided tests were used, or by differences in prevalence of true hypotheses (theta), effect sizes or sample sizes”*.

The sentence is now pruned.

# Table & figures

On lines 380-382 (legend of fig 4), please specify that each publication selection bias mechanism is calculated with the assumption that the other does not exist.

For example you may add the underlined words:

“*Estimated publication selection bias prevalence over time for different calipers (measures). Panel A shows the result for pure publication bias, with the hypothesis that there is no p-hacking, and panel B for pure p-hacking with the hypothesis that there is no publication bias. It was technically impossible to model both p-hacking and publication bias at once. The dot size is determined by the number of studies (k) in the respective study year.*”

I added with small changes:

*Estimated publication selection bias rate over time for different calipers (measures). Panel A shows the result assuming pure publication bias and no p-hacking, and panel B for pure p-hacking with no publication bias. It was technically impossible to model both p-hacking and publication bias at once. The dot size is determined by the number of studies (k) in the respective study year.*

Similarly one lines 406-409 (legend of fig 5), you may clarify all the hypotheses performed, such as:

*“**Estimated false discovery rate (FDR) over time, either assuming that p-hacking is pure, with the hypothesis of no publication bias, or assuming that the publication bias is pure, with no p-hacking, or assuming that there is no p-hacking and no publication bias at all. For pure p-hacking or pure publication bias, the FDR is calculated from the caliper ratio (CR) based on a 5%-caliper*. *The statistical power calculation was based on the empirical effect size (retrospective meta-analysis approach). The proportion of tested hypotheses that are true (non-zero effects) was arbitrarily assumed to be θ = 50%. The FDR is mathematically identical is there is some pure publication bias or if there is no publication selection bias at all. For robustness for different measures, see Fig 7. The dot size is determined by the number of studies (k) in the respective study year.”*

*I added with small changes:*

*Estimated false discovery rate (FDR) over time, either assuming pure p-hacking and no publication bias or pure publication bias and no p-hacking. For pure p-hacking or pure publication bias, the FDR is calculated from the caliper ratio (CR) based on a 5%-caliper. The statistical power calculation was based on the an a priori assumed medium effect size (d = 0.5). The proportion of tested hypotheses that are true (non-zero effects) was arbitrarily assumed to be θ = 50%. The FDR is mathematically identical if there is some pure publication bias or if there is no publication selection bias at all. For robustness for different measures, see Fig 7. The number of studies (k) in the respective study year determines the dot size.*

The legend of figure 6 is too light. I propose to extend it:

“*Fig 6 FDR robustness plot over different a priori probabilities of true underlying hypotheses, ~~statistical power~~ effect sizes, and p-hacking rates*

*False discovery rate over all possible ~~publication selection bias values~~p-hacking rates (0-100%) including empirical estimators of the p-hacking rate based on different calipers (1%, 5%, 10%) and different effect sizes (d=0.2, d=0.8 and d=0.499, the empirical effect size) and for various theoretical proportions of tested hypotheses that are true (θ = 10, 20, 50%). The publication bias prevalence is assumed to be zero* *for the calculation of the p-hacking rate*”

*I added with small changes:*

*False discovery rate over all possible p-hacking rates (0-100%) including empirical estimators of the p-hacking rate based on different calipers (1%, 5%, 10%) and different effect sizes (d=0.2, d=0.5 and d=0.8) and for various theoretical proportions of tested hypotheses that are true (θ = 10, 20, 50%). The publication bias rate is assumed to be zero for the calculation of the p-hacking rate.*

On the figure 6 the term “power estimates” seems to be used improperly, since Cohen’s d value are shown… This should be called “effect size”. The x-axis label, “p-hacking prevalence” could be renamed to “p-hacking rate” for consistency. I prefer the term “rate” to “prevalence”, because I would expect the denominator of the “prevalence” to be all **published results**, while the denominator is actually the share of initially non-significant results. In order to clarify the fact that the “retrospective” effect size is an empirical estimation in the data set, I propose to change two things. Call that “empirical” rather than “retrospective”, and do not round it to 0.5. The value 0.499 is perfectly nice.

I changed p-hacking prevalence to rate. I also agree that the legend for the statistical power estimates was misleading. As the statistical power estimates based on the a priori true effects are ment, I changed the legend to “stat. power (a priori true eff.)”

As the retrospective statistical power as well as its model for effect heterogeneity were pruned, I now just rely on the a priori chosen values. 0.5 in this context ment the a priori assumed threshold for medium effects (d=0.5), which was nearly identical with the retrospective statistical power estimates.

# Inconsistency between results

One interesting statistic can be analyzed: the positivity rate (PR) defined as the proportion of significant results among all published results.

I think that the observed PR should be compared to the theoretical PR according to θ, pow, α, pbr or phr.

From the R database with n=690,983 test values, the observed PR is equal to 74.1%.

The theoretical PR is equal to (TP+FP)/(TP+FP+TN+FN).

For pure publication bias without p-hacking, the equations are:

$$TP=\theta\times pow$$

$$FP=\left( 1-\theta\right)\alpha$$

$$FN=\theta\left( 1-pow \right)\left( 1-pbr \right)$$

$$TN=\left( 1-\theta\right)\left( 1-\alpha\right)\left( 1-pbr \right)$$

From the manuscript, I could extract that overall pow=0.435, θ=0.50, pbr=0.245, α=0.05

Therefore, the theoretical PR is 39.0% (R code below):

```
PR_from_pbr=function(theta, pow, alpha, pbr) {
	TP=theta*pow
	FP=(1-theta)*alpha
	FN=alpha*(1-pow)*(1-pbr)
	TN=(1-theta)*(1-alpha)*(1-pbr)
	(TP+FP)/(TP+FP+TN+FN)
}
PR_from_pbr(theta=0.50, pow=0.435, pbr=0.245, alpha=0.05)
```

For pure p-hacking without publication bias, the equations are:

$$TP=\theta\times\left( pow+\left( 1-pow \right)\times phr \right)$$

$$FP=\left( 1-\theta\right)\times\left( \alpha+\left( 1-\alpha\right)\times phr \right)$$

$$FN=\theta\times\left( 1-pow-\left( 1-pow \right)\times phr \right)$$

$$TN=\left( 1-\theta\right)\times\left( 1-\alpha-\left( 1-\alpha\right)\times phr \right)$$

Since TP+FP+FN+TN=1 because the number of published results is equal to the tested hypotheses, this can be simplified to PR=TP+FP. From the manuscript, I could extract theta=0.50, pow=0.435, alpha=0.05 and phr=0.101. The R code show how to compute the theoretical PR:

```
PR_from_phr=function(theta, pow, alpha, phr) {
	TP=theta*(pow+(1-pow)*phr)
	FP=(1-theta)*(alpha+(1-alpha)*phr)
	(TP+FP)
}
PR_from_phr(theta=0.50, pow=0.435, alpha=0.05, phr=0.101)
```

This yields a PR=31.9%

Therefore, I could get three different PR:

1) Observed PR = 74.1%
2) Theoretical PR for pure publication bias without p-hacking = 39.0%
3) Theoretical PR for pure p-hacking without publication bias = 31.9%

This discrepancy suggests that one or several hypotheses performed in your manuscript are wrong, such as:

1) Absence of correlation between sample size and effect size. This assumption can be removed since the correlation can be estimated from the data set (Spearman’s R between N and d = −0.477). This could be fixed by meta-analyzing by sample sizes intervals (e.g. quintiles of sample sizes), then, assuming that the effect size is homogenous in a sample size strata.

I clarified :

Third, statistical power estimate assumes a constant underlying effect size across psychology with no dependency of sample size and underlying true effect size. This unobserved heterogeneity may lead to the problem that hypotheses (e.g., with a large underlying true effect) were deliberately conducted with small, adequately powered samples but may be classified as underpowered.

2) Erroneous calculation of the average effect size if the proportion of true hypotheses θ has not been taken in account. The correction factor is simple: just divide the average effect size of all hypotheses tested by θ, to get the average effect size of true hypotheses.

I pruned the retrospective statistical power estimates, still the assumed average effects could be wrong too.

3) Wrong hypothesis on the value of θ

I clarified:

First, the parameter with the largest leverage on the FDR, the share of true hypotheses, is not estimable and remains open for speculation.

4) Wrong hypotheses about the mechanism of p-hacking and publication bias, such as the fact that all non-significant P-values are equally affected by the mechanism, that all tests are independent

I clarified:

Fifth, the assumptions on the underlying process of publication bias and p-hacking have to be valid, this may be especially problematic for p-hacking because a large number of assumptions have to be made (for details, see section 1.2. in S1 Appendix).

The robustness analyses of the manuscript are all “successful”, but the consistency check that I propose (observed vs theoretical PR) is a major failure in my opinion. It should be shown in the manuscript and discussed.

Unless I missed it, the hypothesis #1 (absence of correlation between sample size and effect size) was not clearly stated in the manuscript. This hypothesis should be stated or removed by an appropriate analysis.

Integrated in manuscript and supplemental information, see above.

The observed PR (74.1%) is high. I find two main hypotheses to this high PR:

1) A high statistical power (pow) and high proportion of true hypotheses (θ). Without publication bias or p-hacking, PR=θ×pow+α×(1-θ). A PR=74% can be obtained with θ=75% and pow=90%.
2) Massive p-hacking or massive publication bias. A PR=74% can be obtained with θ=50%, pow=50%, and a pbr=81% (pure publication bias); it can also be obtained with θ=50%, pow=50% and phr=64% (pure p-hacking).

Any intermediate scenario between these two extreme hypotheses is possible.

Anyway, this inconsistency should be shown and discussed, unless it is solved. Even if it is solved, the consistency between the theoretical and observed PR should be shown.

One possible solution to this inconsistency would be to model the two statistics PR and CR, from θ, pow, α, and pbr or phr. Assuming that the p-hacking and publication bias mechanisms are modeled separately, as you already did, there are three unknown parameters and two known statistics. I expect that, if one parameter is chosen (e.g θ=80%), all other parameters (e.g. pow and pbr) can be deduced from PR and CR. Not all choices are possible (e.g. θ=20% is incompatible with the CR and PR you observed). The most extreme choices, compatible with the data set, could be used as robustness analyses. That approach would bypass the calculation of the average effect size, concentrating on the power. Maybe some more complex models are possible, taking in account the proportion of P-values less than 0.005 (see the part “Suggestions”).

Thank you for your very helpful suggestion, I wrote a new section called central limitations in the manuscript and discussed some exemplary scenarios that may lead to the observed share of significant results. A graphical visualization can also be found in the supplemental information.

# Suggestions

A few descriptive statistics may help to better understand the structure of the data set and interpret other statistics. First, the histogram of P-values would be nice to show. A zoom on the critical zone (e.g. 0.02 to 0.08) in a subpanel may help to visualize the CR. The CR, defined as P(OC)/(P(OC)+P(UC)) should also be provided.

Now included in the manuscript (Fig 3).

The crude proportion of P-values less than 0.05 (positivity rate) should be provided overall and by sample sizes subgroups. Acceptable sample sizes groups would be <10, 10-20, 20-50, 50-100, 100-500, 500-1000, 1000-5000, > 5000 or power-of-two groups such as <16, 16-32, 32-64, 64-128, …, 1024-2048, > 2048, or deciles of the sample size. I propose to show a table, with, in rows, the sample size, and in columns, the crude proportion of P-values less than 0.05, 0.01, 0.005 and 0.0001. The CR could also be shown in the same table.

Table added in supplemental materials (Table B).

In the manuscript:

The presence of publication selection bias could also be seen when looking at different significant thresholds for deciles of the underlying number of observations. For deciles with a smaller number of observations statistically significant at the 5% level are much more common than for lower thresholds (e.g. 1% and 0.1%), especially when compared with deciles with a larger number of observations (for details, see Table B in S1 Appendix).

In the supplemental observation:

As shown in the article, the results of the caliper test, as well as the observed *p*-value distribution, provide evidence for publication selection bias. This pattern is also visible when looking at the share of significant test values at the 10%, 5%, 1% and 0.1% significance thresholds computed separately for each decile of the underlying the number of observations (Table B). Over the deciles, the share of statistically significant test statistics drops with a decreasing significance threshold, as expected, whereas around 80% of the test statistics are significant at the 10% significance threshold. The difference between the deciles increased the smaller the significance threshold was set. Deciles with a lower number of observations show substantially lower shares of significant values than larger ones. In the most extreme case, the 0.01% significance threshold, in the smallest decile, 13.1%, and in the largest 44.7% of the test values were statistically significant. This result shows a larger share of just significant results in studies with a lower number of observations that are more prone to publication selection bias practices (for a similar logic, see p-curve & p-uniform 16).

Let us call PRx(threshold) the proportion of P-values less than the threshold. Therefore PR=PRx(0.05).

Indeed, these different values help to interpret the risk of p-hacking.

Without any p-hacking or publication bias, for a positivity rate of 80%, different hypotheses are possible:

1) θ = 100% and pow=80% → that would lead to a PR=80% and a PRx(0.005)=49,8%; this calculated as pnorm(qnorm(0.80)+qnorm(1-0.05/2)-qnorm(1-0.005/2))
2) θ = 78,9% and pow=100% → that would lead to a PR=80% and a PRx(0.005)=79,1%
3) Other intermediate θ/pow combinations, leading to a PRx(0.005)>49,8% and PRx(0.005)<80%

Therefore, one expects to get PRx(0.005) > 49,8%. A PRx(0.005) less than 49,8%, is suggestive of p-hacking and publication bias. Put in other words, a whole literature with most P-values between 0.01 and 0.05 but few “very significant” P-values less than 0.001 is suggestive of publication bias or p-hacking.

I wrote a small R code showing all possible combinations of θ and pow for a given PR, assuming that there are no p-hacking/publication bias:

```
# PR = alpha*(1-theta) + pow*theta is the positivity rate
# compute PRx(alphax), the proportion of significant results with p < alphax
PRx=function(theta, pow, alpha=0.05, alphax=0.005) { # positivity rate at threshold alphax
	pnorm(qnorm(pow)+qnorm(1-alpha/2)-qnorm(1-alphax/2))*theta + alphax*(1-theta)
}
PR_pow2theta=function(PR, pow, alpha=0.05, alphax=0.005) { # no p-hacking/publication bias
	theta = (PR-alpha)/(pow-alpha)
	if (theta < 0 | theta > 1) {stop("impossible theta")}
	c(pow=pow, theta=theta, PR=PRx(theta, pow, alpha, alpha),
          PRx=PRx(theta, pow, alpha, alphax))

}
PR_theta2pow=function(PR, theta, alpha=0.05, alphax=0.005) { # no p-hacking/publication bias
	pow = (PR - alpha*(1-theta))/theta
	if (pow < 0 | pow > 1) {stop("impossible power")}
	c(pow=pow, theta=theta, PR=PRx(theta, pow, alpha, alpha),
         PRx=PRx(theta, pow, alpha, alphax))

}
PR2theta_pow=function(PR, alpha=0.05, alphax=0.005) {
	t(Vectorize(PR_theta2pow)(PR,theta=seq(PR_pow2theta(PR,pow=1)["theta"],1,0.01)))
}

PR2theta_pow(0.80)
```

There is a limitation to this model: it assumes that the distribution of effect sizes is dichotomous, with a peak at zero (probability 1-θ) and a peak at a constant effect size (probability θ) for which the power is pow. However, I think that, conditional to a given PR(0.05), the lowest PRx(0.005) will always be for a distribution of effect size without any variance (i.e. θ=1 and constant power). I did not verify that rigorously (you may do that) but I think that it is related to the non-linearity of pnorm(z-qnorm(1-alphax/2)) as a function of z.

If that is correct, this opens a new way of analyzing p-hacking and publication bias, very different from the caliper test. For instance, I generated that table from your dataset, with the following R code:

ds.long$Nc=cut(ds.long$N, breaks=c(0,10,20,50,100,500,5000,Inf))

alphax=0.005

pr=tapply(ds.long$p<0.05, ds.long$Nc, mean)

prx=tapply(ds.long$p<alphax, ds.long$Nc, mean)

cbind(t(Vectorize(PR_theta2pow)(pr,theta=1,alphax=alphax)),prx)

| Sample size | Observed PRx(0.05) | Lowest theoretically possible PRx(0.005) without p-hacking/publication bias | Observed PRx(0.005) |
| --- | --- | --- | --- |
| ≤ 10 | 65 % | 32 % | 30 % |
| 11-20 | 71 % | 39 % | 41 % |
| 21-50 | 73 % | 40 % | 44 % |
| 51-100 | 74 % | 42 % | 46 % |
| 101-500 | 77 % | 46 % | 52 % |
| 501-5000 | 81 % | 52 % | 63 % |
| ≥ 5001 | 81 % | 52 % | 67 % |

For very small samples (< 20) the observed PRx(0.005) is very close to the worst possible theoretical PRx(0.005). This means that, either the actual effects are very homogeneous, with 100% of true hypotheses and equal effect sizes for all hypotheses, or there is some p-hacking or publication bias.

This is less bad than I thought, but this model is quite optimistic. A less optimistic model could be performed by estimating empirically the heterogeneity of effect sizes and calculating a theoretical PRx(0.005) under an approximate distribution of true effect sizes. This would be performed in relatively homogeneous sample size strata.

Many thanks for your suggestion! I think it is an excellent method to detect publication selection bias. As the main interest of the manuscript lies in the actual estimation of a publication bias rate (with all its limitations), I decided against implementing it in the analyses.

# Discussion & conclusion

Line 460: “*all significant results are just statistical artifacts*". Please, replace the word “are” by “may be”.

Changed accordingly.

There is considerable uncertainty. With an observed positivity rate (PR) at 74.1%, an assuming that the overcaliper is due to pure publication bias (24.5%), the data is compatible with the hypothesis that θ=95%, pow=20%, α=0.05 and pbr=24.5%, leading to an FDR at 1.3%. Assuming that the overcaliper is due to pure p-hacking (10.1%), the data is compatible with the hypothesis that θ=80%, pow=88%, α=0.05 and phr=10.1% leading to an FDR at 3.9%. If the power is quite poor (50%) as you found, θ is poor (e.g. 20%), and there is some p-hacking (phr=10.1%), the FDR would be very high (51.5%); however, that is incompatible with the positivity rate observed on the data set: 74.1% observed vs 22.7% theoretical with those hypotheses.

Lines 467-483 (limitations paragraph): please mention that there are many unverifiable and implausible assumptions on the mechanisms of p-hacking and publication bias. You may also mention that if the p-hacking mechanism is to transform just-insignificant P-values (e.g. within the 5% undercaliper) to just-significant P-values (within the 5% overcaliper), a very minor p-hacking with a negligible impact on the FDR would provide a major change in the CR and would be compatible with your data set.

I clarified:

Fifth, the assumptions on the underlying process of publication bias and p-hacking have to be valid, this may be especially problematic for p-hacking because a large number of assumptions have to be made (for details, see section 1.2. in S1 Appendix).

I furthermore included a detailed discussion of the assumptions in the supplemental information.

For instance, from the data set, I could compute the CR=0.614 which would be compatible with a change of 22.8% of just-insignificant results (0.05 < p < 0.0626) to just-significant results (0.0396 < p < 0.05). I did not perform the exact impact on the FDR but I can tell that this would be grossly equivalent to increasing the significance threshold from 0.05 to 0.05+0.0228×(0.0626-0.05)=0.0503. This would have a very minor impact on the FPR and on the FDR. Actually, I think that this model would provide higher FDR values than the pure publication bias model. Indeed, this mechanism of p-hacking would not explain a high PR contrary to the publication bias. Therefore, the high PR would necessarily come from a high θ and a high statistical power.

See the extended section on the central limitation.

Please, change “*A plausible range of the FDR is, therefore, between the FDR with no bias (publication bias) and its inflated version by p-hacking*” on lines 480-481 to something like “*A plausible range of the FDR is, therefore, between the FDR with no p-hacking (publication bias only) and a large θ (e.g. 90%) and* the *FDR inflated by p-hacking with a very small θ (e.g. 10%)”.* However, this change may become obsolete, depending on how you fix the problems mentioned in the chapter “Inconsistency between results” of my reviewing.

The discussion, from line 491 to 507 contains general advice and opinion about the replication crisis, that are acceptable for a discussion although weakly related to the results. I suggest to stress on the fact that it is a personal opinion by changing a few words. On lines 491-492, “three interventions can be emphasized" could be changed to “**we** propose three interventions”.

Changed accordingly:

Given the low statistical power as well as the substantial inflation due to p-hacking, I propose three interventions that may be of help to decrease the FDR and increase the credibility of science

An explicitly structured conclusion should be added, related to the results & limitations of the study. I propose to separate it from the chapter of the Discussion. The conclusion could contain core messages such as “It is possible that the statistical power is low and FDR is high in the APA journals, but there is considerable uncertainty about these statistics, especially the FDR. Our data is compatible with the most extreme hypotheses (FDR ranging from XX at best to XX at worst). We faced unsolvable modelling limitations that are worth sharing. The knowledge of these limitations is of great interest to anybody who would work on p-hacking and publication bias”

As noted above I introduced an extra chapter in the manuscript, central limitation, that deals explicitly with the missing fit between expected and empirically observed significant thresholds.

I added in the Discussion and conclusion:

Keeping the five limitations and especially the central limitation in mind a wide range of possible FDRs may be compatible with the observed share of significant results. In the most extreme cases the FDR can range, as described in Table 2 from less than 1% to nearly 90%. However, besides the unsolvable modelling limitations of the central parameters of interest, publication selection bias, statistical power and the FDR, the article aimed to root the up to now completely theoretical considerations on the aforementioned parameters at least to some degree empirically.

Of course, I suggest that your conclusion contain those ideas, but not necessarily the specific wording. This is a suggestion.

Unless you fix the inconsistency that I mentioned in the chapter “Inconsistency between results”, I suggest that you a word about it in the conclusion. For instance, “we faced unsolvable modelling limitations with major inconsistencies suggesting that some of our hypotheses were wrong”.

I completely agree, for the respective paragraph, see above.

In my opinion, the conclusion is one of the most important parts of an article. Major limitations can be accepted if the conclusion does not go beyond what can be told from the methods and results. Similarly, the abstract should be very carefully worded so that somebody who only reads the abstraction gets the same message as somebody who gets the whole article and only reads the conclusion.

I changed the abstract and the discussion & conclusion chapter accordingly.
