# Supplementary material for: Are most published research findings false? Trends in statistical power, publication selection bias, and the false discovery rate in psychology (1975–2017)
Source: PLoS One. 2023 Oct 17;18(10):e0292717. doi: 10.1371/journal.pone.0292717 (PMC10581498; doi:10.1371/journal.pone.0292717)
Supplement: Attachment — Submitted filename: Reviewing 2022-12-10.docx [file pone.0292717.s010.docx]

# Introduction

Sorry for this late response. I was quite busy lately.

Thank you for the extensive work you performed to answer to my comments.

Almost all problems in the previous manuscript are fixed. The few remaining problems should not be hard to fix. Although my reviewing is lengthy, I think that the issues it rises are not that that big.

There may be a few substantial modifications that may change some results. This is why I consider that there is a “major revision” although it is less major than previous ones.

# Major comments

## Error in the formula of paired t test?

The statistical power is calculated based on theoretical Cohen’s d (0.2, 0.5 and 0.8), and results show poor power, even for large effect sizes (d = 0.8). This strategy work around many problems that happened when trying to estimate the “true” effect size. The standard error of Cohen’s d is estimated from the published statistic (e.g. t statistic) and sample size and/or degrees of freedom (Table A, section 1.1 of the Supporting Information).

Things are somewhat easier to understand when combining the two formulas of Table A. For instance, the standard error of Cohen’s $d$ for a $t$ statistic is equal to $SE=\frac{\left| \frac{2t}{\sqrt{df}} \right|}{z}=\left| \frac{2}{\sqrt{df}} \right|$ assuming that $z=\left| t \right|$. This is the nice thing with Cohen’s d : the standard error becomes a simple function of the sample size. This is important because it means that the assertion that “statistical power is poor” becomes equivalent to “sample sizes are small”. Of course, that makes sense, but stating that helps to understand how it works.

Lines 127-131 of the manuscript specifies that the conservative assumption that all tests (e.g. t-test) were paired tests was performed, so I expect this calculation to be related to a paired-t test.

However, I fear that this formula is incorrect for a paired $t$ test. Indeed, the number of degrees of freedom is equal to the sample size minus one (≈ sample size). With the assumption that the two values of a pair of values are not correlated, that the between-pairs standard deviation is SD for the first and for the second items of the pair, then, the standard deviation of paired differences (deltas) is $\sqrt{2}\times SD$ and the standard error of the mean of paired differences is $\frac{\sqrt{2}}{\sqrt{df+1}}\times SD$ so that the standard error of Cohen’s d is $\sqrt{\frac{2}{df}}$, which is less than you formula. Assuming that the two values of a pair of values are positively correlated (e.g. R=0.50), the standard error would be even less, when expressed as a Cohen’s d based on the between-pairs standard deviation. For instance, if we note $X_{i}$ as the random variables of the first item of the pair and $Y_{i}$ the random variables of the second item and $VAR\left( X_{i} \right)=VAR\left( Y_{i} \right)=SD^{2}$ and $R=COR\left( X_{i},Y_{i} \right)$ the Pearson’s correlation coefficient between the two items of the pair, then :

$$VAR\left( delta_{i} \right)=VAR\left( X_{i} \right)+VAR\left( Y_{i} \right)-2\times COV\left( X,Y \right)$$

$$=2\times SD^{2}-2\times R\times SD^{2}=SD^{2}\left( 2-2\times R \right)$$

So that:

$$Std.error\left( \mathrm{mean}\left( delta \right) \right)=\sqrt{\frac{SD^{2}\left( 2-2\times R \right)}{N}}=SD\sqrt{\frac{2-2\times R}{N}}$$

So that $Std.error\left( d \right)=\sqrt{\frac{2-2\times R}{N}}$

For $R=0.50$, we get $Std.error\left( d \right)=\frac{1}{\sqrt{N}}\approx\frac{1}{\sqrt{df}}$.

For higher correlations, the standard error is even lower!

Therefore, if t tests are actually paired, you formula probably overestimates the standard error and consequently, underestimates the statistical power, leading to pessimistic conclusions. However, to conclude that there is a problem of poor statistical power, you should rather use optimistic assumptions than pessimistic assumptions.

For an unpaired test, $df=N_{1}+N_{2}-2$. For a given $df$, the standard error of Cohen’s $d$ should be minimal (optimistic scenario) when $N_{1}=N_{2}=\frac{df+2}{2}$. With that assumption $VAR\left( mean diff \right)=\frac{SD^{2}}{N_{1}}+\frac{SD^{2}}{N_{2}}=SD^{2}\left( \frac{1}{N_{1}}+\frac{1}{N_{2}} \right)$ so that $Std.error\left( Cohen^{'}s d \right)=\sqrt{\frac{1}{N_{1}}+\frac{1}{N_{2}}}=\sqrt{\frac{4}{df+2}}=\frac{2}{\sqrt{df+2}}$ which is close to the formula of Table A.

Overall, for the same degrees of freedom, an unpaired test has twice as few observations by group than a paired test, which leads to a higher standard error and lower calculated power. Therefore, your assumption that the test is paired is optimistic, which is appropriate in the context of your article. The only problem seems to come from your formula which looks like the formula of an unpaired t test.

**Proposition 1**: a quite optimistic assumption would be to assume that all tests are paired and that $R=0.50$ so that $Std.error=\frac{1}{\sqrt{df+1}}$. However, you may find it too optimistic given that many t tests may actually be unpaired, leading to an underestimation by a factor ≥ 2 of the actual standard error and to a major overestimation of the actual power (e.g. 82% instead of ≤ 30%) as shown by the R command pnorm((qnorm(0.975)+qnorm(0.30))*2-qnorm(0.975)).

**Proposition 2 (alternative proposition)**: the theoretically “best” way would be to assess the proportion of t test that are actually unpaired by random sampling of articles containing such tests and reading of the text surrounding the test by a human. Then, the power would be calculated as a weighted mean of powers for paired or unpaired t tests. Given that 23.8% of all tests are t-tests, this improvement may have a non-negligible influence on results, although it should not be major. However, it is probably not worth the complication since the current article is already quite complex.

## Description of the calculation of standard errors

I think that Table A would be clearer if the formula of the Std. error of Cohen’s *d* was given rather than the formula of the effect size *d*. Moreover, on lines 127-137, you should focus on the calculation of the standard error rather than on the calculation of the effect size, especially on lines 130-131. Indeed, the “effect sizes” are “known” (*d*=0.2, 0.5 and 0.8), and lines 127-137 are about calculating the observed effect size in order to calculate its standard error. The difference between observed and theoretical effect size may slightly confuse readers. Explaining on lines 127-137 that it is about the standard error of the effect size should be better.

With that approach, the standard error of the effect size of a $\chi^{2}$ with 1 df can be computed as:

$$Std.err\left( \chi^{2} with 1 df \right)=\frac{2}{\sqrt{N}}$$

Basically, the same formula as that of an unpaired Student’s t test. This is consistent with the fact that the chi-square test is almost equivalent to a Student’s t test that would be performed on the raw binary variables (coded 0 and 1) as if they were quantitative.

I expect that the actual test statistic ($F$, $\chi^{2}$, $r$, $t$) disappear in all formulas of standard errors of Cohen’s d : only the sample size and degrees of freedom should influence the standard error.

The idea of effect size is more complex to understand for three or more groups ($\chi^{2}$ with $df\geq2$ and $F$ statistic with $df_{1}\geq2$). Effect sizes could be generalized to three or more groups as the average of effect sizes of all pairwise comparisons. Another possible generalization is based on equality of R² (variance explained), which would produce slightly different results. With the R², I guess that the standard error of the effect size would be basically $\frac{2}{\sqrt{N}}$ for all tests, where $N$ is the total sample size. The generalization scheme of Cohen’s $d$ used in this article is unclear.

**Propositions**:

1. Use a clear definition of effect size ($d$ statistic) for the Pearson’s $r$ statistic and for multigroup comparisons (such as one based on R²).
2. Give formulas for standard errors of the effect size in Table A
3. Focus on the calculation of the standard error rather than effect size on lines 127-137 of the manuscript, since it is the real problem

Moreover, since mathematical errors are easy to miss, I suggest that you perform, for each statistic ($F$, $\chi^{2}$) and for 2-group and 3-groups scenario, a simulation, with randomly generated groups, checking that the formula gives consistent estimates of the standard errors, and that the power is also consistent with the calculations.

## Assumption D

Appendix S1, page 5, assumption D is: “*The actual statistical power (if p-hacking did not exist) is constant among all tests of the literature where H1 is true*”

For a reader who is not familiar with the methods, this assumption seems to be quite extreme at first. The assumption of a constant effect size (Assumption A) is much more sensible. Moreover, assumptions A and D are contradictory since sample sizes are not constant. Even though contradictory assumptions may sometimes have no bad consequences, depending on the analyses performed, it is always worrisome.

When computing the p-hacking rate from the caliper ratio, you made some assumptions about the statistical power had to be made in order to model the distribution of P-values conditional to their significance. With a very poor statistical power, the distribution of P-values would be uniform on the [0.00-0.05] interval, leading to high caliper ratios for moderate p-hacking rate, while higher statistical powers would lead to a peak probability of P-values close to zero and a moderate caliper ratios for high p-hacking rates.

However, I think that this distributional assumption can be loosened, using the empirical distribution of P-values conditional to the significance (p<0.05). Indeed, publication bias and the p-hacking you described, would not change this conditional distribution. Therefore, you can easily compute the probability of a P-value in the interval [0.039, 0.05] conditional to the decision of p-hacking. This should be very easy to compute with R… Something like mean((pvalue≥0.039)[pvalue<0.05]) where pvalue is the vector of the 691,960 P-values you computed from published articles.

This empirical conditional distribution implicitly takes in account the variance in sample size, effect size, probability of hypothesis truth (a false hypothesis can be interpreted as an effect size equal to zero), and correlations between sample size and effect size. The only important assumption is that the p-hacking rate is completely independent on the sample size, effect size and hypothesis truth for any p>0.05.

This proposition solves half of the problem. Indeed, it defines the distribution of P-value once the p-hacking process has been initiated, but there are two steps before initiating the p-hacking:

1) The first experiment yields a P-value>0.05
2) The researcher decides to p-hack, with a probability *phr*

The probability of step 1 is important to compute.

There are two strategies to compute this step 1:

1) Using an overall statistical power, derived from the empirical distribution of sample sizes, effect size $d$ and probability of hypothesis truth $\theta$.
2) Using the empirical distribution of P-value (corrected for p-hacking)

The second option is probably better because there are very few assumptions. This makes the problem mathematically more complex because the p-hacking rate and correction to the empirical distribution of P-value have to be simultaneously computed.

Overall, I think that you can compute the *phr* based on four empirical probabilities:

1) Empirical probability of significant P-value < 0.039
2) Empirical probability of just-significant P-value (overcaliper) : P-value ∈ [0.039, 0.05)
3) Empirical probability of just-non-significant P-value (undercaliper) : P-value ∈ [0.05, 0.0626)
4) Empirical probability of non-significant P-value > 0.0626 : P-value ∈ [0.0626, 1.00]

The sum of the four probabilities is equal to 1. This is a simplified “histogram” of the empirical distribution of P-values.

I think that these four probabilities can be expressed as a function of these four parameters:

a) P(p-value < 0.039 | p-value <0.05), which can be directly computed from the empirical distribution, since it is not modified by p-hacking
b) P(p-value < 0.05) if there were no p-hacking at all. This cannot be directly computed, since p-hacking inflates this probability. It is an unknown variable that has to be estimated.
c) P(p-value < 0.0626 | p-value>0.05), which can be directly computed from the empirical distribution, since it is not modified by p-hacking thanks to assumption B.
d) The p-hacking rate *phr*, which cannot be directly computed.

The R statistical software makes it easy to solve such equations with the optim() function. Just write a R function taking the two unknown parameters (b) to (d) as input and returning the four parameters (1) to (4) as output. With the optim() function, you may find the solution of (b) and (d) that minimizes the least squares of difference between the observed parameters (1) to (4) and the computed parameters. The solution of least squares must be a **perfect fit**, i.e. the sum of squares MUST be zero. The optim() function is just a lazy solution to avoid mathematically solving the 4-equations 4-variables problem. I did not write down the equations, but the mathematical solution might not be so complex to find.

I did not give deep thought in these solutions, but I hope that it could help you to avoid to make too strong and too contradictory assumptions.

I expect that it does not change so much results and conclusions of your article, but it is worth trying.

Some similar strategy, based on empirical distributions, could be used to estimate the publication bias rate (*pbr*).

This strategy may reduce the impact of the problems of the uncertainty about $\theta$ and $d$, and the assumption of constant effect size, that may be the primary explanation of the central limitation of the article.

From the empirical distribution of P-values, it is even theoretically possible to assess the distribution of effect sizes (including the proportion of effect sizes at zero). It should be possible to create a maximum likelihood estimator of a parametric distribution of effect sizes (e.g. describing the distribution of effect sizes with four or five parameters… maybe assuming a zero-inflated and skewed distribution). It should be possible to estimate simultaneously the p-hacking rate and the distribution of effect sizes but I do not think that it is really relevant for your article. It is just an idea that helps to understand what the empirical P-value distribution represents.

# Minor comments

In the abstract, the first of these two sentences is incomplete and should either be merged with the second sentence or rewritten to clarify it:

« As a result, the false discovery rate, the share of statistical artifacts on all significant results, was under an *a priori* probability of 50% of all 20 hypotheses being true and p-hacking considerable. In this scenario, 17 percent of all statistically significant findings were statistical artifacts rather than substantial results. »

Overall, the English is a bit clumsy. When the manuscript is close enough to its final form, editing from a native English speaker would improve the manuscript.

NB: I am not a native English speaker either.

The first sentence of the Introduction is awkward, because it says that “misconduct [...] endanger scientific integrity” which is a tautology. The sentence makes more sense when replacing the word “integrity” by “validity”.

On lines 42-45:

« Questionable research

practices, such as erroneous rounding results (5-7) and selective reporting of results that confirm the expectations (publication selection bias 7, 8), may even pose a larger problem for the validity of the findings than fraudulently made-up datasets (9). »

You should either add “of” between “rounding” and “results” or remove the “results” word.

In the introduction, the term “test” would be better than “estimator” or “estimate” on lines 66-70. For instance “falsely detected estimates" can be replaced by "falsely positive tests".

Overall, the “Problem” section sums up quite well the statistical theory and practical problem that arises. The “Problem” section should be a sub-section of the Introduction rather than a separate section in my opinion.

On lines 108-109: “*Also, minor reporting inconsistencies in the articles were allowed (e.g., X²*

*instead of χ²).*"

Please, specify that this is allowed by your automatic extraction algorithm rather than by the APA publication manual.

On lines 118-121:

« To compare two-sided (*t*, *r, z*) and one-sided test statistics (*F*, *χ²*), two-sided *p*-values were only computed for the two-sided tests. However, it was impossible to distinguish if a test that allowed for two-sided testing was used only one-sided. »

This is awkward, as it mixes two ideas: First, tests based on *F* and *χ²* statistics are always “one-sided”, i.e. integrate only one tail of the statistic distribution, although they may actually be interpreted as two-sided tests (e.g. comparison of two proportions with a *χ²*). Second, tests based on *t*, *r* and *z* exists in one-sided and two-sided form, leading to different P-values for the same test statistic.

I think that the only important idea to convey is that you assumed that P-values were two-sided for t, r and z-based statistics. You may reformulate the sentences to clear that up.

A two or three sentences Conclusion separate from the Discussion would be nice.

Please rephrase lines 148-152. The statistical power should either be described as the probability of identifying a true effect or as the cumulative distribution of a normal distribution, but not a mix of both in one sentence. Moreover, I recommend to use Cohen’s $d$ and its standard error here, rather than the true mean effect (µ).

Lines 162-163: the *oc* and *uc* are defined by parentheses. This is clear to me, because I know what they stand for, but it would be unclear to a reader not familiar with these ideas.

Lines 173-175: this sentence should be rephrased to clarify that the CT cannot distinguish between the two mechanisms and so, is not enough to assess the false discovery rate.

Line 176: please, define the CR (with its formula) before first use in the manuscript. For instance, you may define the overcaliper (oc), undercaliper (uc) and caliper ratio (CR) near line 165. Or you may just define the CR at line 176 between parentheses: e.g. CR (probability of just-significant result conditional to the result being close to significant, i.e. just significant or just non-significant).

Line 178: please, put pbr between parentheses to clarify that it is an abbreviation of *publication bias rate*.

Line 183: replacing the word “given” by “assuming” would improve the sentence.

Line 196: After “Second, p-hacking is always successful in finding a significant specification”, please mention that you assumed that the distribution of p-value after p-hacking is equivalent to that which would be obtained by drawing a new sample, and so, may have a high probability of being much significant. The words “as this might be the case for researchers that that employ several rounds of drawing" may be replaced by “as if several rounds of drawing were performed until a significant result was obtained”.

At line 200, or in the discussion, you may specify that the fine tuning could lead to a high probability of just-significant results and so, for a moderate probability of p-hacking, it could give high CR values. Such high CR values would be interpreted as major p-hacking with the sample re-drawing mechanism hypothesis.

At line 204: a result is given in the Methods section which is discouraged in articles. However, it serves the purpose of explaining a concept. It may be rephrased to remove the reference to the actual result.

At line 210: “𝐹𝑃𝑝𝑏 which is defined as the product of no underlying true effect (𝜃)". Replace the 𝜃 in parentheses by 1-𝜃.

At line 235-236, you wrote that “*Given the fact that p-hacking is a more general framework that reduces authors' costs of producing significant results, p-hacking might be more prevalent*". This assertion is too subjective and not necessarily true, in my opinion. In my personal experience (Department of Biostatistics), both p-hacking and publication bias seem to be quite common: I have no idea which one is more prevalent overall.

At lines 236-240, some assumptions are described. Maybe one additional assumption should be mentioned: p-hacking and publication bias affect each test (primary, secondary, post hoc) independently. For p-hacking, this makes sense, even if it may be worse on primary analyses than secondary analyses. For publication/reporting bias, there is a selective reporting bias that may impact each test separately, but the classical publication bias (articles kept in the file-drawer) should impact a whole article at once, probably based on the primary analysis.

At line 244: “*In total 43 observations for each year are available.*". This sentence suggests that there 43 observations/year × 43 years = 1849 observations. It should be rephrased… Maybe “In total, 43 observations were available for 43 years (1975 to 2017)” or any equivalent wording (a native English speaker may improve this sentence).

The first sentence of the Results section describes three parts of this section. Explicit subheadings in the results section may help to read the manuscript.

In addition, explicit subheadings of the Methods section may also improve the manuscript, since this section is quite long: e.g. extraction of test statistics, computation of power, estimation of publication bias and p-hacking rate.

Lines 255-257: “In the second step, the test values were transformed into a standard effect sizes metric (Cohen's *d*) to estimate the statistical power. ». You should rather specify that test values were transformed in standard errors of effect sizes. Indeed, Cohen’s *d* are fixed (d=0.2, 0.5, 0.8), and only its standard error must be computed from the article data.

Fig 2: please, use consistently a lower case or upper case ‘k’ character for the number of articles, but not both. Moreover, I think that Fig 2 should be renamed as Fig 1 since there is currently no Fig 1.

Table 1: one digit after the decimal point should be enough.

Lines 271-273: “*On the article level, the number of included test values, approximated by the mean sample size for each test, was extremely right-skewed (mean: 791.144, median: 81), mainly caused by studies with a very large number of observations.*”

the sentence does not make much sense to me. The mean sample size is not an approximation of the number of test values per article.

The next sentence “*Compared with the sample size found in psychology (53), the sample size is nearly twice as large in both mean and median.*" should contain the mean and median sample sizes specified in reference 53.

On lines 282-284: “*However, in a robustness check, there were no differences in the mean p-value, the calipers used for publication selection bias, and the type of test values for the neighboring years 2015 and 2016*.". Do you mean “caliper ratio” rather than calipers? Do you mean “2015 vs 2016”?

« *However, a significant but very small difference (2.7 percentage points) was found in the statistical power detecting a medium effect (d = 0.5).* ». In which direction? Please, specify the statistical power for each of the compared periods (2015 and 2016 as far as I understand).

Line 286 : “*On the level of test values, the F-Test was the most-used test statistic, with 67.2%.*”. Please, add “of all tests” at the end of the sentence.

Line 289: “*t-tests were used in 23,8%*". Please, use a decimal point as digit separator rather than a comma.

Fig 3: nice figure! For panel B, could you add a digit to the percentages to avoid showing to “equal” percentages (4% and 4%) for two different bars.

Line 308: *(CR = 3/2 = 60%).* The ratio 3/2 is equal to 1.5, which is inconsistent with the value 60% given. I guess that CR = 3/5=60% and that 3/2 is the odds of the overcaliper vs the undercaliper.

Line 310-311: “*providing evidence for publication selection bias in the psychological literature*”… publication selection bias OR p-hacking.

Lines 313-315: “*For deciles with a smaller number of observations statistically significant at the 5% level are much more common than for lower thresholds (e.g. 1% and 0.1%), especially when compared with deciles with a larger number of observations*” please reword to clarify. I propose “*For small sample sizes, the proportion of P-values below 0.001 was much lower than for large sample sizes while the proportion of P-values below 0.05 was only slightly lower (Table B, S1 Appendix)*”.

This is an interesting result! However, it suggests either a publication bias or p-hacking or both. Consequently, the previous sentence “*The presence of publication selection bias could also be seen when looking at different significant thresholds for deciles of the underlying number of observations.*” should mention “OR p-hacking” or “assuming no p-hacking”.

Lines 320-321. I guess that you assumed that 50% of effects were d=0 and 50% were d=0.50, but the wording is not very clear about that. Please reword to clarify that.

Line 337: *“Both measures of publication selection bias, publication bias, and p-hacking"*

Please, put publication bias and p-hacking between parentheses to clarify that they are the two forms of publication selection bias.

Fig 5: replace the legend “meassures” by “effect size”.

Line 356: “*half of the existing effects are detectable*”. Actually, half of the existing **medium** effects are detectable.

Lines 365-366. You mention the “*retrospective statistical power*”. Is not that a remnant of the older manuscript, when the average effect size was meta-analyzed? I propose to replace that by “*for a medium effect size (d=0.50)*”.

Line 366… The reference to “Fig 5” is incorrect. It is “Fig 6”.

Lines 366-367: you mention that up to 17.8% of significant effects would be false, but the actual figure suggests that the FDR is greater than 20% for most years. Is there an error?

Lines 371-372: “*This lower bound is the case for both scenarios, publication bias and no publication selection bias.*” Please reword in one or two sentences to clarify. I understand that this sentence contains two ideas:

1. Assuming there is no p-hacking, the FDR is independent of the level of publication bias
2. The scenario of pure publication bias is the most optimistic for the FDR

Line 375: the term “FDR_inf_” is mentioned without explanation. When looking at the figure, I understood that FDR_inf_ means “inflated FDR” and is synonymous of “FDR assuming pure p-hacking without publication bias”. I propose that you avoid using FDR_inf_ unless you first define it in the main manuscript. Moreover, the word “*decreased*” should be replaced by “decrease in” and “*lower p-hacking rate*” should be replaced by “lowering of the p-hacking rate” because it describes a temporal trend in the subfield.

Lines 378-378: “*So far, the FDR was only reported based on the estimated retrospective statistical power and the 5%-caliper p-hacking bias estimator.*" Again, the “retrospective statistical power” is described but it is not clear how it is computed. Is it based on the effect size (d=0.2, 0.5 or 0.80) and standard error of Cohen’s *d* calculated from articles?

Lines 389-390: “*the a priori probability[…]*”. I propose to rephrase that to “*the proportion of true hypotheses (θ)*” in order to be consistent with other paragraphs, using a frequentist vocabulary rather than a Bayesian one.

Line 395: “*[…] but probably yielded too low FDR*". I propose to reword "*probably underestimated the actual FDR*”.

Line 407: “*Relying on a more restricted sample* […]" I guess that you mean that articles with few statistical tests are most likely to contain tests with statistical tests that are relevant to the research question. You may rephrase this sentence to clarify that.

Line 423-424: “*For χ²-tests with df = 1, a larger statistical power could be observed around 20 percentage points*." Do you mean that the power for *χ²-tests with df = 1* was 20% higher than the power for tests related to Pearson’s r? You should provide the statistical power rather than the difference of statistical powers; it would be clearer.

Lines 430-432: the syntax of the sentence seems to be incorrect. Replacing “*with 31.9% or 33.8%, much lower.*" by "would be 31.9% or 33.8%” seems to fix the problem.

The discussion about the Central Limitation is nice, however I think that a few additional things may be discussed, in relation with the difference between primary and secondary analyses. Effect sizes (Cohen’s d) were assumed to be homogeneous, and so, for a given article, the power is expected be quite homogeneous between analyses (this is already mentioned in the limitations paragraph). I would expect that the actual effect size and statistical power would be quite poor for the primary analysis but that effect sizes and statistical power could be excellent for many secondary analyses that tests “obvious” hypotheses such as correlation between measures that are very closely related. For those analyses, $\theta$ (proportion of true hypotheses) and $d$ (effect size) may both be very high, leading to a high share of significant results (p<0.05), and even a good rate of quite significant results (p<0.01). Moreover, the p-hacking and publication bias most likely impact the primary analysis rather than secondary analyses.

With those mechanisms, I can understand that the share of significant results is high, because of secondary analyses showing obvious correlations, while the p-hacking and publication bias are underestimated because they are related to approximatively 1/17^th^ of test statistics since there are, on average 17 tests per article.

Table 2: replace the word “*Exemplary*” by “Example of”.

Lines 464-465. “*Despite the positive development in recent years, the statistical power is still low*" Due to the central limitation in this article, it is hard to tell whether statistical power is low. I think that you can tell that sample sizes are small and statistical power is low for medium (d=0.50) and large effect sizes (d=0.80)… But statistical power may actually be good for huge effect sizes that may be actually met for irrelevant secondary analyses testing obvious correlations.

Lines 465-466. “*In the data, patterns of publication selection bias were detected but hardly changed over time.*” you may add “*or p-hacking*”

Line 477. replace “*resukts*” by “results”.

Lines 509-525. As this is the end of the discussion, a great freedom of discussion about future perspectives is given to authors; meaning that some of the content of this paragraph can be an opinion rather than scientific facts.

That is why I do not ask authors to modify this part.

In my opinion, mandatory power analyses before the study are not a great idea. In medicine, in France, pre-study power analyses are mandatory to get funding, but lead to many cheated hypotheses, based on ridiculous effect sizes. Indeed, everybody has to reach the “≥ 80%” power to get funding, while the total capacity of inclusion on a few years can rarely be enough to get enough power. Therefore, everybody cheats by providing fake power analyses based on ridiculous hypotheses with unrealistic effect sizes. This is so common practice that I even saw once a protocol update to reduce the sample size because inclusions were slower than initially predicted. Instead of computing the reduced power (e.g. 80% → 70%), the statistics paragraph was updated to assume that the reality changed to accommodate the slower inclusion rate (power: 80% → 80%, effect size increased).

*Post hoc* power analyses performed by readers of the article are more relevant, although they would not encourage good research practice.

In my opinion, preregistration should be encouraged but should not become mandatory, otherwise there is a risk that it encourages scientific misconduct to make things more consistent between the published article and the pre-registered protocol when major deviations to the protocol happened (for good or bad reasons).

Appendix S1, page 1, section 1.1. "*As laid out in the main article, is done by assuming an a priori plausible effect size.*". Please add ‘it’ before ‘is’ or reword to make the sentence syntax valid.

Appendix S1, page 1, assumption A: please add “all analyses (primary and secondary)” in the description of the assumption to clarify the fact that it is not only about primary analyses.

Appendix S1, equation A… I guess that it is $\left| z \right|$ rather than $z$, because all tests are assumed to be two-sided and the significance threshold $th=1.96$. Please, add the absolute value in the equation, if appropriate.

Appendix S1, page 3: “*Although the large number of z-values in the study at hand provides the CT with sufficient statistical power, another reason to choose wider intervals is the accuracy of the test values reported in the primary studies that are usually presented with two decimal places and add further noise to the distribution.*"

I first read that sentence too fast and improperly read “P-value” instead of “test values”… To avoid such reading error, I suggest that you clarify that by adding “(e.g. $\chi^{2}=3.86$)” after “*test values*”.

Appendix S1, Assumption B: please add the words “for any P-value in the interval” before “(α, 1]”. Also specify that you assume that p-hacking is also independent of the sample size, effect size or actual truth of the hypotheses tested.

Appendix S1, Fig A. This is a nice figure, but there is a reading difficulty: at step this, there is a box “[P_1_ (phr)]” related to the probability of p-hacking. The choice of p-hacking is only drawn once, then random samples are iteratively drawn until significance is met. This can be understood because the probability is described as P_1_(phr) rather than P_i_(phr). This is very subtle. Another way to express the same idea would be to add a few boxes at the top of the figure for the initial step:

1) Planned analysis P-value ≥ α (0.05) (top box of the figure)
2) Decision of p-hacking P(phr) or not p-hacking 1-P(phr)

In the Fig A legend, you may also specify that this process is equivalent to repeating the experiment on new independent samples until a significant test is obtained.

Appendix S1, page 4: “*The first step in the process of p-hacking, as shown in Fig A 𝜃 defines if there is an underlying effect (H1 true) or no underlying effect (H0 true)*"

This should be reworded… The existence of an underlying effect is not controlled by a human, while p-hacking is a human process.

Appendix S1, page 6: “*Equation B shows the* ***conditional*** *probability of a just significant*". Is not that the “unconditional probability”?

Appendix S1, Fig B: very nice work and figure. Please specify that N is the number of tests and K the number of articles, in the figure legend or within the figure itself. Moreover, I propose that you add a few keywords to the title of the figure. Rather than “*Truly and falsely detected test values in the data extraction process*” I propose “Truly and falsely detected test values in the automated data extraction process versus manual (human) data extraction”

Table C. please define “small”, “medium” and “large” effect sizes in the table legend. I understand that for d, they are d=0.2, d=0.5 and d=0.8, but it is unclear when the effect size is expressed as a correlation coefficient r.

The “CT” in the headings of Table C seem to be actually the caliper ratio CR.

For a prettier table, I suggest that you specify the last digit of numbers, even if it is zero. For instance, write 0.150 for the statistical power rather than 0.15.

Last sentence of page 13: “*All in all, there is robust evidence for a mostly time-invariant substantial publication selection bias rate in the psychological literature without any signs of decline.*"

I agree with that assertion, because the different limitations of your analyses probably have the same impact in all periods, so that time trends are interpretable. Therefore, I think that this finding may be part of the conclusion of your article.

For the time-trend analysis described in Table E, is the autocorrelation taken in account? If not, an autoregressive model, such as AR(1) may be used. However, I expect a weak autocorrelation.

Legend of Fig C and Fig D: the subfield “rest” should be renamed to “other”.

Table E and Table G show yearly slopes. The values of slopes are very small and too heavily rounded. I propose that you express the slope for 10 years rather than 1 year and that you express them as percentages. For instance, +0.002 / year becomes +2% / 10-years. You may ensure that there is a two-digits precision.

Fig D. Add “for a medium effect size (*d*=0.50)” in the figure legend.

Fig E. the term “inflated FDR” is very little used in the article. You should either completely avoid it, or rename it so its meaning is obvious… I propose to name the two FDRs: “FDR for pure publication bias” (panel A of the figure) and “FDR for pure p-hacking” (panel B of the figure). Eventually, an asterisk may be added to mention the fact that FDR for pure publication bias is equal to the FDR assuming no publication selection bias at all, so that it only depends on statistical power and proportion of true hypotheses (θ).

Appendix S1, page 19, section 2.4: “*In addition to the scenarios presented in Table 2 in the main text, the scenarios are also presented graphically in Fig E.*”. Reference error: it is Fig F and not Fig E.
