# Supplementary material for: Are most published research findings false? Trends in statistical power, publication selection bias, and the false discovery rate in psychology (1975–2017)
Source: PLoS One. 2023 Oct 17;18(10):e0292717. doi: 10.1371/journal.pone.0292717 (PMC10581498; doi:10.1371/journal.pone.0292717)
Supplement: Attachment — Submitted filename: response_rev_rev_rev_rev_rev.docx [file pone.0292717.s013.docx]

# Summary

The manuscript has been improved and is now quite good in my opinion. The objective, methods, results and discussion are consistent. Limitations are well acknowledged.

I do not have major comments but have many minor comments and suggestions, spotting stylistic issues and minor errors.

Maybe the worst issue lies in probable errors of equations shown in the Appendix. I hope that these equations were not used for computations.

I would like to thank the author for the great patience he had, to resubmit repeatedly his manuscript, responding to all my comments for several rounds.

Help from a native English speaker would be welcome.

# Detailed comments

In the abstract (lines 18-20) : « *As a result, the false discovery rate, the share of*

*statistical artifacts on all significant results, assuming a proportion of true hypotheses (θ) of 50% of all hypotheses being true, and p-hacking was with 17.7 percent considerable.* »

This sentence is unclear and should be reworded… I propose to change it to “*The share of false discoveries among all significant results was estimated at 17.7%, assuming a proportion θ=50% of all hypotheses being true and assuming that p-hacking is the only mechanism generating an overcaliper, i.e. a higher proportion of just-significant results compared to just-insignificant results”*

Changed to:

The share of false discoveries among all significant results was estimated at 17.7%, assuming a proportion θ=50% of all hypotheses being true and assuming that p-hacking is the only mechanism generating a higher proportion of just significant results compared to just nonsignificant results.

On lines 42-44, the referencer mentioned in the sentence do not directly support the sentence: “*Questionable research practices, such as erroneous rounding of results (4-6) and selective reporting of results that confirm the expectations (publication selection bias 6, 7), may even pose a larger problem for the validity of the findings than fraudulently made-up datasets (8).*”

Indeed, the reference is neither about the validity of findings of fraudulent articles (which is scientifically zero, anyway) nor about the deleterious impact of fraudulent articles on the scientific knowledge overall, but only about the frequency of minor misconduct. The impact of fraud and minor misconduct on scientific knowledge is hard to know.

Changed to:

Questionable research practices, such as erroneous rounding of results (1-3) and selective reporting of results that confirm the expectations (publication selection bias 3, 4), may even pose a larger problem for the validity because of the very low prevalence of than fraudulently made-up datasets (5).

The legend of “Fig 1” is somewhat misleading “*The Truth table visualizes the different possible errors made by a statistical test: detecting a nonexisting effect (FPR) or missing an existing one (1-pow). The FDR, as a joint measure of both errors, allows quantifying the FP share on all positive statistical effects (TP + FP)."*

Indeed, knowing the FPR and 1-pow is not enough to compute the FDR (it depends on θ). You may just change the sentence to “*The FDR allows quantifying the FP share on all positive statistical effects (TP + FP)”*, or mention that this depends on FPR, power and θ.

Changed to:

The FDR makes it possible to quantify the FP share out of all positive statistical effects (TP + FP).

On lines 44-46: “The article at hand shows the development of statistical power and publication selection bias in the form of publication bias or p-hacking in psychological articles published between 1975 and 2017." I am not sure that the term “development” is appropriate here. Maybe “state and evolution” would be better. Same comment for lines 459-460 (start of the Discussion).

Changed

On lines 79-82, the sentence “*Publication bias is defined as searching for significant results in new datasets while nonsignificant results remain in the file drawer and are therefore, not published*” suggest that experiments are repeated until they lead to significant result. This is not the usual definition of publication bias, which is based on keeping nonsignificant results in the file drawer, without doing anything else. Repeating the experiment is possible (especially in *in vitro* and small animal studies) but leads to a very different bias. At first glance, the FDR seems to be unchanged… but actually, the proportion of true hypotheses tested (θ) is lowered by the fact of repeating experiment with non-significant results, which have a higher probability of testing a wrong hypothesis. If all the repeated experiments testing the same hypothesis by the same searchers are considered as one big experiment based on the “repeat until it works” methodology, then, θ is unchanged but the FPR is inflated as in p-hacking.

This problem can be solved by rewording the sentence, such as “*Publication bias is defined as publishing only studies with significant results while studies with non-significant results remain in the file drawer and are therefore, not published”*.

Changed to:

Publication bias is defined as publishing only studies with significant results, while studies with nonsignificant results remain in the file drawer and are therefore not published (26).

On lines 81-83 : “*A bias closely related to publication bias is selective reporting bias,*

*where significant results within one dataset are reported, whereas nonsignificant results remain unpublished.*”

Inserting the words “*within the same dataset*” after “*nonsignificant results*” would make the sentence clearer.

Changed to:

A bias closely related to publication bias is selective reporting bias, where significant results within a given dataset are reported, whereas nonsignificant results within the same dataset remain unpublished.

On lines 90-93: “*P-hacking, in contrast, increases the number of FP by transforming TN to FP (for theoretical considerations, see 1, 12), as the resulting FP results form a flood of statistical artifacts (28), outnumbering the TP effects.*"

The sentence would be correct if “*the number of FP*” is changed to “*the FDR*” and removing “*results*” after “*FP*” and before “*form a flood*”.

Changed to:

P-hacking, in contrast, increases the FDR by transforming TN to FP (for theoretical considerations, see 1, 12), as the resulting FP form a flood of statistical artifacts (28), outnumbering the TP effects.

On lines 98-99 : “*The automatic extraction routine covered full-texts in Html and pdf form, using OCR to recover the texts from the pdf files.*”

Please, use upper case letters for abbreviations HTML and PDF, and specify the non-abbreviated form of OCR at first occurrence (Optical Character Recognition).

Changed accordingly.

Lines 124-125 : “*To this end, we used a priori set plausible true effect sizes for small (d = 0.2), medium (d = 0.5) and large 125 (d = 0.8) effect sizes (12, 35).*”

The words “used” and “set” are redundant; remove one of them. Proposals: “*we set a priori*” or “*we used a priori*”.

Changed to:

To this end, we used a priori plausible true effect sizes for small (d = 0.2), medium (d = 0.5) and large (d = 0.8) effect sizes (12, 35).

On lines 130-132 “*For F, χ², and r the standard error was defined as: 𝜎𝑖 = 2/√𝑁, whereas for t an additional assumption on paired tests and the correlation between observations (r) has to be made*”, please specify that you made the optimistic assumption that the two groups were equal in size (N_1_=N_2_=N/2).

Changed to:

For F, χ², and r the standard error was defined as: $\sigma_{i}=2/\sqrt{N}$, whereas for t an additional assumption on paired tests with equal group sizes and the correlation between observations (r) has to be made.

The Supporting Information specify “*Therefore, multi-group comparisons were dropped from the analysis*” on page 2. The main body of the manuscript is unclear on that. I suggest to mention that you dropped statistics with ≥ 2 degrees of freedom, within, before or after the sentence of lines 128-130: “*The conversion of the test values into standard errors in Cohen's d metric was possible for F, χ², both with two groups (df = 1) and r, t but not for z because no information on the sample size was available.*”

You may even refer to the Supporting Information in the manuscript.

I clarified:

Multi-group comparisons for F and χ² (df1 > 1) were therefore not included.

On line 135 “*error of the Pearson correlation coefficient and the, in respect of the standard error equivalent*”, the words “*and the*” should be dropped unless I did not understand the sentence; in that case, it should be reworded.

Changed to:

Besides Cohen's d metric, the standard error of the Pearson correlation coefficient and the biserial correlation coefficients (36) were also calculated and transformed in Fisher's z for the power estimation (see the section on robustness).

On equation shown on line 140: parentheses around d/σ_i_ are extraneous.

Changed.

On lines 158-159: “*However also the 10%- [0.0311, 0.05), [0.05, 0.078) and 1%-caliper [0.048, 0.05), [0.05, 0.052) were estimated.*”

Moving the “*also*” word just before “*estimated*” would make the sentence prettier. I am not a native English speaker, but I am used to see the “*also*” word close to the verb.

Changed to:

However the 10% [0.0311, 0.05), [0.05, 0.078) and 1% caliper [0.048, 0.05), [0.05, 0.052) were also estimated.

On lines 167-169: “*Although the CT can be used to estimate the rate of pure publication bias or p-hacking, it is impossible to identify if publication bias, p-hacking, or a combination of both strategies leads to an overrepresentation of just significant results.*”

The second part of the sentence seems to say that publication bias and p-hacking combined together may not lead to an overcaliper, while actually the problem is that, given an overcaliper, it is not possible to know whether it is explained by p-hacking, publication bias or both. The sentence should be reworded to make that clear.

Changed to:

Although the CT can be used to estimate the rate of pure publication bias or p-hacking, it is not possible to identify if publication bias, p-hacking, or a combination of both strategies leads to an overrepresentation of just significant results (overcaliper).

Lines 187-191: the two sentences could be merged to make them shorter; the second one is very clear.

Changed to:

Some assumptions have to be made for both estimators of publication bias and p-hacking. First, publication selection bias is implemented irrespective of the nonsignificant p-value achieved initially. This means that a strongly nonsignificant p-value is equally likely to be treated by publication selection bias and dropped with an equal probability in the case of publication bias as it is in the case of p-hacking (e.g., borderline significant results, p = 0.051 or far off p = 0.9).

Lines 202-203: “*The probability of finding statistically significant results that are true is not affected bypublication bias.*” is redundant with the previous sentence.

The sentence “The probability of finding statistically significant results that are true is not affected by publication bias.” Is pruned

Line 205: “*which is defined as the product of no underlying true effect (𝜃) and the set*”. Please, put (1-θ) in parentheses rather than (θ).

Changed

Lines 212-213: “*P-hacking works slightly differently as nonsignificant results (𝐹𝑁𝑝ℎ and 𝑇𝑁𝑝ℎ) are replaced with a significant result (that add to 𝑇𝑃𝑝ℎ and 𝐹𝑃𝑝ℎ rather than being dropped completely).*”. For consistency, “*a significant result*” should be replace by “*significant results*”.

Changed

Line 214 “*while the dropped studies were*”. Please change “*were*” to “*are*”, otherwise one would think that the last part of the sentence refers to publication bias rather than p-hacking.

Changed

Lines 234-235: “*Especially publication bias 𝜃 may affect complete articles by leaving the whole results unpublished.*" Replacing “*especially*” by “*although*” and adding “*actually*” before “*affect*” would make the sentence more consistent with the previous one.

Changed

Line 252: “sample size dropped out" would be clearer as “sample size were dropped out”.

Changed

Lines 253-254: “*After this step, 35,515 articles containing 487,996 test values remain in the analysis sample*." Please, use “*remained*” rather than “*remain*” for time consistency.

Changed

Line 259: the word “*Description*” would be better than “*Descriptives*”.

Changed

Figure 2 (flow chart) seems to be inconsistent.

It specifies that there are 734,748 computable P-values and 487,996 P-values for which the statistical power is computable. Therefore, 734,748 – 487,996 = 246,752 excluded P-values are expected, which is consistent with specific exclusions 18,062 + 24,331 + 28 + 184,176 + 20,155 = 246,752 but inconsistent with the overall number of exclusions 42,773 specified at the top of the exclusion box. This number probably comes from an old version and should be updated.

Thanks for pointing at the error, I corrected and checked Figure 2.

The “!=” symbol should be replaced by “≠” if possible.

Changed

In table 1, please use “1^st^ quantile” and “3^rd^ quantile” in the table header, rather than “1. Quantile” and “3. Quantile”.

Changed

On lines 269-271: “*compared with the sample size found in psychology (41: 337), the sample size is more than twice as large as the mean sample size (all years pooled: 207) and substantially larger than the median sample size (48.4, 32.0, 40.0 for the years 1977, 1995, 2006).*”

I understand that (41: 337) is a reference to an article and that this sentence compares the mean sample size. To make this clearer, I propose to add a few words, such as “*compared with the mean sample size found in psychology in the literature (~~41: 337~~217.96, 211.03 and 195.78 in years 1977, 1995, 2006, see (41)), the mean sample size in our study is more than twice as large ~~as the mean sample size~~ (all years pooled: ~~207~~504) and substantially larger than the median sample size (~~48.4, 32.0, 40.0 for the years 1977, 1995, 2006~~all years pooled: 61.5).*”

Moreover, a comparison of the median sample size to the literature would be more relevant, as it is far less influenced by outliers. The median sample size seems to be close to that of the literature.

Changed to:

Compared with the mean sample size found in psychology, as documented in the literature (217.96, 211.03 and 195.78 in the years 1977, 1995, 2006, see 41: 338), the mean sample size in our study is more than twice as large (all years pooled: 504). Much smaller than the mean sample size in our study is the median sample size (all years pooled 61.5), which is also more comparable to the median sample sizes found in the psychological literature (48.40, 32.00, 40,00 in the years 1977, 1995, 2006, see 41: 338).

On line 280: “*there were no differences in the mean p-value*"

Did you mean “*there were no differences in the mean number of p-values” or* “*there were no differences in the mean p-value*"?

Indeed, the main difference that I would expect with OCR in PDFs is to miss some P-values.

The sentence is about the mean p-value over the PDF and HTML exports.

On line 309-310: “*The presence of publication selection bias could also be seen when looking*

*310 at different significant thresholds for deciles of the underlying number of observations*" should be reworded by replacing “*significant thresholds*” by “*significance threshold*” and “*for deciles*” by “*and stratifying the analysis by deciles*”.

Changed

Table B of “S1 Appendix” is very nice. Is it possible to add the computation of the caliper ratio according to decile or quintile of sample size in order to assess whether publication selection bias is dependent on the sample size.

Changed, I furthermore added:

For the 5%- and 10% CR a increasing imbalance of just significant and nonsignificant results is observed that may, contrary to the expectations, point to the fact that larger studies are more affected by publication selection bias.

In Figure 4A and figure 4B, could you change the y-axis labels to “publication bias rate” and “p-hacking rate”? That would make it clearer that these are the publication selection bias rates rather, than, for instance, the FDR under different hypotheses.

Changed

Lines 370-371: “*This low FDR is the case for both no publication selection bias at all and pure publication bias.*”. Replacing “*is the case*” by “*is the same*” would make things clearer.

Changed

Lines 390: “*The minimum of the FDR, given[…]*”. Adding “*theoretical*” between “*The*” and minimum would clear up things.

Changed

Lines 403-404: “*Relying on this more restricted sample may contain test values that are relevant for the research question and exclude test values (e.g., manipulation checks or model fit statistics).*”. I would expect words associated to “*exclude test values*” such as “*exclude irrelevant test values*”.

Changed

Lines 406-407: “*In contrast, the share of just significant values from the caliper test, the basis for the publication selection bias estimate, was larger.*"

A few numeric values should be given rather than completely relying on the appendix. For instance, you may add “*for articles with fewer than 5 tests (5%-CR: 0.646) than for the main analysis (5%-CR: 0.613)*” after “*from the caliper test*”.

Changed

Line 417 “*have the by far largest*" should be "*have* *by far the largest*”.

Changed

The section about the “Central limitation” is very good in my opinion.

Thanks

In Table 2, some numeric values are “copied” from above by use of the quote-unquote (‘’) symbol. I found it slightly confusing and would recommend to copy the actual numeric values. The same remark can be done for Table A.

Changed

Lines 461-462: “(*both in the form of publication bias and p-hacking)*". This suggest that you showed evidence that both forms exist. I propose “*(in the form of publication bias, p-hacking or both)*”.

Changed

Line 463: “*no substantive*" should be replaced by "*no substantial*”.

Changed

Line 465: “*maybe*” should be replaced by “*may be*” (in two words).

Changed

Lines 478-479: “*A robustness check shows that primary research outcomes were only poorly covered (for details, see section 2.1. in S1 Appendix).*” Following the reference to the section, the type of robustness check is clear, but it is not clear from the manuscript’s text. I propose to add a few words, such as “*A robustness check, comparing manual to automatic data extraction* *shows that*[…]”

Changed

Line 481: “*This unobserved heterogeneity[…]*" should be replaced by “*The unobserved heterogeneity[…]*”.

Changed

Lines 484-485: “the relative prevalence” should be replaced by “*their relative prevalence*”.

Changed

The end of the “*Discussion and Conclusion*” section provides perspectives of interventions that could contribute to reduce the FDR. This is okay, but I would expect an additional paragraph of conclusion, directly related to the results of the study, such as “We showed that studies in psychology tend to be underpowered and have signs of p-hacking or publication bias, leading to potentially high number of false discoveries; however, we failed to estimate precisely the share of false discoveries as too many unknown parameters influence this estimate.”.

I clarified:

To sum up, findings reported in the psychological literature tend to be underpowered and shows signs of p-hacking or publication bias, which may lead to a high number of false discoveries; however, the estimations rely on several assumptions and unknown parameters that may influence the estimates. Nonetheless, three interventions can be laid out that may be of help to decrease the FDR and increase the credibility of scientific findings: first, conducting mandatory power analyses before a study; second, pre-registering the research design, along with a complete model specification; and third, developing clear-cut reporting guidelines on how to present statistical tests.

In Appendix, page 2, Table A. The formula $2/\sqrt{N}$ seems to have been truncated. The full formula should be set. The double-quote ("), used to refer to the formula above, makes the table harder to read. I propose that you repeat the formula or that you use merged table cells with visible borders, so that the fact that the same formula is used would be obvious.

The legend of the table should contain additional information: the fact that N represents the total sample size, including all groups. The fact that σ_d_ is the standard error of Cohen’s d and σ_r_ is the standard error of the Pearson’s R correlation coefficient.

Added:

$\sigma_{d}$ is the standard error of Cohen’s d, $\sigma_{r}$ the standard error of Pearson’s correlation coefficient. N represents the total sample size for all groups.

In Appendix, Fig A. This figure is a bit confusing because it suggests that the choice of p-hacking is repeated after each draw of z_i_, contrary to assumption F. The only clue to the fact that the choice is made only once is the subscript 1 in P_1_. Maybe showing the choice before the first z_i_ draw would make things clearer. Fortunately, assumption F is well stated in a dedicated paragraph.

Thanks for your remark, I agree that the figure alone might be difficult to read. The alternative versions (e.g. putting the “decision” to engage in publication selection bias before collecting the data seems counterintuitive to me. Therefore I decided to leave the Fig A as is.

In Appendix S1, page 6: “*Especially Assumption F may be problematic if researchers publish an undesired nonsignificant result because the search or production costs of a significant result outrun the returns*”

It is not that much a problem, in my opinion, because the real p-hacking mechanism is not due to re-experimenting most of the time but is based on modifying selection criteria, outcomes and adjustment variables. Sometimes, new P-values are “almost” independent of the original P-value while other times, they are not much different.

If you wish to mention a limitation of this set of assumption, I would suggest to mention that the assumption that draw of new P-values are independent of the first draw is probably erroneous.

Now pruned

On page 6, 𝑝𝑂𝐶_𝑢𝑛𝑏𝑖𝑜𝑎𝑠𝑒𝑑 should be changed to *p_oc_unbiased_.*

Changed

Equations B to G are not very consistent in their mathematical notation.

Equation F and G use $p_{oc_{\alpha}}$ and $p_{oc_{pow}}$ and I would expect equations C and E to define $p_{oc_{ar}}$ and $p_{uc_{ar}}$.

There is a typo in Equation C. It defines $p_{uc\_unbiased}$ rather than $p_{oc\_unbiased}$. Similarly, Equation E should be based on $p_{uc\_unbiased}$ rather than $p_{oc\_unbiased}$.

There is perhaps a terminological confusion over-caliper is the value larger than the significance threshold (in respect to z), meaning that the overcaliper includes all just significant values (p slightly under p=0.05).

Equation B seems to be half-correct in my opinion.

Indeed, if $Z\sim\mathcal{N}(z,1)$

$$p_{oc_{unbiased}}=\Pr\left( th<\left| Z \right|\leq th+c \right)$$

$$=\Pr\left( th<Z\leq th+c \right)+\Pr\left( -th-c<Z\leq-th \right)$$

And $\Pr\left( th<Z\leq th+c \right)=\Pr\left( Z\leq th+c \right)-\Pr\left( Z\leq th \right)$

$$=\Pr\left( Z-z\leq th+c-z \right)-\Pr\left( Z-z\leq th-z \right)$$

$$=\phi\left( th+c-z \right)-\phi(th-z)$$

Because $Z-z\sim\mathcal{N}\left( 0,1 \right)$

Similarly, $\Pr\left( -th-c<Z\leq-th \right)=\phi\left( -th-z \right)-\phi\left( -th-z-c \right)$

Therefore, $p_{oc_{unbiased}}=\phi\left( th+c-z \right)-\phi\left( th-z \right)+\phi\left( -th-z \right)-\phi\left( -th-z-c \right)$

Below, is the R code of three equivalent R functions, computing $p_{oc_{unbiased}}$.

```
pocu1=function(th,z,c) { # helped by extra params of pnorm
	(pnorm(th+c,mean=z)-pnorm(th,mean=z))+
	(pnorm(-th,mean=z)-pnorm(-th-c,mean=z))
}
pocu2=function(th,z,c) { # without extra params of pnorm
	(pnorm(th+c-z)-pnorm(th-z))+
	(pnorm(-th-z)-pnorm(-th-c-z))
}
pocu3=function(th,z,c,R=1e7) { # simple simulation
	zz=abs(rnorm(R,mean=z,sd=1))
	mean(zz>th & zz<=th+c)
}

pocu1(1.96,z=1.5,0.5) # very large caliper (to make simulations accurate)
pocu2(1.96,z=1.5,0.5)
pocu3(1.96,z=1.5,0.5)

pocu1(1.96,z=-0.3,0.5) # very large caliper (to make simulations accurate)
pocu2(1.96,z=-0.3,0.5)
pocu3(1.96,z=-0.3,0.5)
```

Equation C seems to have the same error.

The R code for unbiased undercaliper is:

```
pucu2=function(th,z,c) {
	(pnorm(th-z)-pnorm(th-c-z))+
	(pnorm(-th+c-z)-pnorm(-th-z))
}
pucu3=function(th,z,c,R=1e7) {
	zz=abs(rnorm(R,mean=z,sd=1))
	mean(zz>th-c & zz<=th)
}
pucu4=function(th,z,c) {
	pocu2(th-c,z,c)
}
```

Thanks for the pointer, the equation is now changed. In the code the correct formulars were used. However, in the process of re-checking the code I found another slight error, that reduced the estimate of p-hacking slightly (from 9.9 to 8.2%, given a statistical power with a medium effect and theta = 0.5). The implications for the inflated FDR therefore changed also slightly.

I hope that you used the correct formula in your actual code and that equations B and D are copy errors.

On page 10 of the Appendix: “*There was no significant difference between the detected results and the falsely non-detected results reported in-text that were missed by the algorithm t(266)=-0.948; p=0.344.*” It would be clearer if the words “*of the mean P-value*” were inserted between “*no significant difference*” and “*between the detected*”.

Changed

The statistical validity of this test is dubious. Indeed, adjustment on the study is not enough to fix the non-independence of test results within an article. All tests in a table (undetected by your algorithm) may share a large part of the data. For instance, sometimes, the first column of a table contain unadjusted P-values while the second column contain adjusted P-values : these P-values are correlated. Moreover, the text may refer mention P-values that are also shown in the table. Therefore, within each group (detect and undetected P-values) and between groups, P-values may be correlated. Theoretically, with a much larger sample of articles, it could be possible to perform a statistically valid comparison, by using bootstrap or jackknife on whole articles, with the only assumption that P-values of different articles are independent. With only seven articles, you cannot do that.

However, most probably, your t(266) test provides P-value smaller than it should (liberal test); as they are quite large, I think the result can still be interpreted.

I suggest that you mention that you neglected the correlation of P-values within an article and that these t(266) test is probably liberal.

I completely agree and clarified in a footnote:

Possible dependencies of p-values within an article were not considered because of the low number of included articles. The modelling strategy ignoring these possible dependencies might therefore be more liberal, detecting inexsisting differences.

On page 10 of the Appendix: “*The limitation of the subset of 5 articles was*”. I guess you meant “*The limitation of the subset* *of articles with ≤ 5 tests was*”.

Changed

On page 10 of the Appendix: “*In total, only 25% of all primary research outcomes could be extracted*.” I guess you meant “*extracted automatically*”.

Changed

On page 13 of the Appendix, Table C. The “N<5” should be written “N≤5” (less than or equal to). Pdf and html should be converted to upper case letters (PDF and HTML). X² should be written χ². *F* (Fisher’s statistic) should be italicized.

Changed

On page 15, tables D and E. It is not very clear why the number of data points (number of years) differ between columns (N=35 or N=36). I guess that it could be due to exclusion of years with too few articles for proper estimation of the CR. A footnote could explain that.

I clarified in the legend:

Differences in *N* by different CRs are caused by the exclusion of years with too many articles (<20) or test values (<20) in the respective CR.

On Fig C, Fig D and Fig E. Trends are smooth on this figure. I guess you used non-parametric LOESS regression as in figure 4, but this is not clearly specified. You may add that in the figure legend.

I added to the legend:

… from nonparametric LOESS regressions

On page 18, Tables H and I. Point estimates are correct but standard error and P-values are biased in this table, because it does not take in account the correlation of statistical power within an article. You should either completely remove the standard error and P-values or use a robust variance estimator, such as the clustered covariance sandwich estimator (e.g. vcovCL function in the “sandwich” package of R) or jackknife or bootstrap with at least the article, or even, the whole year, as cluster.

Thanks for the remark, I now calculated cluster robust standard errors and added in the figure legend of both figure H & I:

cluster robust standard errors on the article level in parentheses

On page 20 of the Appendix: “*However, social psychology has, because of its very low statistical power, a particularly large FDR (uninflated or with publication bias).*”. The Fig E seems to show the opposite! The social psychology (solid red/orange line) seems to have the lowest FDR while cognitive psychology (dashed green line) seems to have the highest FDR.

Thanks for the pointer, I replaced social psychology by cognitive psychology.
