# Supplementary material for: Are most published research findings false? Trends in statistical power, publication selection bias, and the false discovery rate in psychology (1975–2017)
Source: PLoS One. 2023 Oct 17;18(10):e0292717. doi: 10.1371/journal.pone.0292717 (PMC10581498; doi:10.1371/journal.pone.0292717)
Supplement: Attachment — Submitted filename: Reviewer comments.docx [file pone.0292717.s004.docx]

Conflicts of interest disclosure:

Although I do not personally know the author, I am an informal member of the Metrics International Forum mentioned in the Acknowledgments.

Comments to the author:

The author shows a large-scale epidemiological review of the literature in psychology and sociology from APA PsycArticles®, a set of 221,554 articles in 119 peer-reviewed journals. The supporting material is extensive, with R scripts, the study database and a large appendix with supplementary tables and figures. Many robustness analyses and simulations have been performed to assess the impact of hypotheses and chose the best models. The subject is relevant in the reproducibility crisis of science.

However, there are serious limitations inherent to the design of the study; some of them cannot be easily fixed. At least, the limitations should be discussed. Some results may have to be changed or removed. The editor should consider the limitations in his acceptation or refusal of the manuscript.

In addition, there are many minor problems with the actual structure of the manuscript that can be fixed easily: better reporting of Methods, with less reliance to Supplementary Information, a structured Discussion, including a paragraph about strengths and limitations, a comparison to the literature and research perspectives. The editorial entitled “The case for structuring the discussion of scientific papers” (BMJ 1999;318:1224) may help the author.

I will first mention major issues, then minor issues.

**Major issues**

1. Hypotheses underlying the estimation of the PIC-specific pooled effects

The statistical power can only be calculated if the effect size (Cohen’s *d*) is known. This effect size was calculated for each large psychology/sociology domain, assuming that all effects were identical within a field based on categories defined by the PsycINFO Classification.

This classification provides broad categories. Ten categories make approximatively 50% (figure taken from the Supplementary Information data.Rdata) of all the analysed research. “Cognitive processes”, “Learning & Memory”, “Cognitive & Perceptual Development”, etc.

First, these categories are far too broad to assume that all effects, in primary, secondary and *post hoc* analyses, are identical. Even within an article, effects of different magnitude are expected to be tested. However, the author pooled all these effects in a meta-analysis model and used the pooled effect as reference for power analyses.

However, the variance of effect sizes is important to take in account. For instance, assuming that the thousands tests were all adequately powered (80% every time) but that half of the effects tested were very small, associated to very large sample sizes (to get 80% power), and half of the effects were very large, associated to small sample sizes (with 80% power), then, the overall statistical power would be 80%, but the methodology of this manuscript would find that half of the studies are underpowered (power ~ 5 or 10%) and half are overpowered (power >99%) with an overall power at 40-50% because it would find an intermediate pooled effect size. Therefore, the pooled effect size is not enough: its variance must be assessed. That is possible thanks to the tau² statistic that meta-analyses model can compute. However, the covariance between the effect size and sample size must be computed to reliably assess the overall statistical power, which seems to be impossible.

Another problem exists. Not all tested hypotheses are true. If half of the hypotheses are true, the pooled effect size would be diluted by a factor two, ie. grossly underestimated. Therefore, the pooled effect size must have a correction factor, based on the hypothetical rate of true hypotheses, which is unknown.

The R code and table S4 show how effect sizes (Cohen’s d) are calculated from P-values. These calculations are only based on the type of statistic (eg. t test) and degrees of freedom. This is at unreliable, if not wrong. Whether paired or two-samples t tests are used is unknown. The group balance of two-samples t tests is also unknown. The formula used by authors is correct only if the test is a two-sample t test and both groups are of equal size. If actual groups are unbalanced, the formula underestimates the effect size. If a one-sample t test is used, the underestimation is even worse, because there are twice as many measurements as degrees of freedom, and measurements are correlated, which is not taken in account by the formula. The same consideration applies to F tests: unbalance or pairing (typically in mixed effects models) greatly change the relationship between the F tests, degrees of freedom and effect sizes. Although slightly different, the problem of group balance and pairing (McNemar) also exists for chi-square tests.

From the study database (data.Rdata), approximatively 25% of all tests are F-tests comparing three groups or more. The concept of Cohen’s d is questionable when there are more than two groups. Although the concept of statistical power remains, pooling three-groups effect sizes with two-groups effect sizes is questionable. Once again, balance between groups and absence of pairing is assumed by author’s calculations.

The manuscript mentions that “*The first indication of publication bias and/ or p-hacking was the large share of statistically significant findings (72.8% at the 5% level) in the examined 170 literature, especially in the context of the low statistical power*.”

In my opinion, that may be mainly explained by the wrong hypotheses underlying the assessment of the effect sizes and statistical power. I think that it is mainly explained by a dispersion of z-values much larger than modelled, because effect sizes are not constant within each research field. Indeed, even if publication bias and p-hacking were massive, it would artificially lead to overestimation of the true effect sizes by the meta-analysis algorithm and so, would not make that much a difference between the computed statistical power (44.6%) and proportion of statistically significant findings (72.8%).

Due to all these limitations, I recommend that the author of this manuscript do not put forward results relying on the pooled effect sizes and that they clearly announce the limitations of their approach.

2. Overinterpretation of publication bias

The publication bias was assessed by a reliable method, independent of the statistical power or distributional assumptions. The ρ statistic (ρ=s/0.5-1) is based on the ratio s between the number of P-values between 0.0396 and 0.05 and P-values between 0.0396 and 0.0626. This seems to have been interpreted as an approximation of the rate of p-hacking/publication bias that transform any non-significant (eg. p=0.30) result to a significant result (eg. p=0.045). I think that this may be a strong overestimation. Indeed, the peak of values between 0.0396 and 0.05 may include p-hacked P-values that were initially at 0.06, but it also includes p-hacked P-values that were between 0.06 and 1.00. Assuming that 10% of tests under null hypothesis with P-value between 0.05 and 1.00 are p-hacked to make them enter the [0.03-0.05] range according to a uniform distribution, there will be 2% of results “naturally” between 0.03 and 0.05, plus 10% artificial. Between 0.0396 and 0.05, there will be approximatively half of them ((10%+2%)=6%), compared to 0.9×1.26%=1.13% between 0.05 and 0.626. The ρ statistic will be 6/(6+1.13)/0.50-1=68.3%, much higher than the 10% of p-hacking of the hypothesis. Unfortunately, the real publication bias or p-Hacking cannot easily be inferred from the ρ statistic because it depends on several unknown parameters. First, the correlation between the P-value and p-hacking. A P-value at 0.07 may be p-hacked more frequently than a P-value at 0.50. Second, the distribution of P-values after p-hacking. What is the proportion between 0.04 and 0.05? Between 0.01 and 0.04? Less than 0.01? Third, the process of the publication or selective reporting bias. The publication bias may be higher for P-values far from 0.05 than P-values close to 0.05. At least, the publication bias follows a more predictable scheme, because it ultimately leads, under the null hypothesis, to a uniform distribution of P-values between 0 and 0.05.

Therefore, the ρ statistic, is a poor estimator of the proportion of null results that are artificially made positive by p-hacking or publication bias. It is used to estimate the FDR_inf_ in Fig 2. I propose to provide sensitivity analyses with different ρ values.

3. The FDR depends on the proportion of true hypotheses

That is well described by the author of this manuscript and several scenarios are tested: 50%, 20% and 10% of true hypotheses. The distribution of P-values may help to assess the FDR. Indeed, if 5% of P-values were below 5%, one could easily tell that most discoveries are false. If 95% of P-values were below 0.0001, that would be indicative of true discoveries. Nevertheless, the difference between p-hacking, publication bias and true discoveries can hardly be made.

4. The difference cannot be made between primary and secondary analyses

Sometimes, some secondary tests have very little power. Sometimes, the null hypothesis is even true, eg. when comparing characteristics of two randomized groups. Other times, secondary tests may be “knocking on an open door”, with a very high power. The results of these tests are of minor interest. They also may violate the hypothesis of “constant effect size” that has been made.

The author approached the concept of “primary analysis” by performing a subgroup analysis of articles with five tests or fewer. I fear that such articles may contain more tests, but the algorithm could not track them because they were not reported with the APA style. May the author verify that fewer than five tests were performed on a subset of articles, by human reading of HTML or PDF files?

I do not think that there is a solution to that problem, but it should be mentioned in the discussion.

**Minor issue: methods**

The R script supports some deviations to APA style but generates wrong results in some cases! Moreover, it may support more deviations.

Examples:

“Chsq(54,N=10,800)” should be interpreted as Chsq(54,N=10800) but has been incorrectly interpreted as Chsq(54,N=10).

“F(4,752,9,504)=13.06” should be considered as ill-formed but has been incorrectly interpreted as F(4,752)=13.06

“F(1,,70)=4.28” has been ignored but could be interpreted as “F(1,70)=4.28”

“Chsq(1,332,N=591)=1673” has been interpreted as “Chsq(1,N=332)=1673” but is ill-formed unless some reliable meaning can be found.

My personal suggestion is to identify well-known patterns with strict regular expressions, then, check what has not matched and find new regular expressions that exclude or correctly interpret the new patterns; then iterate until all tests have been classed in one of the two categories: “well-defined meaning” and “ill-formed”. R warnings should not be ignored.

**Minor issues: reporting of methods**

Although many Supporting Information is provided, the exact references to articles seem to have be anonymized with identifiers such as “E1974_1984#1” containing a period and an index number. Why not providing the Digital Online Identifier (DOI), a Uniform Resource Locator (URL), or an identifier that makes it possible to retrieve the article?

Most of my understanding of the methodology of the article comes from the Supporting Information. The Methods or Result section should contain the following ideas that are required to understand the article:

1) Test statistics were extracted from the full-text of articles by regular expressions
2) Most full-text articles were in HTML format (~96%); but the oldest and newest could be in PDF format; OCR was used in PDF scans.
3) Test statistics in tables were not extracted because they generally do not follow the APA style
4) The APA style is typically test(degrees of freedom)=value where test can be “F”, “Chisq”, “t”.
5) Sample sizes could not be calculated for t tests and F tests; therefore, it was assumed that they were about equal-sized independent groups.
6) Z-tests were ignored.

The Caliper Test (CT) is well described but the conversion from z-values to P-values should be given to make things easier to understand for the reader.

Since effects are actually not Cohen’s d for 3-sample F tests and paired t tests, they should not be called like that. The term “effect size” may be preferred.

Please, clarify inclusion/exclusion criteria. The criteria “*articles that followed the strict reporting guidelines published by the APA in 1974.*” seems to be inconsistent with the Supplementary Information. Indeed, from the R script, it seems that some ill-formed test statistics are found in articles without the whole article being excluded: only the test seems to have been excluded. Moreover, some common deviations to the APA style were correctly detected and interpreted. This is well-specified in the Supplementary Information PDF “Small deviations from the publication manual were also covered.” but contradictory to the inclusion/exclusion criteria.

Additional exclusion criteria were identified in the R script, in a section called “Exclusion criteria”. These should be succinctly reported in the Methods section. Again, they seem to apply to tests and not articles.

The calculation of the False Discovery Rate (FDR) relies on the assumption that all tests are independent. Although this assumption is minor compared to others, it should be mentioned to be rigorous.

A reference to the APA style guideline should be added at the first mention of this style.

**Minor issues: reporting of Results**

Please, add a flow chart to the manuscript, ideally as figure 1. I suggest that both the number of tests and articles be reported in each box of the flow chart. The flow chart should be cited at the first paragraph of the Results section.

It is written that “*Overall, only 17.8% of the studies were adequately powered (>80%), although this share increased to 28.3% in 2017*”. Is 17.8% the proportion of adequately powered **tests** or **studies**? If this is about studies, how are all tests of a single study aggregated for the power calculation?

It is mentioned that “*The replication rate is, however, with 45.6% nearly identical with the statistical power.*” but it is unclear how it has been calculated. Is it calculated for each test, as the probability that a replicated study with the same sample size leads to a significant result conditional to the fact that the first study had a positive result, whatever the null or alternative hypothesis is true? Does it take in account the p-hacking and publication bias in the original study? In the replication study? Does it take in account the Bayesian probability that the alternative hypothesis is true conditional to the success of the first analysis? This should be presented in Methods.

The sentence “*This also applies to the time trend (r = 0.99).*” is unclear. What are the two variables correlated?

The sentence “*Low statistical power also has the deterrent side effect of fostering publication bias or p-hacking because it is harder to detect existing effects. This may encourage authors to engage in publication bias and/ or p-hacking to find an existing effect by repeated testing until they reach statistical significance.*” is inappropriate for a result section and should be moved to the introduction or completely removed, since it does not contain any result.

**Minor issues: Supplementary Information (PDF)**

**Table S7:** the bottom legend of the table suggests that the table contains values of “FDR” but the columns titles, at the top of the table, suggests that the table contains values of “Statistical power”. The consistency should be fixed.

The two following sentences are hard to understand: “*Until the year 1986, all texts retrieved were only available in PDF format. In the following years, this share drastically drops to nearly zero. In the most recent years, 2016 and 2017, 42.2% and 10.6% of the texts were only accessible in PDF format.*”

The second sentence seems contradictory to the first one, but a re-analysis from the study database makes everything clear: The PDF share dropped between 1986 and 2015 and strangely raised in 2016 and 2017. The reason of this raise should be explored: new journals included? Time to convert PDFs to HTMLs? The sentences should be rephrased to make things clearer, such as replacing “*In the following years*” by “*from 1986 to 2015*” and replacing “*In the most recent years, 2016 and 2017*“ by “*This share greatly increased in 2016 and 2017*”.

**Minor issues: introduction**

Please write complete sentences in plain English, avoiding ambiguous parentheses. How can I interpret the sentence: “*Severe misconduct, such as fraudulently making up whole datasets (2), and minor misconduct, such as (deliberately) erroneous rounding results*”. Either remove the word “deliberately”, or remove parentheses or explicitly rephrase “*minor misconduct, such as deliberate or inadvertent erroneous rounding of results*”.

The concept of “selective reporting bias” may be mentioned in addition to publication bias. Although both may be confounded, the publication bias is about not publishing the article at all while the selective reporting bias is about not reporting non-significant P-values in published articles.

Some sentences are awkward, such as “*Because minor forms of scientific misconduct are much more common than fraud (7), they erode scientific integrity in society more severely*”. It basically tells that scientific misconduct (ie. lack of scientific integrity) erodes scientific integrity? That is a tautology.

“*Statistical power [...] is a quality criterion of good research practice and should be as high as possible.*”. Please, either remove the term “*quality criterion*” or “*practice*”. Please, also remove the words “*and should be as high as possible*”, since it is contradictory to the next sentence that mentions that one should “*keep the included number of cases small (e.g. to minimize harm to participants or fit budget constraints)*”.

Assertions without solid data to backup should not be written as assertions. For instance “*Finally, results driven by scientific misconduct shape the public discourse and ultimately drive (political or medical) interventions that might do more harm than good (8).*” is supported by a reference that shows an expert’s opinion without underlying data. The words “and *ultimately drive*” should be replaced by “**may** ultimately drive” or any other formulation that adds uncertainty to the assertion. The last sentence of the introduction “*Instead of rejecting theoretical predictions, so-called "undead theories" with no evidential value survive (24) and inhibit any scientific progress.*” suggests that there is no scientific progress at all due to false undead theories. The word “*any*” should be removed.

Some approximations are done: “*This article shows the development of statistical power, publication bias and p-hacking as forms of scientific misconduct*”. Statistical power is not a form of scientific misconduct. **Insufficient** statistical power may be caused by scientific misconduct, namely by a lack of scientific rigor and methodology in the assessment. However, insufficient statistical power, per se, is not a scientific misconduct.

Some sentences are redundant such as “*From a resource-focused perspective, low statistical power is inefficient because random variations in the data disguise the underlying effect patterns.*” and “*Therefore, the noise of the falsely detected estimates, as defined by the significance threshold (false-positive rate: FPR = e.g. set at 5%), dramatically impacts the overall significant results.*”. The former may be removed without loss of information. The latter could be more rigorous. Indeed, the FPR is not exactly equal to the significance threshold if tests are correlated it could be rephrased to “*(equal to the false-positive rate if tests are independent: FPR = e.g. set at 5%)”* or the FPR could be mentioned in a separate sentence.

A few words should be added to mention the aspects that make this work original compared to previous works.

**Minor issues: discussion**

The discussion is not enough related to the article content. The first paragraph, summarizing the main results is appropriate but may be improved. For instance, the first sentence “*The article has presented evidence on the development of three scientific integrity measures: statistical power, the prevalence of publication bias and p-hacking, and the FDR.*” may be rephrased to suggest that statistical power is a quality criterion of scientific research but not necessarily a measure of scientific integrity. The word “*are*” should be replaced by “could be” in “*The resulting FDRinf illustrates the consequences, as roughly one-third of all significant results are just statistical artifacts*”. There are too many limitations and hypotheses to provide a clear-cut conclusion.

The rest of the discussion makes recommendation based on the author opinion. I suggest to rewrite it entirely, based on BMJ’s guidelines (BMJ 1999;318:1224).

**Suggestions: additional robustness and subgroup analyses**

I am curious to know whether results depend on the type of test (chi-square, t, F, etc.)?

A robustness analysis could be performed to compare the quality of PDF and HTML files. That is especially useful to interpret the latest results (2016 and 2017). Are there the same number of tests detected by article in PDF and HTML files? Are other metrics (power, publication bias, FDR) similar?
